# Tunable magnons in a dual-gated 2D antiferromagnet

**DOI:** 10.1038/s41467-026-74067-z

**Published:** 2026-06-12

**Authors:** Nele Stetzuhn, Abhijeet M. Kumar, Sviatoslav Kovalchuk, Denis Yagodkin, Louis Simon, Samuel Mañas-Valero, Eugenio Coronado, Takashi Taniguchi, Kenji Watanabe, Deepika Gill, Sangeeta Sharma, Piet W. Brouwer, Clemens von Korff Schmising, Stefan Eisebitt, Kirill I. Bolotin

**Affiliations:** 1 Department of Physics, Freie Universität Berlin, Berlin, Germany; 2 Max Born Institute for Nonlinar Optics and Short-Pulse Spectroscopy, Berlin, Germany; 3 Instituto de Ciencia Molecular, Universidad de Valencia, Burjasot, Spain; 4 Department of Quantum Nanoscience, Kavli Institute of Nanoscience, Delft University of Technology, Delft, The Netherlands; 5 National Institute for Materials Science, Tsukuba, Japan; 6 Institut für Optik und Atomare Physik, Technische Universität Berlin, Berlin, Germany; 7 Halle-Berlin-Regensburg Cluster of Excellence CCE, Berlin, Germany

**Keywords:** Magneto-optics, Spintronics, Magnetic properties and materials, Two-dimensional materials

## Abstract

The layered antiferromagnet CrSBr features magnons coupled to other quasiparticles, including excitons and polaritons, which enables their easy optical accessibility. In this work, we investigate the response of the magnons in few-layered devices to changes in carrier density and an applied perpendicular electric field. While the frequencies of both modes increase with the electron density, we reveal their asymmetric response with respect to the electric field. To understand the mechanism of this disparity, we propose a layer-resolved macrospin model describing the magnetic dynamics in thin, non-uniformly doped devices. Through this model we establish the dominant dependencies of the interlayer exchange interaction, magnetic anisotropy, and magnetic moment on the electron density and electric field in individual layers. We demonstrate an on-chip tunability of the in- and out-of-phase magnon frequencies by up to 2 GHz in a dual-gated trilayer device. Our results advance the applications of gate-tunable magnonic devices based on 2D materials.

## Abstract (web summary)

The 2D antiferromagnet CrSBr hosts exciton-coupled coherent spin waves. The authors demonstrate the tunability of these modes by electrostatic gating. With a simple model, the gate dependence of the magnons can be predicted across device thicknesses.

## Introduction

Two-dimensional (anti)ferromagnets have garnered attention as an easily tunable platform to study static and ultrafast magnetism. Thin van der Waals magnets, like other two-dimensional materials, react strongly to external fields^1^, strain^2–7^, intercalation^8^, and the formation of Moiré potentials^9–11^, enabling reversible control of their magnetic properties. The semiconducting layered antiferromagnet CrSBr is of special interest due to its environmental stability^12–14^ and strong anisotropy, stemming from its orthorhombic lattice structure^15^. This anisotropy results in monolayers of CrSBr being easy-axis ferromagnets along the crystallographic $\widehat{b}$-axis, whereas neighboring layers couple antiferromagnetically below the Néel temperature of 132 K ^16^. It also gives rise to a 1D character of the electronic band structure of CrSBr^15^. This, in turn, influences its transport properties^17,18^ as well as the directionality of its tightly bound excitons^19,20^.

Early studies on magnons in CrSBr have demonstrated their long coherence length^21^, broadband tunability^2^ and strong coupling to excitons^21–25^. In contrast to conventional antiferromagnets, in which magnetic order is often elusive to optical probes, this coupling allows one to observe magnon oscillations in absorption-based techniques at the excitonic^21,26^ and polaritonic resonances^23^. The majority of studies on magnons in CrSBr have focused on bulk-like samples so far^21,27,28^. However, a new range of phenomena emerges in thin 2D magnets due to their sensitivity to external perturbations. For instance, the carrier density and electric field in few-layer samples, controlled by external gates, influence material properties such as the coercive field^29^, critical temperature^30,31^ and magnetic ground state^5,32^. While the effect of gating on magnon energies and transport has been studied in a few selected 2D magnetic systems^33–36^, the response of the spin waves in thin CrSBr to electrostatic gating remains unexplored.

Indeed, characterizing the response of a dual-gated 2D antiferromagnet is challenging within the framework of current models: To describe magnetic excitations in bulk-like layered antiferromagnets, one commonly uses a macrospin model in which all spins within one layer are locked by the strong intralayer exchange interaction^37,38^. The complete antiferromagnetic bulk can then be described by only two coupled macrospins. In thin layered systems, however, an out-of-plane electric field leads to inhomogeneous doping across the device, breaking the symmetry between the layers. This leads to several questions about the detailed microscopic mechanisms governing magnon dynamics: How does the presence of free carriers affect the magnetic moment, interlayer exchange and magnetic anisotropies in CrSBr? What is the role of differing magnetic properties in individual layers as a result of electric-field-induced symmetry-breaking? Can a layer-resolved macrospin model describe the resulting magnonic behavior?

In this report, we address these questions by exploring the response of magnons in few-layer CrSBr devices to electrostatic gating. We find an opposing trend of the in- and out-of-phase magnon mode in response to an electric field in a dual-gated trilayer device, which, to the best of our knowledge, has not been reported previously. The detailed understanding of such a response is especially important in the context of developing spintronic applications of nanostructured 2D materials^39^. We therefore develop a minimal model connecting the layer-resolved charge carrier densities and electric fields to the magnetic properties of the sample. This allows us to explicitly correlate the measured magnon frequencies in CrSBr to its electrostatic properties using capacitive modeling and gate-dependent photoluminescence measurements.

## Results

We utilize time-resolved (tr) reflectivity to investigate the gate-dependent behavior of magnons in CrSBr (see methods). The excitons in CrSBr exhibit a strong coupling to the magnetic order of the sample: Depending on the orientation of the macrospins in neighboring layers, the interlayer character and degree of localization of the excitons differs^40–42^. This results in a change of their binding energy, and accordingly their energetic resonance position in photoluminescence and reflectivity spectra^22,23,25^. Coherent magnons can therefore be seen as oscillations in the tr-reflectivity signal of the sample when probing close to an excitonic resonance^21,26,28^.

**Fig. 1:**
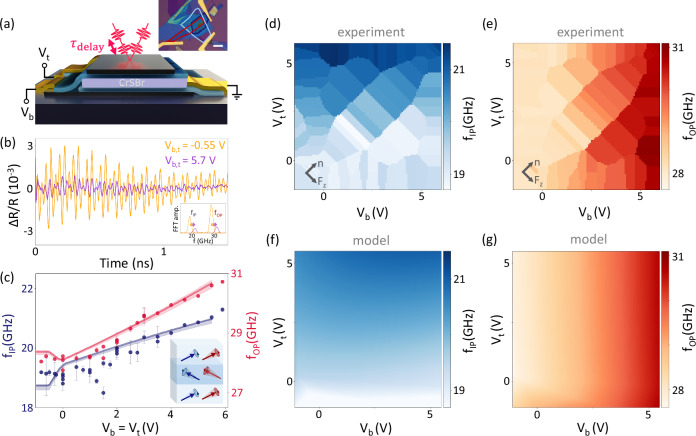
Magnons in dual-gated trilayer CrSBr. **a** Device scheme of a trilayer CrSBr flake (purple) encapsulated in hBN (light blue) with a top and bottom graphite gate (gray). The magnons are excited with an ultrafast pump laser pulse and read out by a probe pulse after *τ*_delay_. The inset shows an optical image of the trilayer device (red: CrSBr flake, blue: top and bottom graphite), the scale bar corresponds to 10 μm. **b** Two exemplary time-resolved reflectivity traces after background subtraction, for weak (*V*_b_ = *V*_t_ = − 0.55 V, orange) and strong (*V*_b_ = *V*_t_ = 5.7 V, violet) electron doping. The drop in signal intensity stems from the doping-dependent changes in the tr-reflectivity spectra (see Supplementary Fig. 3). An in-phase mode *f*_IP_ and an out-of-phase mode *f*_OP_ are extracted from the traces using a Fast Fourier Transform, shown in the inset. Electron doping shifts the magnon frequencies from 19.1 to 21.1 GHz and 28.1 to 30.8 GHz, respectively. **c** The complete doping response (*V*_b_ = *V*_t_) of *f*_IP_ (blue, see inset) and *f*_OP_ (red, see inset) shows that the frequency increase starts after a threshold voltage. The errorbars are showing the uncertainty when extracting the peak position from the FFT. The solid lines are fits using the layer-resolved macrospin model. The shaded regions are calculated with the model using the errors of the fitting parameters given in the first row of Table 1. The complete *V*_b_-*V*_t_-dependencies of **d** *f*_IP_ and **e** *f*_OP_ plotted as Voronoi diagrams show a complex response to gating, which is opposite with electric field for the two modes (arrows indicate increasing electron density *n* and electric field *F*_z_). Using a layer-dependent macrospin model connected to an electrostatic model we can reproduce the experimental trends in the fitted *V*_b_-*V*_t_-diagrams for both **f** *f*_IP_ and **g** *f*_OP_.

The experimental platform to control electric field and doping consists of a thin CrSBr flake (2 − 8 layers, AFM in Supplementary Note 1) sandwiched between two graphite gates, each separated from the CrSBr by few-layered hBN (5 − 11 nm), as shown in Fig. 1a. The CrSBr is electrically grounded throughout the experiment, while the gate voltages *V*_b_ and *V*_t_ are separately applied to bottom and top graphite, respectively. In this scheme, the sum of gate voltages *V*_b_ + *V*_t_ shifts the Fermi level in CrSBr, consequently changing its doping, while the difference between gate voltages *ΔV* = *V*_t_ − *V*_b_ controls the strength of a perpendicular electric field through the device. All measurements were performed at *T* = 10 K, well below the Néel temperature (temperature-dependent data in Supplementary Note 2). The magnons are excited with an ultrafast laser pulse tuned to the exciton or trion resonance at 1.376 eV or 1.350 eV, respectively. Subsequently, we probe the tr-reflectivity response at the same wavelength. We tune the pump- and probe energy to either resonance depending on the doping level of the system to optimize the signal-to-noise ratio in the time-resolved reflectivity measurements. To enhance the signal strength, we apply a constant external magnetic field *H*_0_ ≈ 0.1 T using a permanent magnet in proximity of the sample (Supplementary Note 3).

First, we explore the effect of dual-gating in a trilayer of CrSBr. In Fig. 1b, we observe pronounced oscillations in the tr-reflectivity traces for different values of *V*_b_ = *V*_t_ (orange: −0.55 V, purple: 5.7 V; exponential background subtracted from both). The oscillation amplitude decreases going from low (orange) to high (purple) gate voltages, as the spectral weight between exciton and trion resonance depends on the sample doping. The two oscillation frequencies around 19.1 ± 0.1 GHz and 28.1 ± 0.1 GHz (*V*_b_ = *V*_t_ = −0.55 V, inset of Fig. 1b) correspond to the in-phase and out-of-phase zero momentum magnon modes *f*_IP_ and *f*_OP_, respectively, in accordance with previous reports^21,26,28,43^. As seen in Fig. 1c, increasing the gate voltages leads to an upshift of the frequencies of both modes, to a maximum of *f*_IP_ = 21.1 ± 0.1 GHz and *f*_OP_ = 30.8 ± 0.1 GHz (*V*_b_ = *V*_t_ = 5.7 V, inset of Fig. 1b). Notably, this shift is nonlinear – in particular, below *V*_t_ = *V*_b_ ≈ 1 V, there is no significant change from the initial frequencies. In the complete *V*_b_-*V*_t_-dependence of *f*_IP_ and *f*_OP_ shown in Fig. 1d, e, we observe an asymmetry between the two modes in response to the perpendicular electric field *F*_z_ (arrows mark the direction of increase in doping *n* or field *F*_z_). Specifically, *f*_IP_ increases mainly with *V*_t_, while *f*_OP_ reacts to changes in *V*_b_. In the following, we develop a microscopic picture to describe these dependencies.

**Fig. 2:**
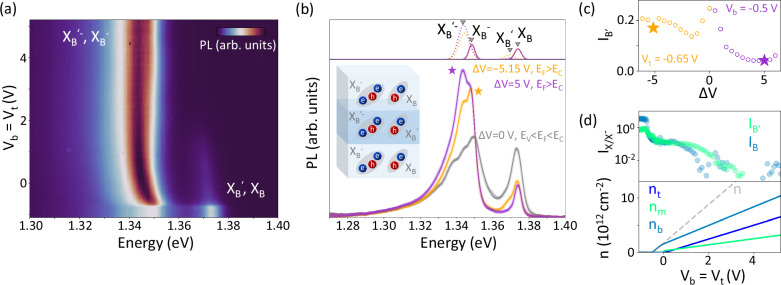
Dual-gate dependence of photoluminescence in trilayer CrSBr. **a** Doping-dependent PL map of the trilayer CrSBr showing the dimming of the neutral exitons *X*_B_ and ${X}_{{{\rm{B}}}}^{{\prime} }$ around ~1.37 eV and brightening of the trions ${X}_{{{\rm{B}}}}^{-}$ and ${X}_{{{\rm{B}}}}^{{\prime} -}$ at ~1.34 eV with increased electron density. **b** Exemplary PL spectra for undoped (gray) and doped (orange, purple) trilayer CrSBr (dots are datapoints, solid lines are fits using a sum of Gaussians). We assume a gate-independent background in the energy range between 1.32 and 1.34 eV to account for contributions of, e.g., *X*_A_ and phonon replicas of *X*_B_ in the fits. The upper panel shows the fitted exciton and trion peaks for the doped spectra. Both the exciton and trion peaks are split in two due to the complexes residing in the outer (B, solid lines) vs. the middle layer (B', dashed lines), as shown in the inset sketch. Under the same overall electron density *n* in the sample, but opposite electric field polarity ∝*ΔV* (orange: *ΔV* < 0, purple: *ΔV* > 0), the exciton-to-trion ratio *I*_B_ is almost identical (compare orange and purple solid exciton and trion fits in upper panel), while ${I}_{\,{{\rm{B}}}}^{{\prime} }$ is sensitive to the field direction (compare orange and purple dashed exciton and trion fits in the upper panel). **c** Extracted ${I}_{\,{{\rm{B}}}}^{{\prime} }$ for fixed *V*_t_ = −0.65 V (orange) or fixed *V*_b_ = −0.5 V (purple), but varying *ΔV* = *V*_t_ − *V*_b_ (spectra from (**b**) marked with stars). For the same doping level (graph is symmetric with doping), the ratio is lower for a positive *ΔV*, due to a higher electron density *n*_m_ for positive field direction. **d** Extracted doping-dependent exciton-to-trion ratios *I*_B_ and ${I}_{\,{{\rm{B}}}}^{{\prime} }$ from (**a**) in the top panel, and layer-resolved carrier densities from electrostatic modeling in the lower panel. The drop of the exciton-to-trion ratios is closely followed by the modeled increase of *n* (gray).

To understand the mechanism behind the gate-dependent magnon frequencies, it is crucial to examine the carrier density configuration of our device. Generally, the ratio of intensities between neutral and charged excitons in gate-dependent photoluminescence (PL) measurements can be used as a proxy for carrier density in 2D materials^44–46^. In Fig. 2a, we plot the PL map of the trilayer CrSBr device as a function of gate voltage *V*_b_ = *V*_t_ (*ΔV* = 0). We see two main features in the energy ranges around 1.34 eV and 1.37 eV, which we assign to trions and excitons, respectively^47^. With increasing voltage, the intensity of the trion emission at 1.34 eV increases abruptly at *V*_b_ = *V*_t_ ≈ − 0.8 V, concurrently with a dimming of the excitonic peak at 1.37 eV. This is consistent with the neutral excitons binding to additional electrons, and suggests an intrinsic n-doping of our sample (as the trions dominate the spectrum at *V*_b_ = *V*_t_ = 0 V). Therefore, we conclude that below *V*_b_ = *V*_t_ ≈ − 0.8 V, the Fermi level lies in the bandgap and the sample is not significantly doped.

In Fig. 2b, the gray data points show an exemplary PL spectrum of the trilayer CrSBr device in this regime without applied field, i.e., *E*_V_ < *E*_F_ < *E*_C_ (where *E*_V_ and *E*_C_ are the valence and conduction band energies, respectively), and *ΔV* = 0 V. We observe strong emission at *X*_B_ ≈ 1.374 eV with a shoulder at lower energies stemming from ${X}_{{{\rm{B}}}}^{{\prime} }\approx 1.370\,{{\rm{eV}}}$. In previous reports, *X*_B_ and ${X}_{{{\rm{B}}}}^{{\prime} }$ were assigned to the excitonic transition between top valence and second lowest conduction band^15,22,47,48^. Their energetic splitting in samples with > 2 layers has been attributed to different dielectric screening in the outer (B) versus inner (B’) layers, leading to a layer-resolved response of thin samples to electrostatic gating^47^. Around 1.34 eV, we find an amalgamation of several peaks even in the absence of trions (as *E*_V_ < *E*_F_ < *E*_C_), which can be assigned to the *X*_*A*_ exciton (transition between top valence and bottom conduction band) and phonon replicas^15,22,47,48^. We note that a different interpretation of the emission at 1.34 and 1.37 eV has recently been given, in which the latter has been attributed to surface and the former to bulk excitons^49^. However, the thickness-dependent relative intensities of *X*_B_ and ${X}_{{{\rm{B}}}}^{{\prime} }$ observed in our experiments support their assignment as excitons in the outer and inner layers (gate-dependent PL of samples with varying thickness in Supplementary Note 4).

For sufficiently high electron doping (*E*_F_ > *E*_C_), we see emission from trions at ${X}_{{{\rm{B}}}}^{-}\approx 1.348\,{{\rm{eV}}}$ and ${X}_{{{\rm{B}}}}^{{\prime} -}\approx 1.344\,{{\rm{eV}}}$ (orange and purple spectra in Fig. 2b, fitted exciton and trion peaks in the upper panel). We note that the binding energies of the outer and inner layer trions ${X}_{{{\rm{B}}}}^{-}-{X}_{{{\rm{B}}}}={X}_{{{\rm{B}}}}^{{\prime} -}-{X}_{{{\rm{B}}}}^{{\prime} }=26\,{{\rm{meV}}}$ match, confirming the assignment of these peaks. Therefore, we use the relative intensities ${I}_{{{\rm{B}}}}=\frac{{I}_{{X}_{{{\rm{B}}}}}}{{I}_{{X}_{{{\rm{B}}}}^{-}}}$ and ${I}_{{{{\rm{B}}}}^{{\prime} }}=\frac{{I}_{{X}_{{{\rm{B}}}}^{{\prime} }}}{{I}_{{X}_{{{\rm{B}}}}^{{\prime} -}}}$ as indicators for the carrier densities of the outer layers, *n*_*t*_ + *n*_*b*_, and the middle layer, *n*_*m*_, respectively. Field-dependent measurements confirm this assignment: In Fig. 2b, we show two selected PL spectra for opposite fields *F*_z_ ∝ *ΔV*, but equivalent doping level *n* (orange: *ΔV* < 0, purple: *ΔV* > 0). While the spectral weight *I*_B_ is nearly identical for the two (as either top or bottom layer become doped), ${I}_{{{{\rm{B}}}}^{{\prime} }}$ differs for *ΔV* > 0 and *ΔV* < 0 (compare the relative intensities of excitons and trions in the upper panel of Fig. 2b). Figure 2c shows the complete dependence of ${I}_{{{{\rm{B}}}}^{{\prime} }}$ on *ΔV*, in which the points at *ΔV* and  − *ΔV* have roughly equivalent doping (full gate-dependence of *I*_B_, ${I}_{{{{\rm{B}}}}^{{\prime} }}$ in Supplementary Note 4). Generally, the intensity ${I}_{{{{\rm{B}}}}^{{\prime} }}$ is smaller for positive than negative polarity of *ΔV*, which suggests a built-in electrical field *F*_z,0_, possibly originating from surface charges^13^.

We quantitatively link *I*_B_ and ${I}_{{{{\rm{B}}}}^{{\prime} }}$ to the layer-resolved electron densities *n*_*i*_ and electric fields *F*_z,*ij*_ (where *i*, *j* = top, middle or bottom layer) using a simple electrostatic model^50,51^. For this, we assume that the interlayer character of the excitons is sufficiently small, because the weak applied external magnetic field only results in a small canting angle of the in-plane magnetization^42^. The main free parameters of the capacitive model are the built-in field *F*_z,0_ and the carrier density *n*_0_ at *V*_b_ = *V*_t_ = 0 V. The former leads to an offset between the bands of the neighboring layers, and the latter defines the initial position of the chemical potential. For the behavior of the layer-resolved carrier densities to resemble the experimentally obtained PL data, we arrive at *n*_0_ ≈ 1.8 × 10^12^ cm^−2^ and *F*_z,0_ ≈ − 0.02 V nm^−1^ (details of the model and gating maps of *n*_*i*_ and *F*_z,*ij*_ in Supplementary Note 4). Comparing the resulting *n*_*i*_ as a function of *V*_b_ = *V*_t_ (Fig. 2d, lower panel) to the ratios *I*_B_ and ${I}_{{{{\rm{B}}}}^{{\prime} }}$ extracted from Fig. 2a (Fig. 2d, upper panel), we find a reasonable alignment of the drop in both exciton-to-trion ratios around ~−0.8 V with the onset of doping between ~− 0.5 and 0 V. The magnon frequencies in Fig. 1c follow the doping-dependence of the total carrier density of all three layers *n* = ∑_*i*_*n*_*i*_ (Fig. 2d bottom, gray dashed line) with a small offset in the gate voltage. This suggests that carrier-density induced modifications of magnetic properties contribute to the changes in magnon dynamics. However, the asymmetry with *ΔV* shown in the full gating maps of *f*_IP_ and *f*_OP_ in Fig. 1d, e cannot be explained by changes in *n* alone, indicating a dependence on the electric fields *F*_z_, as well.

To explain how a gate-dependent layer asymmetry influences the magnonic behavior, we use a layer-resolved macrospin model. This approximation assumes strongly coupled spins within individual layers, such that their collective motion can be described by a single macrospin **m**_*i*_. Due to the long magnon lifetime (>900 ps) observed in Fig. 1b, and because we are mainly interested in the magnon frequencies, we omit damping effects. The dynamics of each macrospin are then governed by the layer-dependent effective field **H**_eff,*i*_ in the Landau–Lifshitz (LL) equation:

1 $$\frac{{{\rm{d}}}{{{\bf{m}}}}_{i}}{{{\rm{dt}}}}=-\gamma {{{\bf{m}}}}_{i}\times {{{\bf{H}}}}_{{{\rm{eff}}},i}.$$

Here, *γ* is the gyromagnetic ratio and ${{{\bf{m}}}}_{i}={M}_{{{\rm{0}}},i}{{\widehat{{{\bf{m}}}}}}_{i}$ is the layer-dependent macrospin. The effective field is given by

2 $${{{\bf{H}}}}_{{{{\rm{eff}}}}_{i}}=-{\nabla }_{{{{\bf{m}}}}_{i}}E={{{\bf{H}}}}_{0}-{\sum }_{ < i,j > }{H}_{{{{\rm{E}}}}_{i\, j}}{{{\bf{m}}}}_{j}+{H}_{{{{\rm{a}}}}_{i}}{m}_{i}^{{{\rm{a}}}}{\widehat{{{\bf{a}}}}}+{H}_{b}{m}_{i}^{{{\rm{b}}}}{\widehat{{{\bf{b}}}}},$$

with the external field **H**_0_, the interlayer exchange coupling ${H}_{{{{\rm{E}}}}_{ij}}$ between neighboring layers *i* and *j*, and two anisotropies *H*_b_ and ${H}_{{{{\rm{a}}}}_{i}}$ along the easy $\widehat{{{\bf{b}}}}$- and intermediate $\widehat{{{\bf{a}}}}$-axis. The macrospin components ${m}_{i}^{{{\rm{a}}}}$ and ${m}_{i}^{{{\rm{b}}}}$ point in those same directions. In contrast to conventional models, the equilibrium macrospin, interlayer exchange and intermediate axis anisotropy are taken to be explicitly layer-dependent. To solve Eq. (1), the external field of *H*_0_ ≈ 0.1 T is taken to be purely in the out-of-plane direction. The excursions *δ***m**_*i*_ of the macrospins away from the equilibrium **m**_*i*,0_ are assumed small, allowing linearization of the LL equation. We then extract the magnon frequencies numerically.

**Fig. 3:**
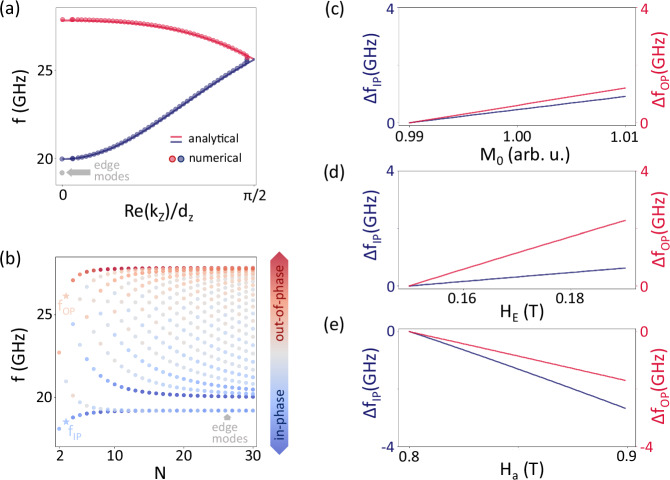
Layer-resolved macrospin model. **a** Comparison of the magnon dispersion of *k*_z_ (*k*_x_ = *k*_y_ = 0; *d*_z_ is interlayer spacing) calculated with the analytical bulk model (solid lines) and the layer-resolved numerical model for 100 layers (dots). Both models show an acoustic (blue) and an optical (red) magnon branch. The edge modes (gray) in the layer-resolved model are a result of the open boundary conditions. **b** Numerical magnon frequencies as a function of layer number *N* calculated with the layer-resolved model. The color-coding shows the efficiency of the coupling of the modes to in- or out-of-phase excitations (details in Supplementary Note 7). For high *N*, the degenerate edge modes lie ≈1 GHz below the bulk modes of the acoustic branch. For *N* < 10, the edge modes split. For *N* = 3, we identify the lowest and highest eigenvalues as the experimental *f*_IP_ and *f*_OP_, respectively (marked by stars). Influence of changing the **c** length of the macrospin *M*_0_, **d** interlayer exchange *H*_E_, or **e** anisotropy *H*_a_ in all layers in the layer-resolved model. Both frequencies shift with *M*_0_ at approximately the same rate, while their sensitivity to *H*_a_ and *H*_E_ differs. For all plots, the respective fixed parameters are *γ* = 155 GHz T^−1^, *H*_b_ = 1.3 T, *H*_a_ = 0.9 T, and *H*_E_ = 0.15 T (0.3 T in the analytical bulk model in (**a**)).

To ensure the validity of the layer-resolved model, it is instructive to compare the numerical dispersion in the case of a bulk-like (*N* = 100 layers) sample to the analytical solution routinely used to model the bulk case (details in Supplementary Notes 5 and 6). In Fig. 3a, we show that the numerical model gives a low-frequency acoustic (blue points) and high-frequency optical (red points) magnonic branch consisting of bulk modes, in good agreement with the analytical results (solid line). The acoustic and optical modes at *k*_*z*_ = 0 correspond to *f*_IP_ and *f*_OP_, and were excited and detected in previous pump-probe experiments^21^. Remarkably, two additional degenerate modes with low frequency emerge in the numerical model (marked gray in Fig. 3a). These are edge modes with an imaginary *k*_*z*_, which decay exponentially into the bulk sample. They are a direct result of the open boundary conditions used (see Supplementary Fig. 11).

In thin samples, the magnon modes predicted by the layer-resolved model begin to deviate from those seen in the bulk. Figure 3b shows the magnon frequencies as a function of device thickness (expressed in layer number *N*). For *N* > 10, the eigenfrequencies of the bulk modes (between ~20 and 28 GHz) as well as the edge modes (below ~20 GHz) remain nearly thickness-independent, in agreement with previous experimental reports^21^. When reducing the layer number to *N* < 10, the maximum and minimum frequencies of the bulk modes start to deviate from the large *N* case. We assign this to the stronger influence of the edge layers, which experience a smaller interlayer exchange coupling. Additionally, the edge modes become non-degenerate and change their character, spreading across the whole sample instead of being localized at one side. In trilayers, we find that only the highest and lowest frequency magnons couple efficiently to a photothermal excitation, so we consider these modes as *f*_OP_ and *f*_IP_ in our experiments (extended discussion in Supplementary Note 7).

Having established the layer-resolved magnetic properties, we now turn to explaining the *V*_b_-*V*_t_-dependent behavior of *f*_IP_ and *f*_OP_ (Fig. 1d, e). In general, various physical processes can result in carrier-density or electric-field dependencies of the parameters in Eqs. (1) and (2) (Supplementary Note 8). To determine compatible mechanisms, we analyze the dependence of the magnon modes on overall changes in *M*_0_( = *M*_0,t_ = *M*_0,m_ = *M*_0,b_), ${H}_{{{\rm{a}}}}(={H}_{{{{\rm{a}}}}_{{{\rm{t}}}}}={H}_{{{{\rm{a}}}}_{{{\rm{m}}}}}={H}_{{{{\rm{a}}}}_{{{\rm{b}}}}})$ and ${H}_{{{\rm{E}}}}(={H}_{{{{\rm{E}}}}_{{{\rm{tm}}}}}={H}_{{{{\rm{E}}}}_{{{\rm{mb}}}}})$ in the numerical model within realistic ranges, see Fig. 3c–e. We observe that both *f*_IP_ and *f*_OP_ increase linearly with *M*_0_ at similar rates (Fig. 3c). Both frequencies also increase with *H*_E_, however at different rates, with a stronger influence on *f*_OP_ (Fig. 3d). Conversely, an increasing *H*_a_ leads to a decrease in both *f*_IP_ and *f*_OP_, with a weaker effect on the latter (Fig. 3e). We only include one of the two anisotropies to be layer-dependent, as increasing *H*_a_ has a similar influence on the frequencies as decreasing *H*_b_ (Supplementary Fig. 10).

We now want to identify the dominating dependencies of *M*_0,*i*_, *H*_a,*i*_ and ${H}_{{{{\rm{E}}}}_{ij}}$ on *n*_*i*_ and *F*_z,*ij*_ to reproduce the behavior in Fig. 1d, e. To this end, we compare different fitting approaches in Supplementary Note 9. From these considerations, we find that the experimentally observed shift of *f*_IP_ and *f*_OP_ at the same rate with respect to carrier density in Fig. 1c could be explained by a dependence of *M*_0,*i*_ on *n*_*i*_. In contrast, the asymmetric shifts of *f*_IP_ and *f*_OP_ vs. applied field in Fig. 1d, e point towards a competing field dependence of two parameters. Considering Fig. 3d, e, we suggest a linear dependence of both ${H}_{{{{\rm{a}}}}_{i}}$ and ${H}_{{{{\rm{E}}}}_{ij}}$ on *F*_z,*ij*_. We therefore arrive at the following minimal set of dependencies:

3 $${H}_{{{{\rm{E}}}}_{ij}}={H}_{{E}_{0}}+{\nu }_{{{\rm{E}}}}{F}_{{{\rm{z}}},ij}$$

4 $${H}_{{{{\rm{a}}}}_{i}}={H}_{{{{\rm{a}}}}_{0}}+{\nu }_{{{\rm{a}}}}{\sum }_{\langle i,j\rangle }{F}_{{{\rm{z}}},ij}$$

5 $${M}_{{{\rm{0}}},i}=1+{\eta }_{{{\rm{M}}}}{n}_{i}$$

where *ν*_E_ and *ν*_a_ are the fitting parameters we use to describe the electric-field dependencies of the interlayer exchange and anisotropy fields, respectively, and *η*_M_ is used to fit the doping-dependence of the layer macrospin. We fit the frequency gating maps in Fig. 1d, e to our layer-dependent LL model with Eqs. (3–5) using the *n*_*i*_ and *F*_z,*ij*_ gating maps from the electrostatic model in Supplementary Fig. 6. The resulting fit parameters are summarized in the first row of Table 1. To estimate the error in the fit parameters of the trilayer data, we vary the fixed parameter *γ* = 155(15) GHz T^−1^ within the range of previously reported values^21^. In the doping-dependency of the modes in Fig. 1c, the model results (solid lines) show good agreement with the data, apart from slight deviations in the low carrier density regime. The modeled frequency maps shown in Fig. 1f, g also reproduce all basic trends of the experimental results reasonably well, in particular the opposing *V*_t_- and *V*_b_-sensitivity of the two magnon modes. To model this behavior, it is necessary to include the sign of the electric field in Eqs. (3) and (4)—which is surprising considering the symmetrical nature of the device stack.

**Table 1:** Macrospin model fit results for devices with different CrSBr thicknesses

thickness	${H}_{{{{\rm{E}}}}_{0}}$ (T)	${H}_{{{{\rm{a}}}}_{0}}$ (T)	*H*_b_ (T)	*ν*_a_ (T nmV^−1^)	*ν*_E_ (T nmV^−1^)	*η*_M_ (10^−12^ cm^2^)
trilayer	0.163 ± 0.003	1.15 ± 0.2	1.48 ± 0.2	−0.86 ± 0.1	−0.85 ± 0.1	(7.3 ± 0.1) ⋅ 10^−3^
bilayer 1	0.50 ± 0.01	0.98 ± 0.02	1.22 ± 0.01			
bilayer 2	0.67 ± 0.03	0.92 ± 0.02	1.16 ± 0.01			
5 layers	0.295 ± 0.005	0.96 ± 0.1	1.27 ± 0.01			
8 layers	0.25 ± 0.01	0.91 ± 0.01	1.16 ± 0.01			

This table summarizes the macrospin model fit parameters for a range of samples. The gyromagnetic ratio *γ* = 155 GHz T^−1^ is fixed in the model. ${H}_{{{{\rm{E}}}}_{0}}$, ${H}_{{{{\rm{a}}}}_{0}}$ and *H*_b_ are the internal fields in Eq. (2), and are fitted for each sample individually. The parameters *ν*_E_ and *ν*_a_ describe the electric-field dependencies of the exchange and anisotropy fields, respectively, and *η*_M_ is used to fit the doping-dependence of the layer macrospin, as described in Eqs. (3 to 5). We fitted all the parameters using the trilayer CrSBr data (resulting *V*_b_-*V*_t_-maps in Fig. 1f, g), with the results shown in the first row. To estimate the errors for the parameters found for the trilayer, the gyromagnetic ratio was varied within *γ* = 155(15) GHz T^−1^. For the 2-, 5-, and 8-layer CrSBr devices, the experimental frequencies were fitted to the macrospin model with *ν*_a_, *ν*_E_, and *η*_M_ fixed to the trilayer values in the top row. The resulting ${H}_{{{{\rm{a}}}}_{0}}$, *H*_b_ and ${H}_{{{{\rm{E}}}}_{0}}$ differ slightly between samples. The errors of the free fit parameters of the 2-, 5-, and 8-layer results are estimated by varying *ν*_a_, *ν*_E_, and *η*_M_ within their error ranges.

The remaining discrepancies between modeled and experimental results stem from the sheer complexity of possible doping- and electric-field-related effects on magnetic properties. First, the position of the chemical potential—controlled by *n*—affects the imbalance between the occupied spin-up and spin-down states, affecting the magnetic moment *μ* and, in turn, the length of the layer macrospin *M*_0_^52^. In addition, an *n*-dependent increase of the Coulomb repulsion influences the antiferromagnetic exchange constant^47,53,54^. Second, the relative population of states derived from *e*_*g*_ to *t*_2*g*_ orbitals in one layer is affected by both chemical potential and external field. This leads to a corresponding dependence of both exchange^53–55^ and anisotropy terms^14,56–58^. Third, a perpendicular electric field shifts the energy bands associated with different orbitals in neighboring layers with respect to each other, again influencing the exchange coupling parameter^59^.

We believe that the suggested dependencies in Eqs. (3) to (5) are the dominating mechanisms of the gate dependency of magnons in CrSBr. The inclusion of, e.g., additional terms such as ${H}_{{{{\rm{E}}}}_{ij}}\propto {n}_{i}$, has not significantly improved the modeling of the experimental data. We also neglect any nonlinear or saturation effects. For the trilayer sample, we predict a change in ${H}_{{{{\rm{E}}}}_{ij}}$ between neighboring layers of up to ~100 mT as a result of the applied electric field, while the tunability of ${H}_{{{{\rm{a}}}}_{i}}$ reaches almost ~200 mT. These changes of effective fields are an order of magnitude stronger than the previously reported gate-tunable internal field in a 10 nm thick Cr_2_Ge_2_Te_6_ sample^35^ and surpass the voltage-controlled magnetic anisotropy induced, for example, in CoFeB samples^58,60,61^. As *M*_0,*i*_ ∝ *n*_*i*_, the macrospin length changes most significantly in the bottom layer due to its strong doping, increasing by  ~ 8% (complete gating maps of *γ*_*i*_, ${H}_{{{{\rm{a}}}}_{i}}$, ${H}_{{{{\rm{E}}}}_{ij}}$ and discussion in Supplementary Note 9).

**Fig. 4:**
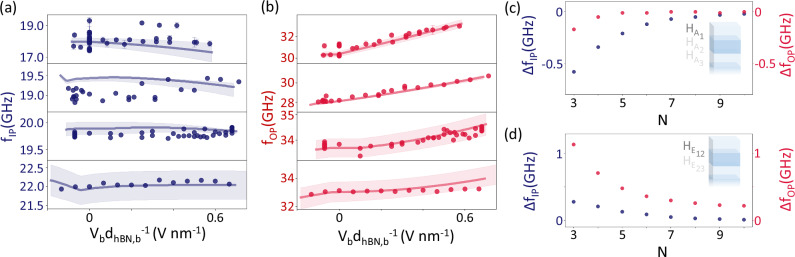
Thickness-dependence of magnon tunability. Comparison of the $\frac{{V}_{{{\rm{b}}}}}{{d}_{{{\rm{hBN,b}}}}}$-dependency of **a** *f*_IP_ and **b** *f*_OP_ for two, three, five, and eight layers of CrSBr. We scale the bottom gate voltage by the bottom hBN thickness *d*_hBN,b_ for better comparability across the devices with different *d*_hBN,b_. The solid lines and the shaded error regions show the frequencies fitted with the macrospin model (parameters in Table 1). In (**a**), *f*_IP_ is insensitive to the bottom gate across all devices, while the tunability of *f*_OP_ in (**b**) drops with layer number. Note the difference in frequency scales for better visibility of the small shift in the thick devices. **c** Modeled shifts *Δf*_IP_ and *Δf*_OP_ when changing ${H}_{{{{\rm{a}}}}_{1}}$ from 0.8 to 0.9 T (${H}_{{{{\rm{a}}}}_{i}}$ of all other layers fixed to 0.9 T, H_b_ = 1.3 T, H_E_ = 0.15 T) as a function of layer number *N*. The shift drops to almost 0 for both modes in 10 layers. **d** Varying ${H}_{{E}_{12}}$ from 0.15 to 0.19 T (other exchange couplings fixed to 0.15 T, H_a_ = 0.8 T, H_b_ = 1.3 T) also results in smaller *Δf*_IP_ and *Δf*_OP_ for higher *N*.

Additionally, the *n*_*i*_- and *F*_z,*ij*_-dependencies found for the trilayer predict the gate-dependent magnon frequency changes in samples of different thickness. The *V*_b_-dependence of *f*_IP_ and *f*_OP_ in a 2, 5-, and 8-layer device, shown in comparison with the previous trilayer data, is plotted in Fig. 4a, b (additional data in Supplementary Note 10). In the bilayer device, we reach a similar tunability to that of the trilayer (in a dual-gated bilayer, we reach our maximum *Δf*_OP_ ≈ 4 GHz, Supplementary Fig. 18). The magnitude of the gating effects decreases with thickness: In the 5-layer device, the maximum shift of *f*_OP_ is only 0.5 GHz, while the 8-layer device shows no discernible frequency shift. All devices also reproduce the insensitivity of *f*_IP_ to *V*_b_ observed in the trilayer. Using *ν*_a_, *ν*_E_, and *η*_M_ extracted from the trilayer data, we model the expected frequency shifts in the other devices. The fitted *H*_b_, ${H}_{{{{\rm{a}}}}_{0}}$ and ${H}_{{{{\rm{E}}}}_{0}}$ differ from the trilayer results (values in Table 1, errors of the free fit parameters estimated by varying *ν*_a_, *ν*_E_ and *η*_M_ within their error interval). The modeled gate-dependent changes (solid lines in Fig. 4a, b with shaded error regions) reproduce the data reasonably well, and agree with our experimental observations of reduced gate-sensitivity with higher layer number (see also Supplementary Fig. 17). In thicker devices, both carrier density and fields are induced mainly around the outer layers, with the inner ones being screened from the gates (electrostatic modeling for 2-, 5- and 8-layer device in Supplementary Note 4). The influence of these outer layers on the magnon frequencies decreases with increasing layer number *N*, illustrated in Fig. 4c, d. The magnon frequency shifts due to changes of the anisotropy (Fig. 4c) or interlayer exchange interaction (Fig. 4d) for one outer layer decrease drastically going from three to ten layers in the layer-resolved model.

## Discussion

In summary, we have demonstrated the gate-tunability of the in- and out-of-phase magnon modes in trilayer CrSBr by up to 10 %. By using the contrasting response of the two magnons to the perpendicular electric field, their separate control is possible. We explain this gate-dependence in a Landau-Lifshitz model with layer-dependent magnetic moment, interlayer exchange, and anisotropy. By coupling the interlayer exchange and intermediate-axis anisotropy to the electric fields and the magnetic moment to the electron densities across layers, we arrive at a minimal model for our experimental results. The model explains most trends in the data, including the contrasting response of the two magnon modes to top and bottom gates, their doping response, as well as the observed thickness dependence. In particular, the gate-tunability of magnons in CrSBr drops from up to 4 GHz in a bilayer to  ≈ 0.5 GHz in a 5-layer device, and vanishes for 8 layers.

The accuracy of the layer-resolved macrospin model is limited by several simplifications, mainly concerning the estimation of carrier density and electric field from PL measurements and the focus on the dominating linear dependencies of the magnetic sample parameters. In the future, we believe that experiments using a variable external magnetic field on CrSBr and other material systems, as well as studying higher-momentum magnons, can further improve our understanding of gate-dependent magnonic phenomena and expand our minimal model. Additionally, the magnon tunability could be increased by new gating techniques which surpass electrostatic gating in terms of the achievable doping and field^51,62,63^. Our findings open up new possibilities for using the on-chip control of magnons for magnonic circuits, e.g., for a phase shifter used in logic gates^39,64^.

## Methods

### Sample fabrication and crystal growth

The crystals of CrSBr were synthesized by chemical vapor transport and subsequently characterized by X-ray diffraction, crystal diffraction (powder and single crystal), transmission electron microscopy, energy dispersive X-ray analysis, Raman and IR spectroscopy, superconducting quantum interference device magnetometry, and magneto-transport measurements, as reported in ref. ^65^.

The samples were fabricated using a dry-transfer technique after exfoliation on PDMS. The contacts were patterned by electron beam lithography followed by evaporation of 3 nm Cr and 80 nm Au.

### Tr-reflectivity measurements

The tr-reflectivity measurements on the 3-, 5-, and 8-layer devices have been performed in a single-color pump and probe scheme. The laser source is a wavelength-tunable Ti:sapphire laser (Coherent Chameleon Ultra II, *τ*_pulse_ ≈ 140 ps, *f*_rep_ = 80 MHz). We spatially separate pump and probe beams before entering a reflective objective (Thorlabs LMM40x-P01) and focusing on the sample, into spot sizes of *d*_probe_ ≈ 1 μm, *d*_pump_ ≈ 3 μm. Thanks to the spatial separation of the reflected beams, we then filter out the pump beam using an iris. On the bilayer devices, we employed a two-color pump and probe scheme. For this, we used a high pump energy of 1.9 eV and probed around 1.67 eV, which is a higher-energy excitonic resonance of CrSBr^48^. We then filtered out the pump light using a lowpass filter in the detection path. In both configurations, the probe light is measured with a home-built photodetector (Hamamatsu photodiodes) using a lock-in amplifier synced to a chopper in the pump beam (*f* = 2.1 kHz). More details are provided in Supplementary Note 3.

### PL measurements

All PL measurements were conducted using a continuous wave laser with an excitation wavelength of 670 nm and a laser power of 25 μW. The incoming laser light was linearly polarized along the $\widehat{b}$-axis of the CrSBr flake. A refractive objective (Olympus LMPlanFL N 50x/0.50) focused the laser to a spot size of ≈1 μm. The resulting copolarized PL was captured with the Kymera 193i Spectrograph.

### Dielectric constant of CrSBr

The dielectric constant of CrSBr used in the electrostatic model was calculated using Hubbard-corrected DFT energy functionals (LDA+U). The complete dielectric function is shown in Supplementary Fig. 5.

## Supplementary information

Supplementary Information

Transparent Peer Review file

## Data Availability

The source data for this manuscript and its supplementary information is available in the Zenodo database under accession code 10.5281/zenodo.18982552.
