# Supplementary material for: Tunable magnons in a dual-gated 2D antiferromagnet
Source: Nat Commun. 2026 Jun 12;17:5262. doi: 10.1038/s41467-026-74067-z (PMC13263320; doi:10.1038/s41467-026-74067-z)
Supplement: Supplementary file 1 — Supplementary Information [file 41467_2026_74067_MOESM1_ESM.pdf]

Supplementary Information: Tunable magnons in a dual-gated 2D antiferromagnet

Nele Stetzuhn[1,2], Abhijeet M. Kumar[1], Sviatoslav Kovalchuk[1], Denis Yagodkin[1], Louis Simon[1], Samuel Mañas-Valero[3,4], Eugenio Coronado[3], Takashi Taniguchi[6], Kenji Watanabe[6], Deepika Gill[2], Sangeeta Sharma[1,2], Piet W. Brouwer[1], Clemens von Korff Schmising[2,5], Stefan Eisebitt[2,5], Kirill I. Bolotin[1]

[1] Department of Physics, Freie Universität Berlin, Arnimallee 14, 14195 Berlin, Germany

[2] Max Born Institute for Nonlinar Optics and Short-Pulse Spectroscopy, Max-Born-Str. 2A, 12489 Berlin, Germany

[3] Instituto de Ciencia Molecular, Universidad de Valencia, Dr. Moliner 50, Burjasot, 46100, Spain

[4] Department of Quantum Nanoscience, Kavli Institute of Nanoscience, Delft University of Technology, Delft 2628CJ, the Netherlands

[5] Institut für Optik und Atomare Physik, Technische Universität Berlin, Straße des 17. Juni 135, 10623 Berlin, Germany

[6] National Institute for Materials Science, Namiki 1-1, Tsukuba, 305-0044, Ibaraki, Japan

Contents

Supplementary Note 1 Thickness measurement of CrSBr flakes **3**

Supplementary Note 2 Temperature- and fluence-dependent magnon frequency shifts **4**

Supplementary Note 3 Tr-reflectivity measurements **5**

Supplementary Note 4 Gate-dependent PL and electrostatic modeling **7**

Supplementary Note 4.1 Gate-dependent PL and electrostatic model of the dual-gated trilayer device . . . 7

Supplementary Note 4.2 Gate-dependent PL and electrostatic model of the dual-gated 2-layer device 10

Supplementary Note 4.3 Gate-dependent PL and electrostatic model of the singlegated 2-layer device . . . 10

Supplementary Note 4.4 Gate-dependent PL and electrostatic model of 5-layer device 10

Supplementary Note 4.5 Gate-dependent PL and electrostatic model of 8-layer device 12

Supplementary Note 5 Bulk macrospin model **14**

Supplementary Note 6 Layer-resolved macrospin model **16**

Supplementary Note 7 Excitation of coherent magnon modes by laser pulses **18**

Supplementary Note 8 Gate-dependent magnetic phenomena **21**

Supplementary Note 9 Macrospin fit results **22**

Supplementary Note 10 Gate dependence of magnons in bilayer devices **25**

Supplementary Note 1 Thickness measurement of CrSBr flakes

To find the layer number of the measured flakes, we performed AFM measurements (Supplementary Fig. 1). We find the following thicknesses – Sample 1: 2.4 nm, Sample 2: 4 nm, Sample 1: 6.4 nm – corresponding to 3, 5 and 8 layers, respectively [1].

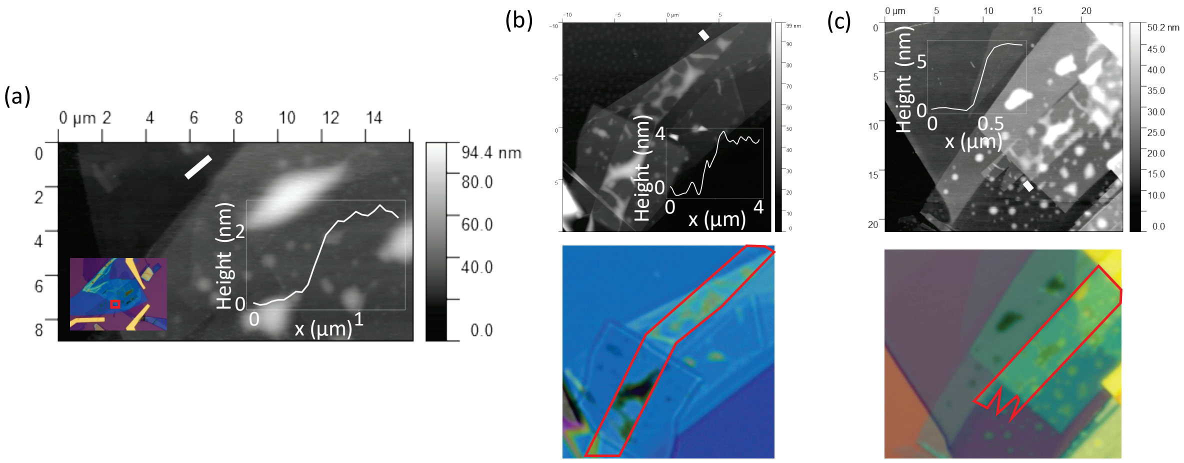

Supplementary Fig. 1: **AFM images.** (a) AFM image of a small portion of the trilayer device (marked in optical image). The height profile corresponds to a linecut of the CrSBr flake along the marker in the image. (b) AFM (top) and corresponding optical (bottom, CrSBr flake outlined) image of the 5-layer device before contact patterning. The height profile corresponds to a linecut of the CrSBr flake along the marker in the image. (c) AFM (top) and corresponding optical (bottom, CrSBr flake outlined) image of the 8-layer device before contact patterning. The height profile corresponds to a linecut of the CrSBr flake along the marker in the image.

Supplementary Note 2   Temperature- and fluence-dependent magnon frequency shifts

To exclude laser- or gate-related heating effects as the origin of magnon frequency changes, fluence- and temperature dependent measurements were conducted on the trilayer sample. We see in Supplementary Fig. 2a that a higher fluence leads to a downshift in magnon frequencies of $\Delta f_{\mathrm{IP}} \approx -(0.1 - 0.2\,\mathrm{GHz})$ and $\Delta f_{\mathrm{OP}} \approx -(0.4 - 0.8\,\mathrm{GHz})$. This is consistent with absorption-induced heating of the sample, as the magnon frequencies also shift down with temperature (Supplementary Fig. 2b). As shown in the main text, gating leads to an upshift of both magnon modes, so that we exclude heating as the underlying mechanism.

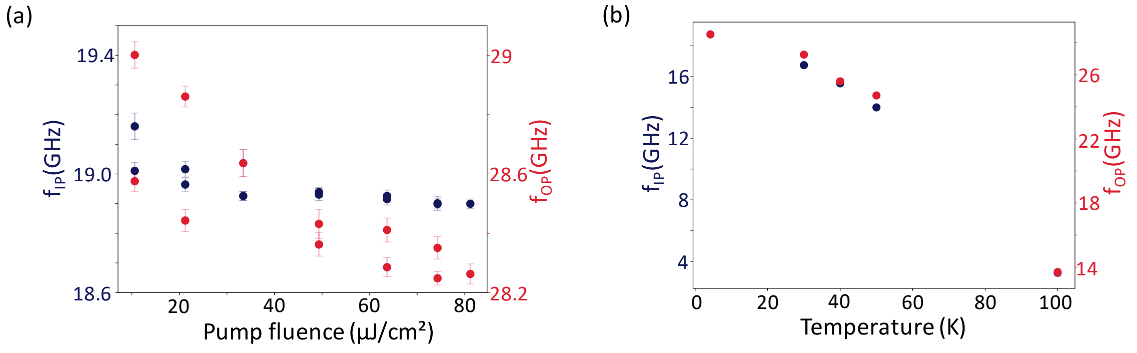

Supplementary Fig. 2: **Fluence- and temperature dependence of magnon modes.** Shift of f_{IP} and f_{OP} as a function of (a) pump laser fluence and (b) sample temperature in the trilayer sample.

Supplementary Note 3 Tr-reflectivity measurements

The single-color tr-reflectivity detection scheme is shown in Supplementary Fig. 3(a). To achieve a high signal-to-noise ratio in our experiments, we tune our pump and probe energies just below the X_B exciton resonance around 1.375 eV for low gate voltages (dark blue curve in Supplementary Fig. 3b). For higher gate voltages, it becomes neccessary to tune pump and probe to the trion resonance at 1.35 eV, as the exciton fades (green curve in Supplementary Fig. 3(b)). We see in Supplementary Fig. 3(c), that for intermediate gate voltages the signal at both resonances (orange: exciton, purple: trion) becomes weak, however, they allow us to observe oscillations at both resonances and we find the same frequency for f_{OP} (f_{IP} ambiguous at trion resonance).

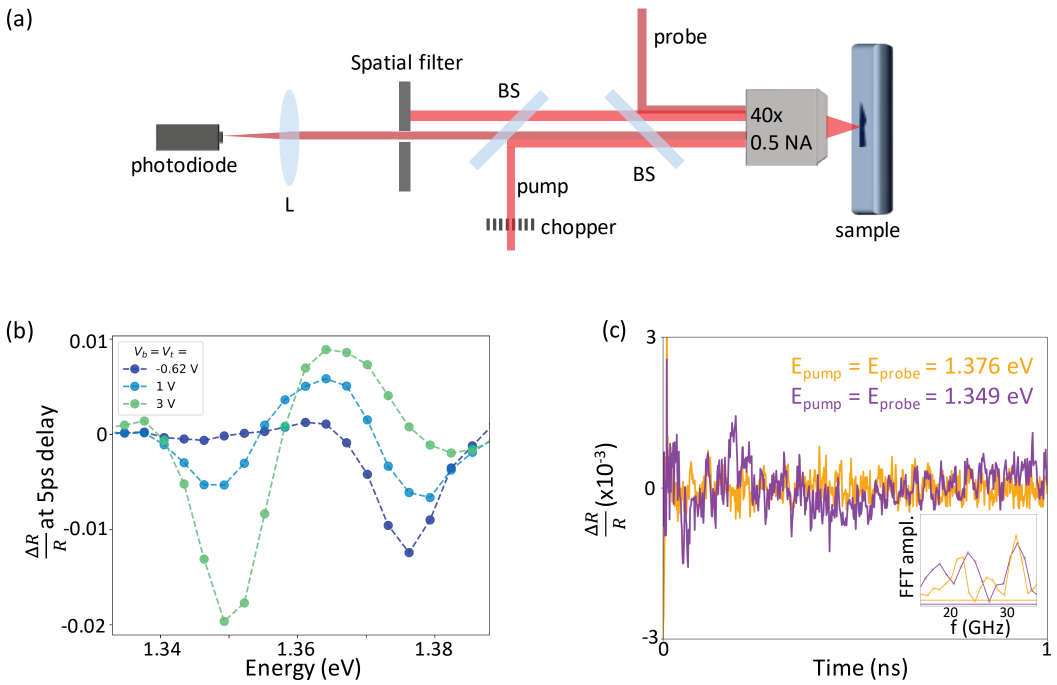

Supplementary Fig. 3: **Tr-reflectivity measurements.** (a) Detection scheme of single-color tr-reflectivity. (b) Tr-reflectivity spectra at small delay for different doping levels. The exciton resonance becomes weaker while the trion resonance strengthens for positive gate voltages. (c) Tr-reflectivity traces and extracted magnon frequencies (inset) measured at the exciton (orange) and trion (purple) resonances for $V_b = V_t = 3$ V after subtracting an exponential background.

In the two-color tr-reflectivity measurements, used for the bilayer samples, the probe is tuned to the higher excitonic resonance around 1.67 eV and the pump to 1.9 eV. We find that at this excitonic resonance, the possible window for the probe wavelength is quite large due to the large width of the resonance in the reflectivity spectrum. The spatial filter in Supplementary Fig. 3(a) is replaced with a low pass filter instead and the objective is changed to the Olympus LMPlanFL N 50x/0.50. The two-color detection scheme offers a better filtering of the pump light background, which is important especially for lower signal-to-noise ratio measurements on the bilayer samples.

Additionally, we mount a permanent magnet in proximity to the sample to increase signal strength. To estimate the strength of the external magnetic field, we use the open source software FEMM4.2 [2] (Supplementary Fig. 4). At the sample position, the field is around 100 mT. It should be noted that the exact position of the sample can differ

slightly between the measurements of the three devices, resulting in differences of the external magnetic field. Also, the external field has in- and out-of-plane components, as evidenced by the observation of both f_{IP} and f_{OP} modes. We neglect this fact in the macrospin model.

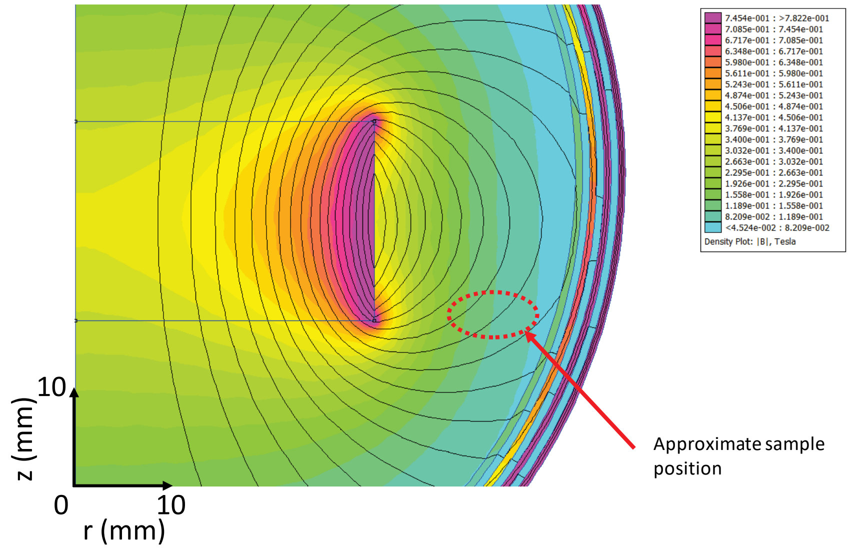

Supplementary Fig. 4: **Simulated field of permanent magnet used for experiments in the manuscript.** Simulated magnetic field of the permanent magnet (Neodym 38, r = 3 mm) as a function of height z and radial distance r.

Supplementary Note 4 Gate-dependent PL and electrostatic modeling

To quantify the layer-resolved electron densities and electric fields, we follow the capacitor model suggested in [3, 4]. In this model, the CrSBr layers correspond to capacitor plates separated by a dielectric with $\epsilon_{\mathrm{CrSBr}}$ and spaced by interlayer distance $d_{\mathrm{CrSBr}} = 0.8\,\mathrm{nm}$. Using density functional theory (DFT), we calculate the out-of-plane $\epsilon_{\mathrm{CrSBr}}(E)$ for mono-, trilayer and bulk, shown in Supplementary Fig. 5. As DC dielectric constants (real part of ϵ at $E = 0$), we find $\epsilon_{\mathrm{CrSBr}} = 3.8$ for a monolayer, $\epsilon_{\mathrm{CrSBr}} = 5.19$ for the trilayer and $\epsilon_{\mathrm{CrSBr}} = 7.42$ for the bulk. The graphite gates are modelled as capacitor plates with a dielectric of $\epsilon_{\mathrm{hBN}} = 3.76$ [5] and a thickness of the hBN flakes d_{hBN}.

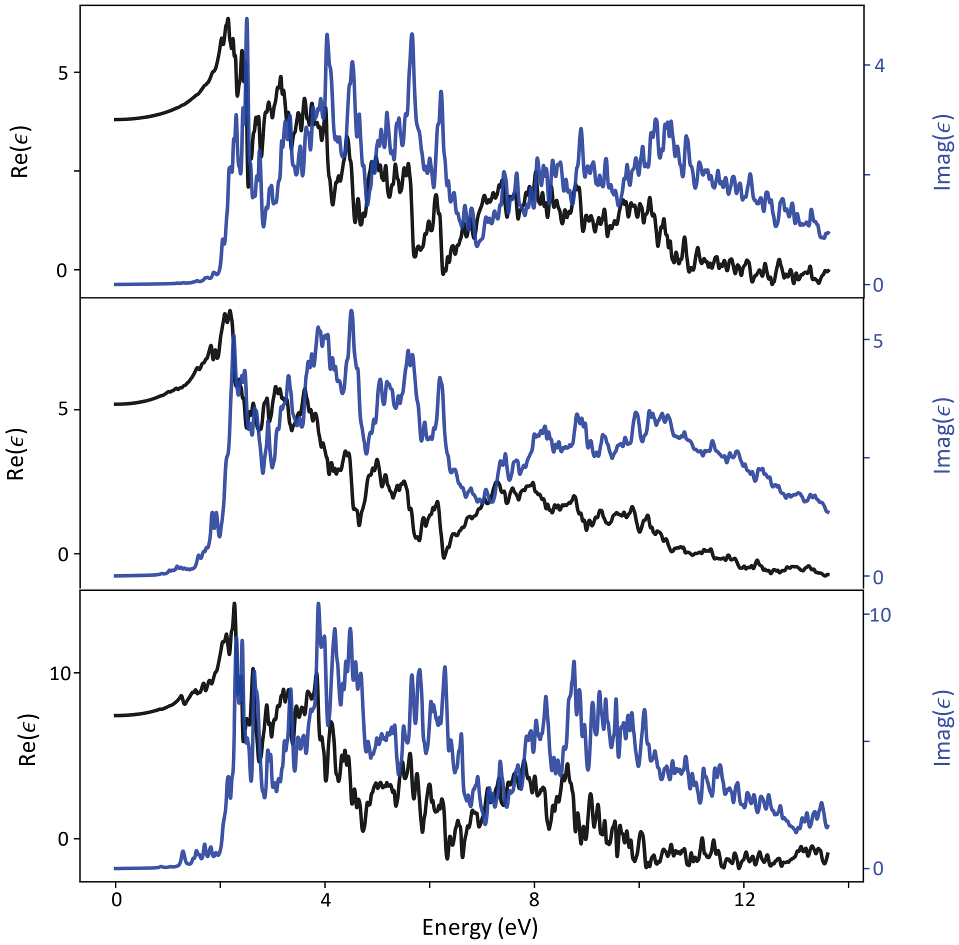

Supplementary Fig. 5: **Dielectric function of CrSBr.** Calculated real and imaginary parts of the out-of-plane dielectric function of (a) monolayer, (b) trilayer and (c) bulk CrSBr.

Supplementary Note 4.1 Gate-dependent PL and electrostatic model of the dual-gated trilayer device

The equivalent circuit for the capacitor model for the trilayer is shown in Supplementary Fig. 6a, and the resulting energy diagram in Supplementary Fig. 6b. In the trilayer sample, the hBN thicknesses on both sides are $d_{\mathrm{hBN}} = 10\,\mathrm{nm}$. The system of equations to extract the Fermi level with respect to the bottom of the conduction band $E_{\mathrm{F},i}$ (in eV, see

Supplementary Fig. 6b) and the carrier density n_i (where i is top, middle and bottom) in the trilayer is as follows:

$$0 = (V_t + V_{t,0}) - E_{F,t} - \frac{en_t}{C_t} - \frac{C_{CrSBr}}{C_t}(E_{F,t} - E_{F,m}) \tag{1}$$

$$0 = (V_b + V_{b,0}) - E_{F,b} - \frac{en_b}{C_t} - \frac{C_{CrSBr}}{C_b}(E_{F,b} - E_{F,m}) \tag{2}$$

$$0 = -en_m - \frac{C_{CrSBr}}{e}(E_{F,m} - E_{F,t}) - \frac{C_{CrSBr}}{e}(E_{F,m} - E_{F,b}). \tag{3}$$

Here $C_{CrSBr} = \frac{\epsilon_0 \epsilon_{CrSBr}}{d_{CrSBr}}$ and $C_{t/b} = \frac{\epsilon_0 \epsilon_{hBN}}{d_{hBN,t/b}}$. To calculate the electron densities, we assume a two-dimensional density of states (DOS) similar to that in TMDs [4]. We note that behavior suggesting a one-dimensional DOS has been reported in CrSBr [6], which would lead to a stronger increase in n for energies just above the conduction band due to the emergence of van't Hove singularities. However, the overall dependence of n and F on the gate voltages, and therefore the main results of the manuscript, will not change with a different DOS.

In the PL data of Fig. 2 in the main manuscript we observe two key features which are of importance for the electrostatic modeling: Our CrSBr crystals are intrinsically n-doped (signified by the dominance of trions over excitons at zero gate voltage) and our samples have a built-in electric field (as shown by the field dependence of the middle layer exciton-to-trion ratio). To model the intrinsic n-doping, we introduce positive offset voltages $V_{t,0}$ and $V_{b,0}$ in Eqs. (1) and (2) – corresponding to a downshift of the conduction bands of all layers with respect to the vacuum level. To model the built-in electric field, we use $V_{t,0} \neq V_{b,0}$ to introduce an offset between the conduction bands across layers.

To find reasonable values for $V_{t,0}$ and $V_{b,0}$, we use the exciton-to-trion ratios $I_B = \frac{I_{X_B}}{I_{X_B^-}}$ and $I_{B'} = \frac{I_{X'_B}}{I_{X'^-_B}}$ as indicators for the carrier densities of the outer layers, $n_t + n_b$, and the middle layer, n_m. We treat the excitons and trions as intralayer, since our applied external magnetic field of ~ 0.1 T only leads to a small canting angle of the macrospins (around 3 degree, comparing to Fig. 1(d) in [7]). The excitons will therefore only have a small interlayer contribution. We fit the gate-dependent PL spectra using

- two Gaussian peaks to describe the excitons X_B and X'_B around 1.37 – 1.375 eV,
- two Gaussian peaks to describe the trions X_B and X'_B around 1.34 – 1.35 eV,
- a weakly gate-dependent background of four Gaussians between 1.32 – 1.36 eV to describe the background visible in this energy range. This is the minimum amount of peaks necessary to adequately fit the spectra without significant trion contributions. We keep the number of background peaks constant for all spectra.

As it is difficult to directly correlate I_B and $I_{B'}$ to the electron densities by fitting (especially as we cannot resolve n_t and n_b separately in the PL), we manually vary the offset voltages in the model to match the onset of doping to drops in the exciton-to-trion ratios.

From the symmetric behavior of I_B in Supplementary Fig. 6c we deduce the following:

- We see the highest exciton-to-trion ratio I_B when both V_b and $V_t < -0.8$ V. This means that increasing either gate voltage above this value should result in at least one of the layers being electron doped.
- When varying only one of the gate voltages while fixing the other to a sufficiently negative value, either the top or bottom layer stays almost undoped, as I_B stays nearly constant after an initial drop.
- When both V_b and $V_t > 0$ V, both top and bottom layers should be electron-doped, as $I_B \approx 0$ for those ranges of the gating diagram.

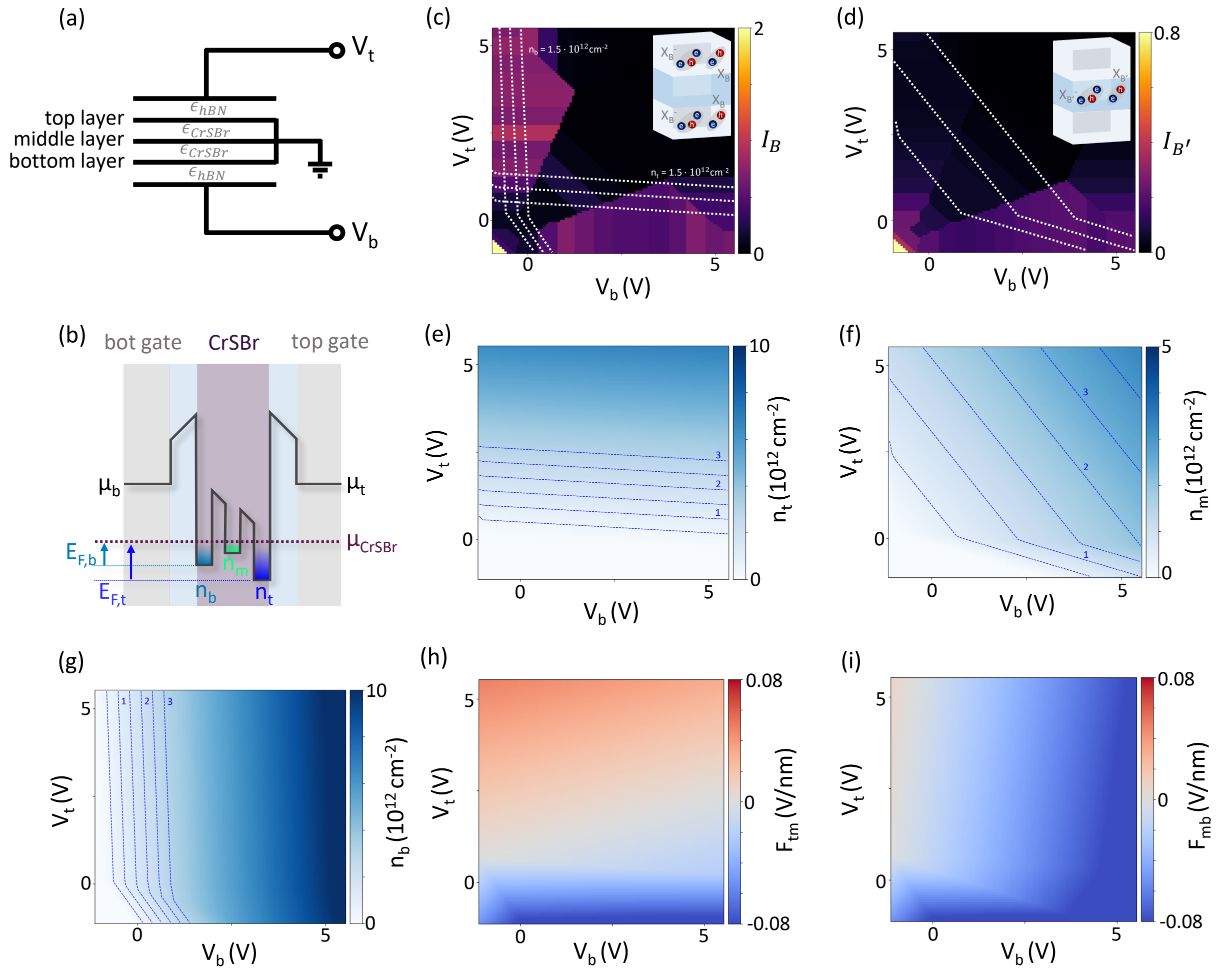

Supplementary Fig. 6: **Photoluminescence and electrostatic model of trilayer CrSBr.** (a) Scheme of the capacitor model for a trilayer CrSBr with top and bottom gate and hBN dielectric. (b) Band alignment sketch when the sample is doped. The Fermi energies $E_{\mathrm{F},i}$ are defined as the difference between the conduction band and the chemical potential μ_{CrSBr}. The top and bottom gate voltages control μ_t and μ_b. (c) Exciton-to-trion ratio I_B for excitons and trions localized to top and bottom layer (see inset). The white dashed lines are guides to the eye corresponding to the contour lines for n_t and n_m being 0.5, 1 and $1.5 \times 10^{12}\,\mathrm{cm}^{-2}$ shown in (e), (g). (d) Exciton-to-trion ratio $I_{\mathrm{B}'}$ for excitons and trions localized in the middle layer (see inset). The white dashed lines are guides to the eye corresponding to the contour lines for n_m = 0.5, 1 and $1.5 \times 10^{12}\,\mathrm{cm}^{-2}$ shown in (f). (e-g) Electron density in the top (e), middle (f) and bottom (g) layer. The contour lines show densities of 0.5, 1, 1.5, 2, 2.5 and $3 \times 10^{12}\,\mathrm{cm}^{-2}$. (h-i) Electric field between top and middle (h) and middle and bottom (i) layers.

This gives us an upper limit for $V_{\mathrm{t},0}$ and $V_{\mathrm{b},0}$ of $\sim$ 2.5 V, as increasing the offset voltages beyond this limit would mean that the sample is doped in the top or bottom layer for all gate voltages we reach in the experiment.

In the gate-dependence of $I_{\mathrm{B}'}$ in Supplementary Fig. 6d, we see that

- $I_{\mathrm{B}'}$ drops faster when applying a top gate voltage. From this, we infer that $V_{\mathrm{t},0} < V_{\mathrm{b},0}$, because a smaller V_t is then sufficient to dope the middle layer.

We find that $V_{t,0} = 0.6\,\mathrm{V}$ and $V_{b,0} = 1.7\,\mathrm{V}$ fulfill all the requirements mentioned above and adequately reproduce the behavior seen in PL. The resulting layer-resolved doping maps are shown in Supplementary Fig. 6(e-g), with contour lines for selected doping levels ($0.5 - 3 \times 10^{12}\,\mathrm{cm}^{-2}$).

We show the same contour lines as guides to the eye in the experimental I_B and $I_{B'}$ maps in Supplementary Fig. 6c, d. In Supplementary Fig. 6c, we see that the contour lines show the same symmetry with gates as I_B, and we see a drop in I_B for a similar n_t or n_b (around $\sim 1.5 \times 10^{12}\,\mathrm{cm}^{-2}$). In Supplementary Fig. 6d, the asymmetry of the contour line for $n_m = 0.5 \times 10^{12}\,\mathrm{cm}^{-2}$ shows that we need a higher V_b to dope the middle layer when $V_t < 0$ than in the opposite case. The modeled n_m also shows a quick increase when increasing V_t for large enough V_b, concurrent with a drop in $I_{B'}$ in the same case. While the onset of the n_m happens for slightly lower gate voltages than the drop in $I_{B'}$, the offset voltages of $V_{t,0} = 0.6\,\mathrm{V}$ and $V_{b,0} = 1.7\,\mathrm{V}$ give the best overall correlation between modeling and PL data. The gate-dependent electric fields between the layers are shown in Supplementary Fig. 6h, i.

It should be noted that changing the offset voltages slightly does not change the overall predicted behavior of the frequencies in the macrospin model (i.e. the gate sensitivity or frequency range). It can, however, slightly change the onset of frequency shifts as well as the fitted ν_a, ν_E and η_M.

Supplementary Note 4.2 Gate-dependent PL and electrostatic model of the dual-gated 2-layer device

In the doping-dependent PL of the dual-gated bilayer device (Supplementary Fig. 7(a)), the X_B exciton at 1.367 eV disappears around $V_b = V_t = 3 - 4\,\mathrm{V}$ (due to the thicker top (26 nm) and bottom (19 nm) hBN). In the same voltage range, the trion at 1.342 eV brightens. We fix $V_{b,0} = V_{t,0} = 0\,\mathrm{V}$ in the model to match this behavior.

In the dual-gated bilayer sample, the system of equations for the capacitor model (Supplementary Fig. 7(b)) reduces to

$$0 = (V_t + V_{t,0}) - E_{F,t} - \frac{en_t}{C_t} - \frac{C_{CrSBr}}{C_t}(E_{F,t} - E_{F,b}) \tag{4}$$

$$0 = (V_b + V_{b,0}) - E_{F,b} - \frac{en_b}{C_t} - \frac{C_{CrSBr}}{C_b}(E_{F,b} - E_{F,t}). \tag{5}$$

The resulting carrier densities and the electric field are shown in Supplementary Fig. 7(c-e).

Supplementary Note 4.3 Gate-dependent PL and electrostatic model of the singlegated 2-layer device

In the second bilayer device, only the bottom gate (hBN thickness of 24 nm) was functional. In the top half on Supplementary Fig. 7(f) we show the gate-dependent exciton-to-trion ratio for this sample. We use the capacitive model for a single-gated device:

$$0 = -en_t - C_{CrSBr}(E_{F,t} - E_{F,b}) \tag{6}$$

$$0 = (V_b + V_{b,0}) - E_{F,b} - \frac{en_b}{C_t} - \frac{C_{CrSBr}}{C_b}(E_{F,b} - E_{F,t}) \tag{7}$$

and fix $V_{b,0} = 1\,\mathrm{V}$ to match the doping onset to the observed drop in the exciton-to-trion ratio in the bottom of Supplementary Fig. 7(f).

Supplementary Note 4.4 Gate-dependent PL and electrostatic model of 5-layer device

In the gate-dependent PL of the 5-layer device we observed that the top gate did not function properly. Therefore, we only discuss the dependence on the bottom gate voltage. The V_b-dependent photoluminescence of the 5-layer device in Supplementary Fig. 8(a) again shows two split excitonic peaks at $X_B \approx 1.376\,\mathrm{eV}$ and $X'_B \approx 1.370\,\mathrm{eV}$, which we hypothesize to reside in the outer and inner layers, respectively. The intensities of the excitonic peaks for the undoped sample ($V_b < 0\,\mathrm{V}$) are now comparable, which we attribute to the increased number of layers hosting X'_B excitons. For $V_b > 0\,\mathrm{V}$, the intensity of the X_B exciton drops, and the trion resonance X_{B^-} around 1.345 eV brightens. Meanwhile,

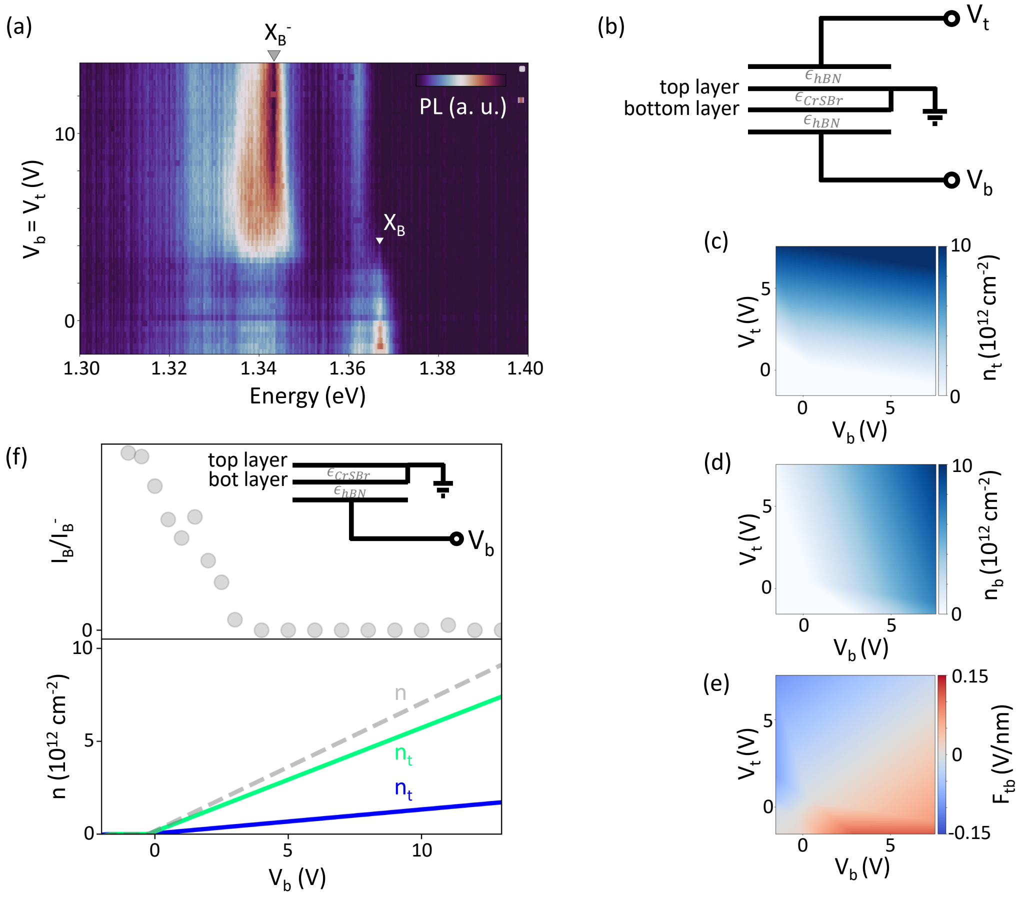

Supplementary Fig. 7: **Photoluminescence and electrostatic model of 2-layer CrSBr.** (a) $V_\mathrm{b} = V_\mathrm{t}$-dependent photoluminescence map of the dual-gated bilayer CrSBr device. (b) Scheme of the capacitor model for the dual-gated bilayer CrSBr with top and bottom gate and hBN dielectric. (c,d) Electron density in the top and bottom layers of the dual-gated bilayer CrSBr. (e) Electric field between the top and bottom layers of the dual-gated bilayer CrSBr. (f) Exciton-to-trion ratio in the singlegated bilayer CrSBr as a function of bottom gate voltage (top), together with the modelled carrier densities (bottom). The inset shows the schematic of the capacitor model.

the X'_B exciton remains visible for all measured gate voltages.

When modeling the 5-layer device as shown in Supplementary Fig. 8b, we expand the system of equations to

$$0 = -en_1 - C_\mathrm{CrSBr}(E_\mathrm{F,1} - E_\mathrm{F,2}) \tag{8}$$

$$0 = (V_\mathrm{b} + V_\mathrm{b,0}) - E_\mathrm{F,5} - \frac{en_5}{C_\mathrm{b}} - \frac{C_\mathrm{CrSBr}}{C_\mathrm{b}}(E_\mathrm{F,5} - E_\mathrm{F,4}) \tag{9}$$

$$0 = -en_2 - \frac{C_\mathrm{CrSBr}}{e}(E_\mathrm{F,2} - E_\mathrm{F,1}) - \frac{C_\mathrm{CrSBr}}{e}(E_\mathrm{F,2} - E_\mathrm{F,3}) \tag{10}$$

$$0 = -en_3 - \frac{C_\mathrm{CrSBr}}{e}(E_\mathrm{F,3} - E_\mathrm{F,2}) - \frac{C_\mathrm{CrSBr}}{e}(E_\mathrm{F,3} - E_\mathrm{F,4}) \tag{11}$$

$$0 = -en_4 - \frac{C_\mathrm{CrSBr}}{e}(E_\mathrm{F,4} - E_\mathrm{F,3}) - \frac{C_\mathrm{CrSBr}}{e}(E_\mathrm{F,4} - E_\mathrm{F,5}) \tag{12}$$

The equation of the top layer does not include a top gate (Supplementary Fig. 8(b)). The hBN thicknesses in the 5-layer device are $d_\mathrm{hBN,top} = 5\,\mathrm{nm}$ and $d_\mathrm{hBN,bot} = 11\,\mathrm{nm}$. We use the trilayer $\epsilon_\mathrm{CrSBr} = 5.19$ calculated with DFT (Supplementary Fig. 5(b)). We assume an offset voltage $V_\mathrm{b,0} = 0.3\,\mathrm{V}$ to describe the trends in the gate-dependent PL in Supplementary Fig. 8(a). This leads to n_5 increasing around $V_\mathrm{b} \approx 1\,\mathrm{V}$. We also see that the charge carrier densities in the other layers remain below $\approx 2 \times 10^{12}\,\mathrm{cm}^{-2}$ for all applied gate voltages (Supplementary Fig. 8(i)), which agrees

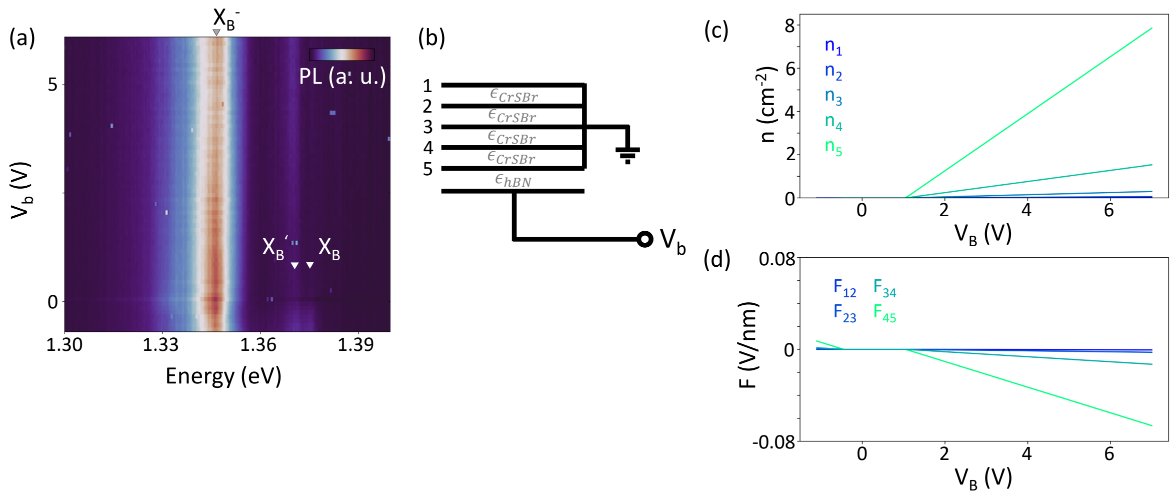

Supplementary Fig. 8: **Photoluminescence and electrostatic model of 5-layer CrSBr.** (a) V_b-dependent photoluminescence map of the 5-layer CrSBr device. (b) Scheme of the capacitor model for a 5-layer CrSBr with a bottom gate and hBN dielectric. (c) V_b-dependent electron density in the respective layers. (d) V_b-dependent electric field between the respective layers.

with the visibility of X'_B for all gate voltages. All the calculated fields and electron densities as a function of bottom gate for the 5-layer device are shown in Supplementary Fig. 8(c,d).

Supplementary Note 4.5 Gate-dependent PL and electrostatic model of 8-layer device

In the doping-dependent photoluminescence of the 8-layer device in Supplementary Fig. 9(a), we once again see two split excitonic peaks at $X_B \approx 1.372\,\mathrm{eV}$ and $X'_B \approx 1.366\,\mathrm{eV}$. In contrast to the tri- and 5-layer device, the X'_B peak is more intense than X_B, the latter only visible as a high-energy shoulder. This is consistent with X'_B emission stemming from excitons localized to the inner layers, as the number of such inner layers has increased from the 3- to 8-layer device.

We see X_B disappears for $V_b = V_t > -0.3\,\mathrm{V}$, simultaneously with a brightening of the trion resonance around 1.345 eV. The X'_B exciton remains visible for all measured gate voltages, similarly to the 5-layer device.

We expand the electrostatic model to 8 layers (Supplementary Fig. 9(b)) in the same manner as for 5 layers. We measure the hBN thicknesses to be $d_{\mathrm{hBN,top}} = 10\,\mathrm{nm}$ and $d_{\mathrm{hBN,bot}} = 6\,\mathrm{nm}$ with AFM. Due to the increased thickness of the device, we use the bulk dielectric constant $\epsilon_{\mathrm{CrSBr}} = 7.42$ calculated with DFT (Supplementary Fig. 5(c)). We assume the offset voltages $V_{t,0} = V_{b,0} = 1\,\mathrm{V}$. The resulting increase of electron density in the bottom layer n_8 around $V_b = V_t \approx -0.4\,\mathrm{V}$ (Supplementary Fig. 9q), followed by the top layer n_1 around $V_b = V_t \approx -0.2\,\mathrm{V}$ correlates well with the drop in the exciton intensity we observe at $V_b = V_t > -0.3\,\mathrm{V}$. The middle layers of the 8-layer device remain almost undoped (Supplementary Fig. 9(m, n)). All the calculated fields and electron densities for the 8-layer device are shown in Supplementary Fig. 9(c-q).

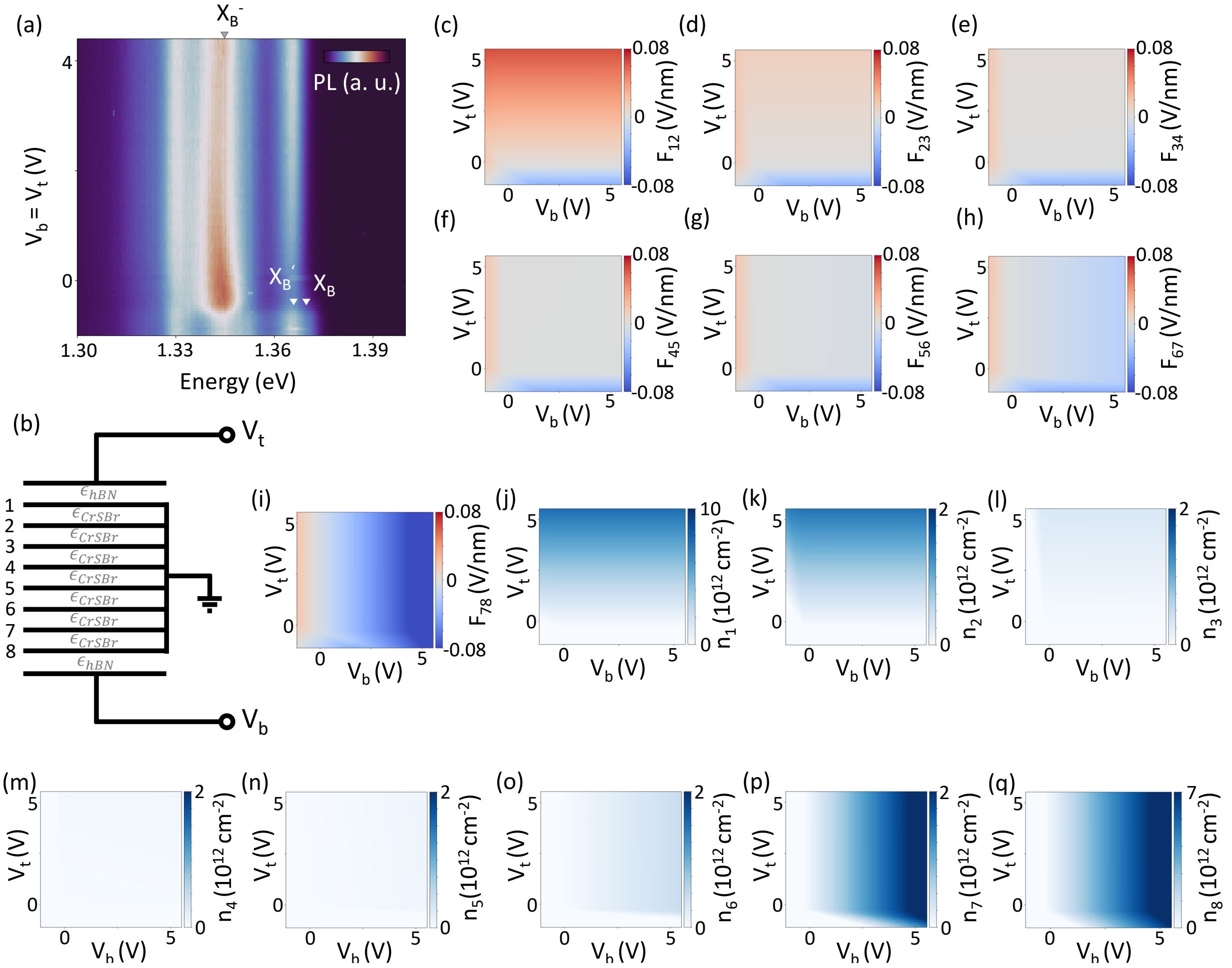

Supplementary Fig. 9: **Photoluminescence and electrostatic model of 8-layer CrSBr.** (a) Gate-dependent photoluminescence map of the 8-layer CrSBr device. (b) Scheme of the capacitor model for an 8-layer CrSBr with top and bottom gate and hBN dielectric. (c-h) Electric field between the respective layers (numbering see (b)). (i-q) Electron density in the respective layers (numbering see (b)).

Supplementary Note 5 Bulk macrospin model

For bulk antiferromagnets, we assume two coupled macrospins which repeat itself in periodic boundary conditions. We can write the LL equation

$$\frac{d\vec{m}_{1,2}}{dt} = -\gamma \vec{m}_{1,2} \times \vec{H}_{\mathrm{eff}_{1,2}} \tag{13}$$

where $\vec{m}_{1,2}$ are the macrospins in neighboring layers. The effective field $\vec{H}_{\mathrm{eff}_{1,2}}$ can be obtained from the energy E of the system:

$$E = -H_0\hat{c}\cdot(\vec{m}_1 + \vec{m}_2) + H_{\mathrm{E}}\vec{m}_1\cdot\vec{m}_2 - \frac{1}{2}H_{\mathrm{a}}((m_1^a)^2 + (m_2^a)^2) - \frac{1}{2}H_{\mathrm{b}}((m_1^b)^2 + (m_2^b)^2) \tag{14}$$

$$\vec{H}_{\mathrm{eff}_{1,2}} = -\nabla_{\vec{m}_{1,2}}E = H_0\hat{c} - H_{\mathrm{E}}\vec{m}_{2,1} + H_{\mathrm{a}}m_{1,2}^a\hat{a} + H_{\mathrm{b}}m_{1,2}^b\hat{b}. \tag{15}$$

Here, $\hat{a}, \hat{b}, \hat{c}$ point along the corresponding crystallographic axes of CrSBr. H_{b} describes the easy axis magnetic anisotropy and H_{a} the intermediate one. H_{E} is the interlayer exchange interaction, which aligns the neighboring macrospins antiparallely. The external magnetic field $\vec{H}_0 = H_0\hat{c}$ is assumed out-of-plane and determines the initial tilt θ of the macrospins away from the easy $\hat{b}$-axis.

As H_0 does not saturate the spins along the $\hat{c}$-axis, the equilibrium macrospins can be written as $\vec{m}_{1,2_0} = M_0\,(0, \sin\theta, \pm\cos\theta)$, where M_0 is the length of the macrospin. By inserting this expression into Eq. (14), we find θ by minimizing the equilibrium energy, $\frac{\mathrm{d}E}{\mathrm{d}\theta} = 0$, leading to the condition

$$H_0 = M_0\sin\theta(H_{\mathrm{b}} + 2H_{\mathrm{E}}). \tag{16}$$

Next, we express the macrospins as $\vec{m}_{1,2} = \vec{m}_{1,2_0} + \delta\vec{m}_{1,2}$, where $\delta\vec{m}_{1,2} << \vec{m}_{1,2_0}$ are small deviations from equilibrium after excitation. By linearizing the LL equation, we arrive at

$$\frac{\mathrm{d}}{\mathrm{d}t}\delta\vec{m}_{1,2} = -\gamma\left(\delta\vec{m}_{1,2}\times\vec{H}^0_{\mathrm{eff}_{1,2}} + \vec{m}_{1,2_0}\times\delta\vec{H}_{eff_{1,2}}\right) \tag{17}$$

where $\vec{H}_{\mathrm{eff}_{1,2}} = \vec{H}^0_{\mathrm{eff}_{1,2}} + \delta\vec{H}_{\mathrm{eff}_{1,2}}$.

From the condition $\vec{m}_{1,2_0}\cdot\delta\vec{m}_{1,2} = 0$, we obtain $\delta\vec{m}_{1,2} = M_0\left(\delta m^{\mathrm{a}}_{1,2}, \mp\delta m^{||}_{1,2}\cos\theta, \delta m^{||}_{1,2}\sin\theta\right)$. Inserting this into Eq. (17) and using the ansatz $\delta\vec{m}_{1,2}(t) = \delta\vec{m}_{1,2}(t=0)e^{-i\omega t}$ results in

$$\frac{i\omega}{\gamma M_0^2}\begin{pmatrix}\delta m_1^a\\ \delta m_1^{||}\\ \delta m_2^a\\ \delta m_2^{||}\end{pmatrix} = \begin{pmatrix} 0 & -H_{\mathrm{E}} - H_{\mathrm{b}}\cos^2\theta & 0 & H_{\mathrm{E}}\cos(2\theta)\\ H_{\mathrm{E}} + H_{\mathrm{b}} - H_{\mathrm{a}} & 0 & H_{\mathrm{E}} & 0\\ 0 & H_{\mathrm{E}}\cos(2\theta) & 0 & -H_{\mathrm{E}} - H_{\mathrm{b}}\cos^2\theta\\ H_{\mathrm{E}} & 0 & H_{\mathrm{E}} + H_{\mathrm{b}} - H_{\mathrm{a}} & 0\end{pmatrix}\begin{pmatrix}\delta m_1^a\\ \delta m_1^{||}\\ \delta m_2^a\\ \delta m_2^{||}\end{pmatrix}. \tag{18}$$

Solving this equation gives an analytical solution for the eigenvalues:

$$\begin{aligned} f_{\mathrm{IP}} &= \pm\frac{\gamma M_0^2}{2\pi}\sqrt{(H_{\mathrm{b}} - H_{\mathrm{a}})(H_{\mathrm{b}} + 2H_{\mathrm{E}})\left(1 - \frac{H_0^2}{(2H_{\mathrm{E}} + H_{\mathrm{b}} - H_{\mathrm{a}})^2}\right)}\\ f_{\mathrm{OP}} &= \pm\frac{\gamma M_0^2}{2\pi}\sqrt{(H_{\mathrm{b}} - H_{\mathrm{a}} + 2H_{\mathrm{E}})(H_{\mathrm{b}} + 2H_{\mathrm{E}})\left(2H_{\mathrm{E}}\frac{H_0^2}{(2H_{\mathrm{E}} + H_{\mathrm{b}} - H_{\mathrm{a}})^2} - H_{\mathrm{b}}\left(\frac{H_0^2}{(2H_{\mathrm{E}} + H_{\mathrm{b}} - H_{\mathrm{a}})^2} - 1\right)\right)}. \end{aligned} \tag{19}$$

As the ansatz assumes that every second macrospin is exactly the same, these two solutions correspond to the case of zero momentum magnons (i.e. f_{IP} and f_{OP} in bulk experiments).

For magnons with $k_z \neq 0$, however, there is a phase difference between every second macrospin. Including this into the solutions of Eq. (17) gives an analytical solution for the dispersion along k_z. The phase difference changes the

interlayer exchange term in the effective field of each macrospin $\vec{m}_i$ (where i is the layer number), since the macrospins above and below are no longer exactly the same. The effective field in layer i becomes

$$\vec{H}_{\mathrm{eff}_i} = H_0\hat{c} - \frac{1}{2}H_\mathrm{E}(\vec{m}_{i-1} + \vec{m}_{i+1}) + H_\mathrm{a} m_i^a \hat{a} + H_\mathrm{b} m_i^b \hat{b}. \tag{20}$$

To solve the new LL equation, we assume the following: Before the excitation, the equilibrium macrospins in every second layer are the same, $\vec{m}_{i+1_0} = \vec{m}_{i-1_0}$. For the deviations after the excitations, we use a plane wave ansatz $\delta\vec{m}_j(t) = \delta\vec{m}_j(t=0)e^{-i\omega t}e^{ik_z j d_z}$, where d_z is the interlayer spacing in z-direction and j describes the layer number. Inserting the new exchange term from Eq. (20) in Eq. (17) gives rise to the terms

$$\sim H_\mathrm{E} M_0 \begin{pmatrix} \delta m_i^{||}(\cos^2\theta - \sin^2\theta) \\ \delta m_i^a \cos\theta \\ \delta m_i^\mathrm{a} \sin\theta \end{pmatrix} - H_\mathrm{E} M_0 \begin{pmatrix} \delta m_{i-1}^{||}(\sin^2\theta - \cos^2\theta) \\ \delta m_{i-1}^a \cos\theta \\ -\delta m_{i-1}^a \sin\theta \end{pmatrix} (1 + e^{-2ik_z d_z}) \tag{21}$$

which are the same as the ones found for $k_z = 0$, apart from the last term $\propto e^{-2ik_z d_z}$. This changes the matrix equation from Eq. (18) to:

$$\frac{i\omega}{\gamma M_0^2}\begin{pmatrix} \delta m_1^a \\ \delta m_1^{||} \\ \delta m_2^a \\ \delta m_2^{||} \end{pmatrix} = \begin{pmatrix} 0 & -H_\mathrm{E} - H_\mathrm{b}\cos^2\theta & 0 & H_\mathrm{E}\cos(2\theta)\left(1 + e^{-2ik_z\cdot d_z}\right) \\ H_\mathrm{E} + H_\mathrm{b} - H_\mathrm{a} & 0 & H_\mathrm{E}\left(1 + e^{-2ik_z\cdot d_z}\right) & 0 \\ 0 & H_\mathrm{E}\cos(2\theta)\left(1 + e^{-2ik_z\cdot d_z}\right) & 0 & -H_\mathrm{E} - H_\mathrm{b}\cos^2\theta \\ H_\mathrm{E}\left(1 + e^{-2ik_z\cdot d_z}\right) & 0 & H_\mathrm{E} + H_\mathrm{b} - H_\mathrm{a} & 0 \end{pmatrix} \begin{pmatrix} \delta m_1^a \\ \delta m_1^{||} \\ \delta m_2^a \\ \delta m_2^{||} \end{pmatrix}. \tag{22}$$

We solve this to extract the dispersion relation in Fig. 3(a) of the main text.

In Supplementary Fig. 10 we show the dependance of the modes on macrospin length, anisotropies and interlayer exchange. They mirror those shown in Fig. 3 of the main text for the numerical model. We also see that increasing H_a and decreasing H_b has a similar effect on the frequencies, and thus we keep H_b unaffected by n or F in the fits.

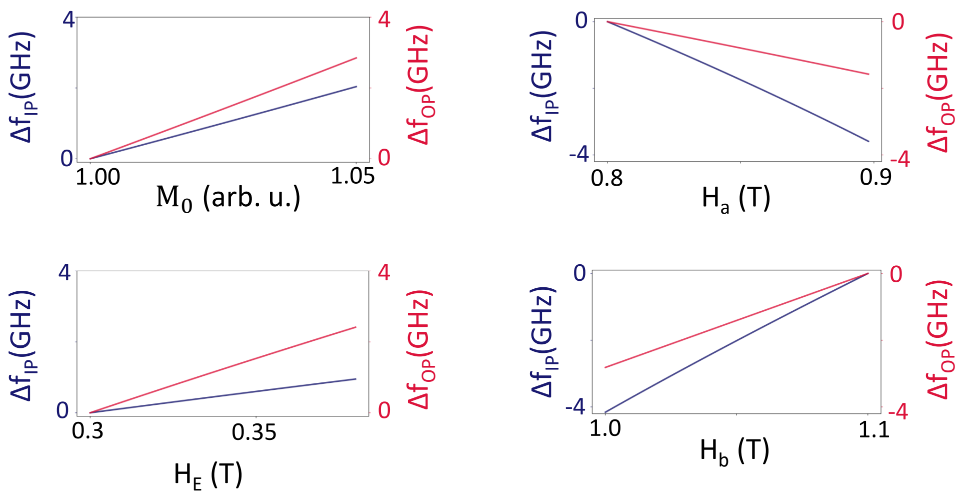

Supplementary Fig. 10: **Analytical macrospin model.** Influence of changing (a) M_0, (b) H_E, (c) H_a and (d) H_b on the magnon modes.

Supplementary Note 6 Layer-resolved macrospin model

For the layer-resolved macrospin model, we again introduce a layer-dependent effective field into the LL equation. Now, however, the parameters can be varied independently from each other in each layer. We start with the LL equation

$$\frac{d\vec{m}_i}{dt} = -\gamma\vec{m}_i \times \vec{H}_{\text{eff}_i}. \tag{23}$$

The energy of the system is given by

$$E = -\vec{H}_0 \sum_i \vec{m}_i + \sum_{\langle i,j\rangle} H_{\text{E}_{ij}} \vec{m}_i \cdot \vec{m}_j - \frac{1}{2}\sum_i H_{\text{a}_i} \left(m_i^{\text{a}}\right)^2 - \frac{1}{2} H_{\text{b}} \sum_i \left(m_i^{\text{b}}\right)^2. \tag{24}$$

We now use explicitly layer-dependent parameters, in particular the layered macrospin $\vec{m}_{i,0} = M_{0,i}\,(0, \sin\theta_i, \pm\cos\theta_i)$, described by their length $M_{0,i}$ and tilt angle θ_i away from the easy $\hat{b}$-axis. Additionally, we introduce the layer-resolved interlayer exchange interaction between layers i and j, $H_{\text{E}_{\text{i,j}}}$, as well as the intermediate axis anisotropy in individual layers i, H_{a_i}. The external field and easy-axis anisotropy remain layer-independent. The effective field in layer i then results to

$$\vec{H}_{\text{eff}_i} = -\nabla_{\vec{m}_i} E = \vec{H}_0 - \sum_{<i,j>} H_{E_{i,j}} \vec{m}_j + H_{\text{a}_i} m_i^{\text{a}} \hat{a} + H_{\text{b}} m_i^{\text{b}} \hat{b}. \tag{25}$$

By minimizing the energy in Eq. (24) with respect to the θ_i, we can find their equilibrium position, analogous to the bulk model in Eq. (16). For three layers, the explicit system of equations is

$$\frac{\mathrm{d}E}{\mathrm{d}\theta_1} = -H_0\cos\theta_1 + M_{0,2}H_{\text{E}_{1,2}}(\cos\theta_1\sin\theta_2 + \sin\theta_1\cos\theta_2) + M_{0,1}H_{\text{b}}\cos\theta_1\sin\theta_1 = 0 \tag{26}$$

$$\frac{\mathrm{d}E}{\mathrm{d}\theta_2} = -H_0\cos\theta_2 + M_{0,1}H_{\text{E}_{1,2}}(\cos\theta_2\sin\theta_1 + \sin\theta_2\cos\theta_1) + M_{0,3}H_{\text{E}_{2,3}}(\cos\theta_2\sin\theta_3 + \sin\theta_2\cos\theta_3) + M_{0,2}H_{\text{b}}\cos\theta_2\sin\theta_2 = 0 \tag{27}$$

$$\frac{\mathrm{d}E}{\mathrm{d}\theta_3} = -H_0\cos\theta_3 + M_{0,2}H_{\text{E}_{2,3}}(\cos\theta_3\sin\theta_2 + \sin\theta_3\cos\theta_2) + M_{0,3}H_{\text{b}}\cos\theta_3\sin\theta_3 = 0, \tag{28}$$

which we solve numerically.

Next, we again linearize the LL equation in Eq. (23) and write $\delta\vec{m}_i = M_{0,i}\left(\delta m_i^{\text{a}}, \mp\delta m_i^{||}\cos\theta_i, \delta m_i^{||}\sin\theta_i\right)$. Inserting this into Eq. (17) and using the ansatz $\delta\vec{m}_i(t) = \delta\vec{m}_i(t=0)e^{-i\omega t}$ results in the following matrix equation for the case of three layers:

$$\frac{i\omega}{\gamma}\begin{pmatrix}\delta m_1^a\\ \delta m_1^{||}\\ \delta m_2^a\\ \delta m_2^{||}\\ \delta m_3^a\\ \delta m_3^{||}\end{pmatrix} = \begin{pmatrix} 0 & -A_{1,2}-B_1 & 0 & C_{1,2} & 0 & 0\\ A_{1,2}+D_1 & 0 & E_{1,2} & 0 & 0 & 0\\ 0 & C_{1,2} & 0 & -A_{2,1}-A_{2,3}-B_2 & 0 & C_{2,3}\\ E_{1,2} & 0 & A_{2,1}+A_{2,3}+D_2 & 0 & E_{2,3} & 0\\ 0 & 0 & 0 & C_{2,3} & 0 & -A_{3,2}-B_3\\ 0 & 0 & E_{2,3} & A_{3,2}+D_3 & 0 & \end{pmatrix}\begin{pmatrix}\delta m_1^a\\ \delta m_1^{||}\\ \delta m_2^a\\ \delta m_2^{||}\\ \delta m_3^a\\ \delta m_3^{||}\end{pmatrix} \tag{29}$$

where

$$\begin{aligned} A_{i,j} &= M_{0,i}M_{0,j}H_{E_{i,j}}\frac{\cos\theta_j}{\cos\theta_i}\\ B_i &= M_{0,i}^2 H_{\text{b}}\cos^2\theta_i\\ C_{i,j} &= M_{0,i}M_{0,j}H_{E_{i,j}}\cos(\theta_i+\theta_j)\\ D_i &= M_{0,i}^2\left(H_{\text{b}} - H_{a_i}\right)\\ E_{i,j} &= M_{0,i}M_{0,j}H_{E_{i,j}}. \end{aligned}$$

Eq. (29) can be expanded for an arbitrary amount of layers N and can be solved numerically.

What do the "extra" frequencies in this model compared to the analytical one describe? As we no longer assume the macrospins of every second layer to be identical, we implicitly allow a phase difference between the macrospins across layers, i.e. a finite momentum k_z of the magnons. As a result, the additional modes in the numerical model describe the magnon dispersion along k_z (when using sufficiently high N). For large N, we extract the k_z value of each eigenmode via Fast Fourier transform and arrive at the dispersion relation for thick samples. The solutions of the numerical model of the two branches for $f_{IP}(k = 0)$ and $f_{OP}(k = 0)$ should therefore be the same as in the analytical model of Eq. (19) (excluding a factor 2 in interlayer exchange field). We show this agreement in Supplementary Fig. 11(a) (blue stars: analytical results from Eq. (19), black empty circles: numerical model for $N = 100$, red solid line: analytical dispersion from Eq. (21)). Using periodic boundary conditions in the numerical model yields identical results to the analytical dispersion (blue circles in Supplementary Fig. 11(a)). In that sense, the numerical and analytical model converge for thick samples. However, the numerical model allows us to include layer-resolved changes of the magnetic parameters.

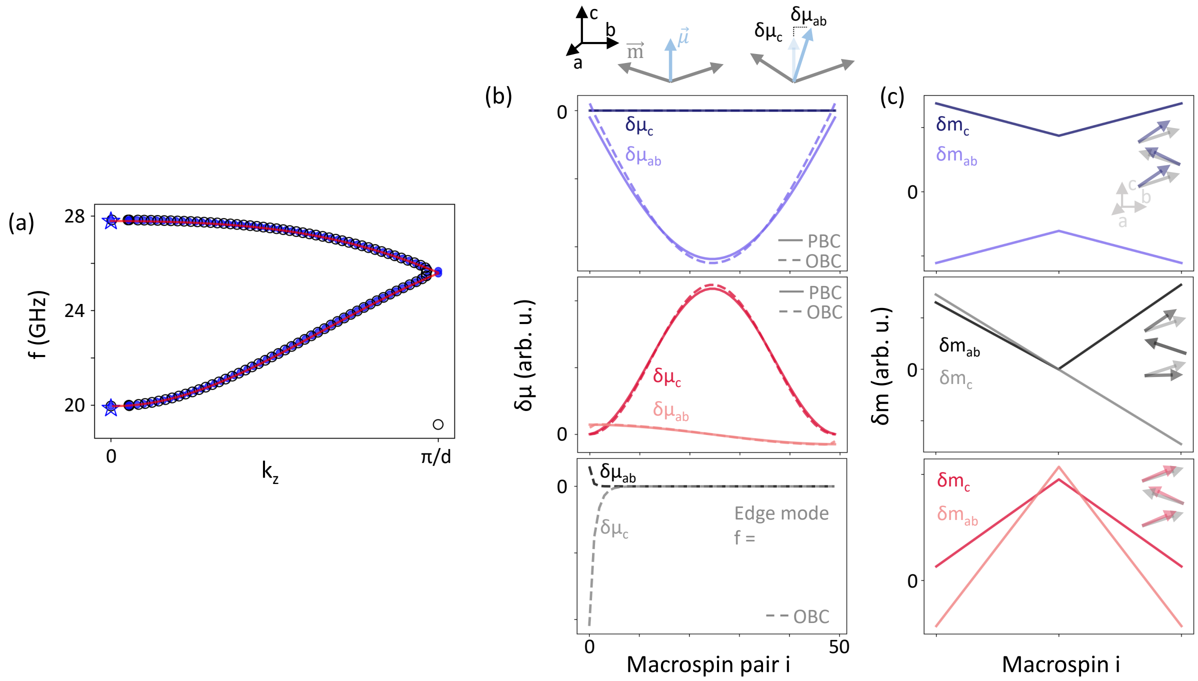

Supplementary Fig. 11: **Dispersion and mode shapes of the numerical macrospin model.** (a) Comparison of the eigenmodes for the $k_z = 0$ analytical model (stars), the analytical dispersion (red solid line) and the numerical model with periodic (blue filled circles) and open (black empty circles) boundary conditions. (b) The changes in the components of the magnetization vector $\vec{\mu}_i = \vec{m}_{2i-1} + \vec{m}_{2i}$ in the numerical macrospin model for the f_{IP} mode (top), the f_{OP} mode (middle) and an edge mode (bottom) for $N = 100$. Solid lines show the calculations with periodic boundary conditions and dashed lines for open boundary conditions. (c) The changes in the components of the macrospins $\vec{m}_i$ in the numerical macrospin model for the f_{IP} mode (top), the f_{OP} mode (middle) and the middle mode (bottom) for $N = 3$. Solid lines show the calculations with periodic boundary conditions and dashed lines for open boundary conditions.

Supplementary Note 7 Excitation of coherent magnon modes by laser pulses

Which of the modes in the numerical model do we observe in our experiment? In thick samples, previous studies have established that light pulses excite the optical and acoustic mode around $k_z \approx 0$ [8,9]. In Supplementary Fig. 11(b), we show the change in the components of the magnetization vectors $\vec{\mu}_i = \vec{m}_{2i-1} + \vec{m}_{2i}$ $(i = 1 - 50)$ of neighboring macrospins before and after excitation for the numerical model of 100 layers using periodic or open boundary conditions (PBC and OBC). The numerically calculated modes f_{IP} and f_{OP} indeed have minimal momentum k_z.

In thin samples, however, the spacing along the z-direction becomes discrete, so that the notion of a wavevector k_z loses meaning. Therefore, we need to establish which modes in the layer-resolved macrospin model correspond to the modes we excite and probe in the experiment in few-layer samples (and to what modes in bulk samples they correspond). We explicitly show the changes of the three individual macrospins $\delta\vec{m}_i$ for the trilayer in Supplementary Fig. 11(c) for all three eigenmodes (lowest to highest from top to bottom). We can directly see that the highest and lowest modes represent a symmetric change from the equilibrium macrospins (grey and colored arrows in inset sketches of Supplementary Fig. 11(c)). This is consistent with a homogeneous excitation across the sample, which we would expect as the result of a laser pulse. In the middle eigenmode, however, we can see that the change in the components $\delta m_{i,c}$ is asymmetric across the sample and would require an excitation mechanism that points in opposite directions for the macrospins in the top and bottom layers, while leaving the middle one unaffected. We therefore assume that the middle mode does not efficiently couple to light (equivalent to 'high k_z') and discard it for the analysis of our experimental data.

To extend these arguments to arbitrary layer numbers, we use the following set of assumptions to estimate the coupling of the eigenmodes to excitation by light:

- The ultrafast pump pulse leads to a step-like change in the layer-resolved effective fields:

$$\vec{H}_{\mathrm{eff}_j}(t > 0) = \vec{H}_{\mathrm{eff}_j}(t < 0) + \Delta\vec{H}_{\mathrm{eff}_j} \tag{30}$$

- This step-like change can be in the same direction across layers – e.g. due to heating decreasing the exchange interaction – or alternate in direction in every second layer – e.g. due to magnetoelastic coupling [10] (Supplementary Fig. 12(a)):

$$\Delta\vec{H}_{\mathrm{eff}_j} = \Delta\vec{H}_{\mathrm{eff}_{\mathrm{even}}} + (-1)^j \Delta\vec{H}_{\mathrm{eff}_{\mathrm{odd}}} \tag{31}$$

- The change in effective field leads to a new equilibrium macrospin position:

$$\vec{m}_{0,j}(t > 0) = \vec{m}_{0,j}(t < 0) + \Delta\vec{m}_j \tag{32}$$

- After the excitation, the macrospins want to align along their new equilibrium positions, leading to magnon oscillations. We use the projection of the eigenmodes before the laser pulse $\vec{\delta m}_j(t < 0)$ onto the $\Delta\vec{m}_j$ resulting from the aforementioned excitation as an estimate of how efficiently each eigenmode $\vec{\delta m}_j(t < 0)$ is excited by light:

$$\vec{\delta m}_j(t < 0) \cdot \Delta\vec{m}_j = \vec{\delta m}_j(t < 0) \cdot (\vec{m}_{0,j}(t > 0) - \vec{m}_{0,j}(t < 0)) \tag{33}$$

- We assume that $|m_{j,0}| = const.$, and therefore $\vec{m}_{j,0}(t < 0) \cdot \delta\vec{m}_j(t < 0) = 0$, so that the projection is simply given by

$$\vec{\delta m}_j(t < 0) \cdot \vec{m}_{0,j}(t > 0) \tag{34}$$

First, we model an excitation which is homogeneous across layers, changing the effective field by

$$\Delta\vec{H}_{\mathrm{eff}_{\mathrm{even,i}}} = -\Delta H_{\mathrm{E}_{ij}} \frac{\vec{H}_{\mathrm{E}_{ij}}}{|\vec{H}_{\mathrm{E}_{ij}}|}, \tag{35}$$

i.e. reducing the interlayer exchange in every layer. To account for heating effects, we include a gradient in $\Delta H_{\mathrm{E}_{ij}}$ (resulting layer-dependent exchange interaction after laser excitation in Supplementary Fig. 12(b)). As a result of the decreased exchange interaction, the equilibrium macrospin position after excitation points more along the $\hat{c}$-direction in all layers:

$$\vec{m}_{i,0}(t > 0) = \vec{m}_{i,0}(t < 0) + \Delta m_i \hat{c}. \tag{36}$$

Now, we calculate the projection $\delta\vec{m}_i(t < 0) \cdot \vec{m}_{i,0}(t > 0)$ for this case, shown colorcoded in the layer-dependent eigenvalues in Supplementary Fig. 12(c). For large N, the projection is largest for the lowest frequency bulk modes (yellow dots at $\sim$ 20 GHz), which is consistent with the f_{IP} of bulk measurements [8]. For small N, one of the edge modes starts to couple strongly to light as it spreads across the sample (light green dots with lowest f for $N < 10$), while the coupling of the former f_{IP} becomes weaker. Therefore we assign the corresponding eigenvalue of this edge mode to the experimental f_{IP} in few-layer devices.

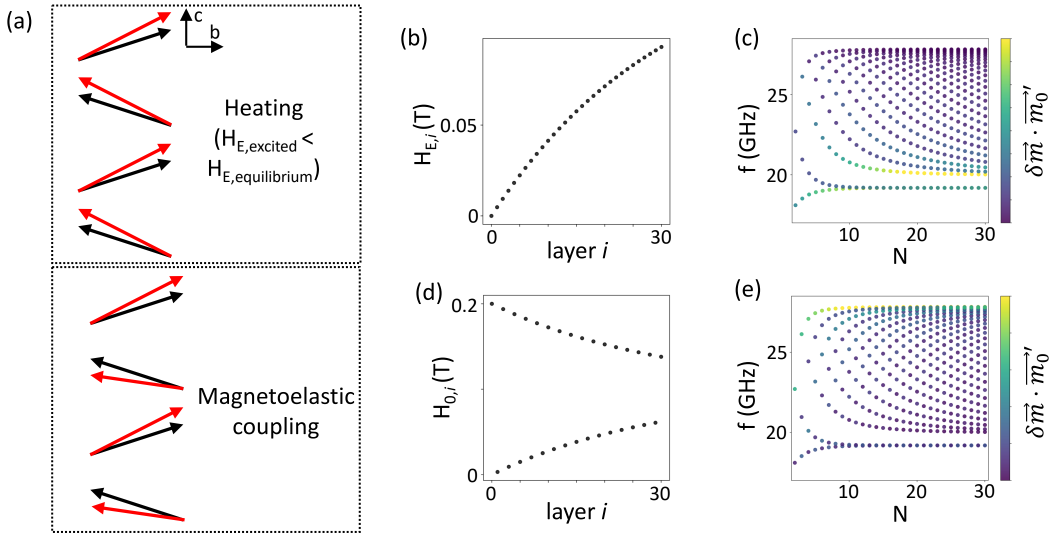

Supplementary Fig. 12: **Numerical macrospin model.** (a) Effect of a homogeneous change in H_{E} across layers, e.g. due to heating (top), or an alternating change in H_0 which is expected to arise from, e.g., magnetoelastic effects (bottom). Modeling this as a change in (b) $H_{\mathrm{E,i}}$ or (d) $H_{0,i}$ allows us to estimate the respective coupling of the modes in (c) and (e).

Next, we explore the effect of an alternating laser-pulse-induced change across layers, e.g. an additional effective field term along the c-direction due to magnetoelastic coupling [10]:

$$\Delta\vec{H}_{\mathrm{eff}_{\mathrm{odd,j}}} = (-1)^j \Delta H_{\mathrm{T}_j} \hat{c}. \tag{37}$$

As $\vec{H}_0 = H_0\hat{c}$ in our model, we introduce this additional effective field as a change in H_0, including a gradient due to heating effects (see Supplementary Fig. 12(d)). The new equilibrium macrospin positions after excitation now alternate

between layers:

$$\vec{m}_{0,j}(t > 0) = \vec{m}_{0,j}(t < 0) + (-1)^j \Delta m_j \hat{c}. \tag{38}$$

The resulting projections are shown colorcoded in Supplementary Fig. 12(e). The largest projection is seen for the highest frequency mode – corresponding to the f_{OP} ($k_z \approx 0$) mode in bulk experiments. For small N, the largest projection also belongs to the mode with the largest eigenvalue, which we therefore assign to our experimental f_{OP} (at $N = 3$ the bulk f_{IP} and f_{OP} modes converge). In Fig. 3b of the main text, the modes are colored according to the coupling to a homogeneous (blue) vs. alternating (red) excitation.

Supplementary Note 8 Gate-dependent magnetic phenomena

As mentioned in the main text, multiple gating effects on magnetic properties have been suggested as illustrated in Supplementary Fig. 13. (1) By increasing the electron doping n, the magnitude of the layer magnetization, i.e. macrospin length, can increase due to an increase in carriers with magnetic moment [11]. The Coulomb repulsion parameter U can increase with n, possibly influencing the exchange constants according to the Kugel-Khomskii model [12–14]. (2) When doping with electrons, e_g levels will be populated in addition to t_{2g} ones [13–15]. This can change the orbitals involved in the hopping between sites, again affecting the (anti-)ferromagnetic exchange coupling parameters. Additionally, the different orbital shapes can influence the anisotropy of the material [16]. (3) A perpendicular electric field between layers can shift the energy positons of bands associated with different orbitals in neighboring layers with respect to each other, again influencing the (anti-)ferromagnetic exchange coupling parameter [17]. (4) Gates can influence orbital filling especially in the outer layers due to the resulting electric field, as electrons shift further/closer to the gate, shuffling them into $d_{x^2-y^2}$ or d_{z^2} orbitals, respectively. This is commonly called voltage-induced magnetic anisotropy [18–20].

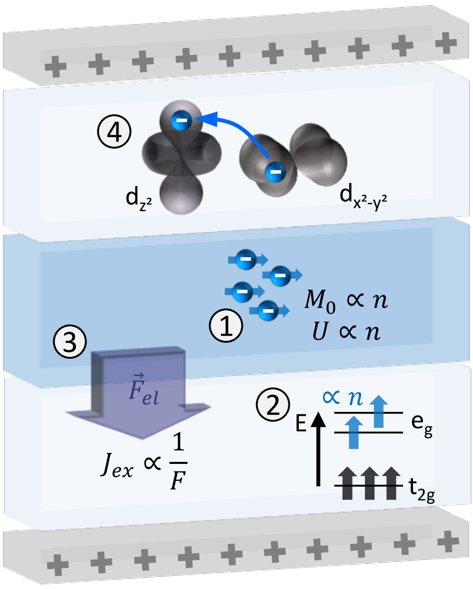

Supplementary Fig. 13: **Possible doping- and field related effects on magnetic parameters.** Sketch of possible gating effects on layer magnetization, anisotropy and exchange fields.

Supplementary Note 9   Macrospin fit results

Using the dependencies from the main text and the results from Supplementary Fig. 6, we fit the lowest and highest eigenvalue of Eq. (29) to the observed gate-dependent f_{IP} and f_{OP} of the trilayer. We use the resulting fitting parameters from Table 1 of the main text to calculate the interlayer exchange, intermediate axis anisotropy fields and macrospin lengths for each layer in Supplementary Fig. 14.

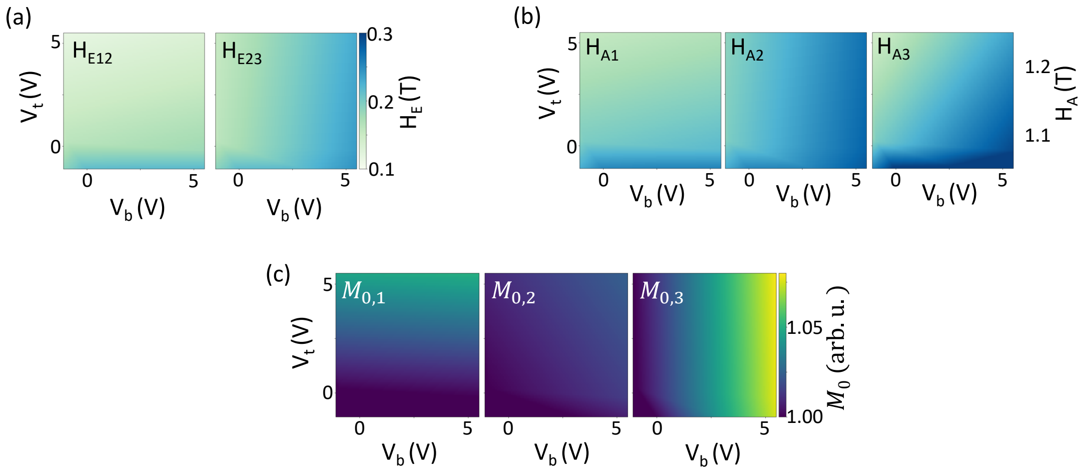

Supplementary Fig. 14: **Macrospin fitting results trilayer.** The results of the macrospin model fitted to the capacitor model for the (a) exchange fields, (b) anisotropy fields and (c) gyromagnetic ratios.

The tunability we reach in the interlayer exchange field is comparable to that in bulk CrSBr when applying in-plane strain [21], however, the exchange interaction becomes stronger rather than weaker. Our dependence of anisotropy on electric field of $-0.85\,\mathrm{T\,nm\,V^{-1}}$ corresponds to $\sim -600\,\mathrm{fJ\,V^{-1}\,m^{-1}}$ of changes in anisotropy constant per unit surface per unit electric field. For comparison, usual voltage controlled magnetic anisotropy (VCMA) coefficients describing changes of interfacial perpendicular magnetic aniostropy at ferromagnet-oxide interfaces reach around $\sim -100\,\mathrm{fJ\,V^{-1}\,m^{-1}}$ [20, 22, 23]. The maximum changes in the macrospin length we predict in the top layer of $\sim 8\%$, which is in the range of what has been predicted as a doping-dependent change of the magnetic moment in CrSeBr [11]. We do the same calculations for the 5-layer device using the results of the electrostatic modelin in Supplementary Fig. 8 and the fitting parameters from Table 1 of the main text. The resulting internal fields and gyromagnetic ratios are shown in Supplementary Fig. 15.

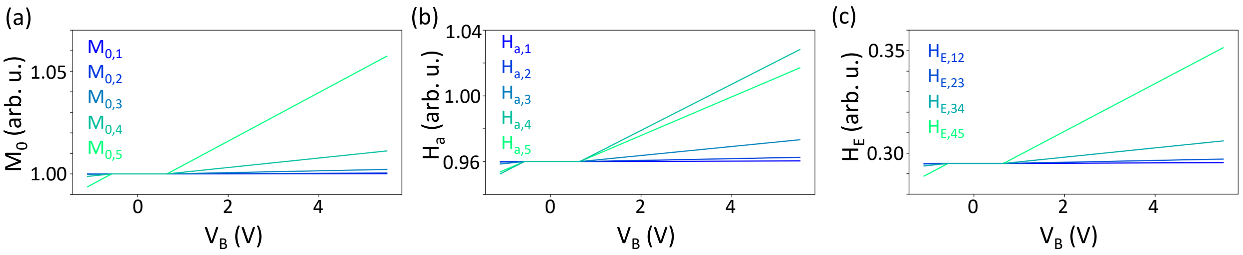

Supplementary Fig. 15: **Macrospin fitting results 5-layer.** The results of the macrospin model fitted to the capacitor model for the (a) macrospin lengths, (b) anisotropy fields and (c) interlayer exchange fields.

To elucidate why we chose the dependencies on n and F shown in the main text, we briefly discuss different possibilities

to fit the trilayer data to the macrospin model (physical processes leading to these dependencies are discussed in Section Supplementary Note 8). The most intuitive consequences of an increased electron density in the sample are an increase of the layer magnetization as well as an increased interlayer exchange interaction. Let us therefore start by fitting the trilayer data only considering doping-dependent linear effects, explicitly $M_{0,i} \propto n_i$, $H_{a,i} \propto n_i$ and $H_{\mathrm{E},ij} \propto n_i + n_j$. We show the difference between experimental and fitted magnon frequencies $f_{\mathrm{ex}} - f_{\mathrm{mod}}$ in Supplementary Fig. 16(a). In the resulting Voronoi plots, white/faint colors correspond to small differences between the fit and the experimental plot, while bright red/blue signify larger deviations. Unsurprisingly, the resulting fits fail to capture any field-dependent changes of the magnon frequencies. We conclude that at least one of the magnetic properties should be dependent on the perpendicular electric field F_{z}.

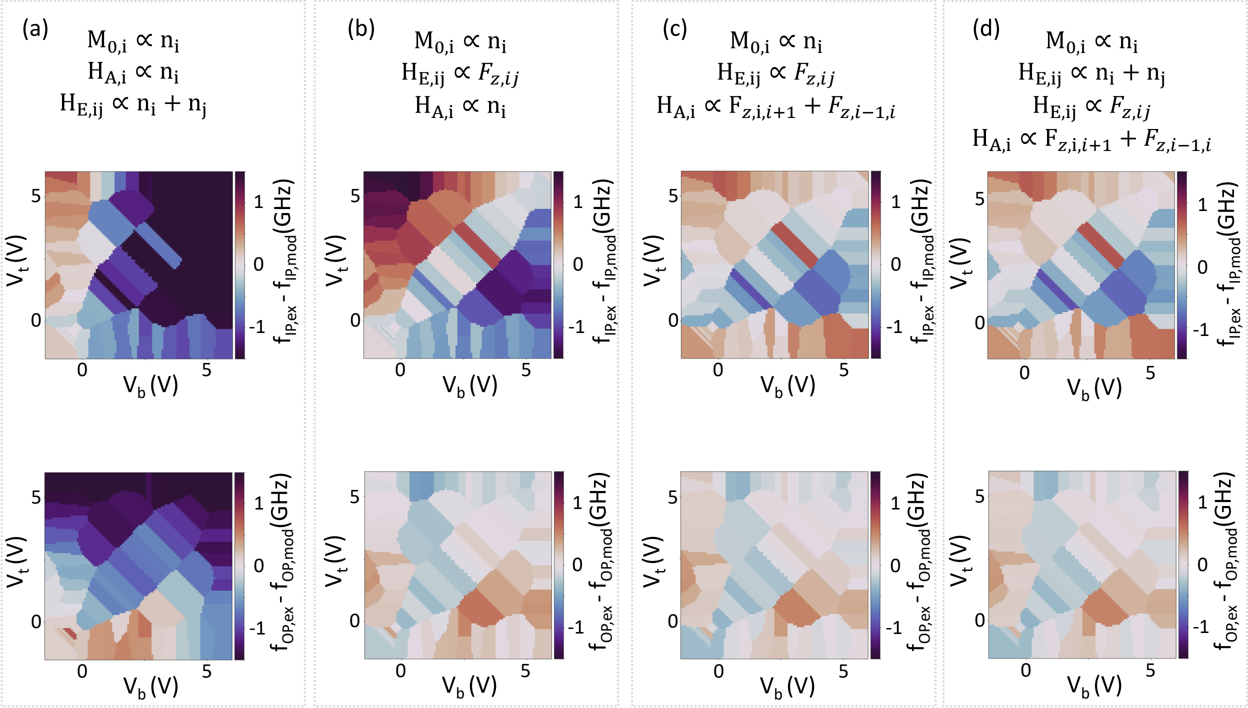

Supplementary Fig. 16: **Macrospin fitting approaches to the trilayer data.** Comparison of experimental $f_{\mathrm{IP,ex}}$ and $f_{\mathrm{OP,ex}}$ with the fitted $f_{\mathrm{IP,mod}}$ and $f_{\mathrm{OP,mod}}$ for different n- and F-dependencies of magnetic parameters (shown at the top).

Therefore, we choose an electric field dependence of the interlayer exchange interaction in the next fit generation, i.e. $M_{0,i} \propto n_i$, $H_{a,i} \propto n_i$ and $H_{\mathrm{E},ij} \propto F_{\mathrm{z},ij}$ (Supplementary Fig. 16(b)). To avoid overfitting, we neglect the doping dependence of $H_{\mathrm{E},ij}$. This model indeed fits the gate dependence of the f_{OP} mode better. However, it still does not capture the field dependence of the lower magnon mode. This leads us to believe that in order to fit the field-dependence of both modes, we need to include two competing parameters $\propto F_{\mathrm{z},ij}$.
We therefore include a second field-dependence, choosing $M_{0,i} \propto n_i$, $H_{a,i} \propto F_{\mathrm{z},i,i+1} + F_{\mathrm{z},i,i-1}$ and $H_{\mathrm{E},ij} \propto F_{\mathrm{z},ij}$. This captures the gate-dependencies of both f_{IP} and f_{OP} reasonably well (Supplementary Fig. 16(c)). The average difference between the modeled and experimental f_{OP} is < 0.2 GHz. The largest deviations can be seen in the low carrier density regime around $V_{\mathrm{b}} = V_{\mathrm{t}} = 0 - 2\,\mathrm{V}$. This is due to the delayed onset of the frequency shifts also visible in Fig. 1(c) of the main manuscript. For f_{IP}, we can see that at high absolute field values F_{z} the fits deviate the most from the experimental behavior (up to 0.7 GHz), possibly indicating an additional phenomenon taking effect at those

gate voltages. However, we also note that in the measurements on the trilayer device, the lower frequency mode had a weaker signature in the FFT, leading to a larger error when extracting its position. Overall, the average difference between the experimental and modeled f_{IP} is < 0.3 GHz.

Lastly, we examine whether including an additional doping dependence of the exchange interaction improves the fits further using $M_{0,i} \propto n_i$, $H_{a,i} \propto F_{z,i,i+1} + F_{z,i,i-1}$, $H_{\mathrm{E},ij} \propto n_i + n_j$ and $H_{\mathrm{E},ij} \propto F_{z,ij}$ (Supplementary Fig. 16(d)). We see no significant improvements in the fits compared to those in Supplementary Fig. 16(c). We therefore neglect any dependencies beyond those shown in Supplementary Fig. 16(c) to avoid high fitting errors resulting from overfitting.

Supplementary Note 10 Gate dependence of magnons in bilayer devices

We have measured the gate-dependence on two bilayer samples. The first, singlegated, sample also has regions with more layers (Supplementary Fig. 17(a)). We observe a strong drop in the signal intensity when going to the bilayer region (Supplementary Fig. 17(b)). Additionally, the higher frequency mode f_{OP} decreases (Supplementary Fig. 17(c)), in accordance with the prediction of the macrospin model (Supplementary Fig. 17(d)). We also observe on this sample that the tunability of the high frequency mode decreases by an order of magnitude going from the bilayer region of the sample to the 4-layer area in Supplementary Fig. 17(e).

The bottom-gate dependence of f_{IP} and f_{OP} in the singlegated bilayer is shown in Supplementary Fig. 18(a). We find good agreement with the predictions of the macrospin model. In a second, dual-gated bilayer sample, we find a doping-induced shift of f_{IP} of $\sim$ 2 GHz and $\sim$ 4 GHz for f_{OP}, also in good agreement with the macrospin fits (Supplementary Fig. 18(b)).

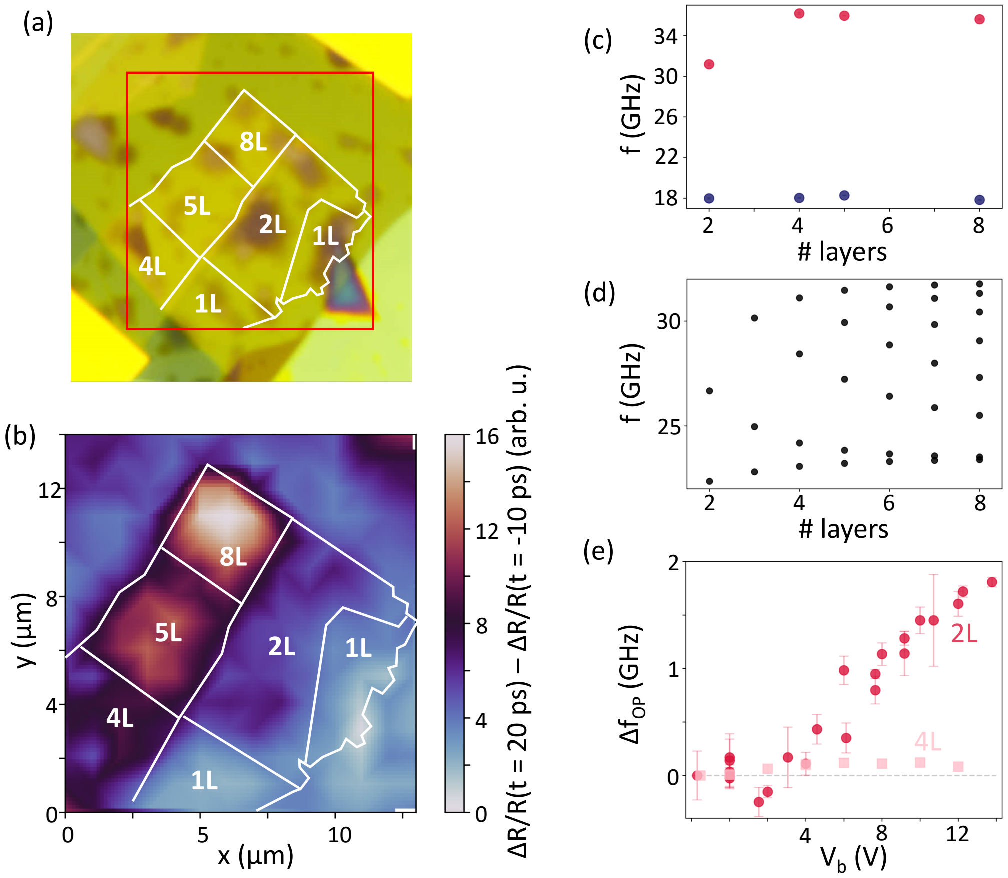

Supplementary Fig. 17: **Thickness-dependence of magnons.** (a) Optical image and (b) strength of the tr-reflectivity signal at 20 ps on a sample with different thickness steps. (c) Layer-number dependent magnon frequencies measured on the different areas in (a,b). (d) Modelled layer-number dependence of magnon frequencies in the numerical macrospin model. (e) V_b-dependence of the change in the f_{OP} mode measured on the 2- and 4-layer regions in (a,b).

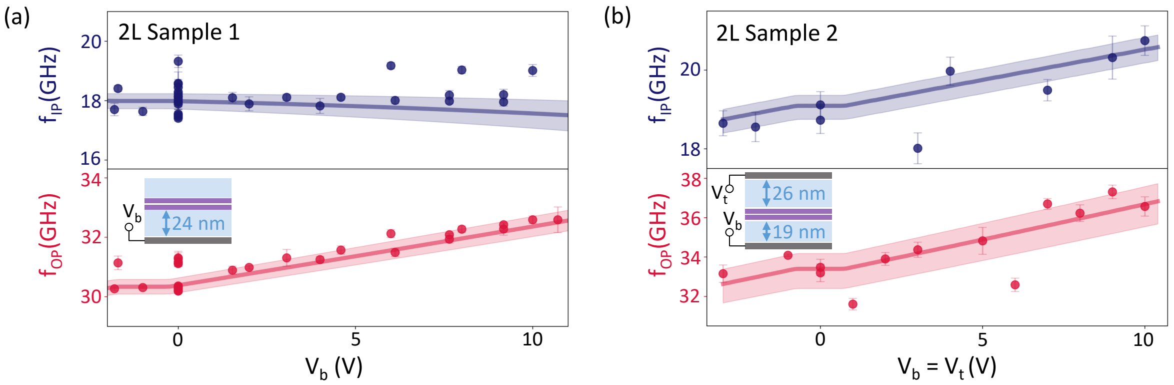

Supplementary Fig. 18: **Gate-dependence of magnons in bilayer samples.** (a) V_b-dependence of f_{IP} and f_{OP} in the single-gated bilayer sample (schematic in inset). (b) $V_b = V_t$-dependence of f_{IP} and f_{OP} in the dual-gated bilayer sample (schematic in inset). Solid lines show the macrospin fits.

References

[1] Evan J. Telford, Avalon H. Dismukes, Raymond L. Dudley, Ren A. Wiscons, Kihong Lee, Daniel G. Chica, Michael E. Ziebel, Myung Geun Han, Jessica Yu, Sara Shabani, Allen Scheie, Kenji Watanabe, Takashi Taniguchi, Di Xiao, Yimei Zhu, Abhay N. Pasupathy, Colin Nuckolls, Xiaoyang Zhu, Cory R. Dean, and Xavier Roy. Coupling between magnetic order and charge transport in a two-dimensional magnetic semiconductor. *Nature Materials*, 21:754–760, 2022.

[2] David C. Meeker. Finite Element Method Magnetics, version 4.2 (https://www.femm.info).

[3] Riccardo Pisoni, Tim Davatz, Kenji Watanabe, Takashi Taniguchi, Thomas Ihn, and Klaus Ensslin. Absence of interlayer tunnel coupling of K-valley electrons in bilayer MoS_2. *Physical Review Letters*, 123:117702, 2019.

[4] Sviatoslav Kovalchuk, Kyrylo Greben, Abhijeet M. Kumar, Simon Pessel, Jan Soyka, Qing Cao, Kenji Watanabe, Takashi Taniguchi, Dominik Christiansen, Malte Selig, Andreas Knorr, Siegfried Eigler, and Kirill I. Bolotin. Revealing hidden interlayer excitons in 2D bilayers via hybrid molecular gating. *Nature Communications*, 16:9893, 2025.

[5] Akash Laturia, Maarten L. Van de Put, and William G. Vandenberghe. Dielectric properties of hexagonal boron nitride and transition metal dichalcogenides: from monolayer to bulk. *npj 2D Materials and Applications*, 2:1–7, 2018.

[6] Julian Klein, Benjamin Pingault, Matthias Florian, Marie Christin Heißenbüttel, Alexander Steinhoff, Zhigang Song, Kierstin Torres, Florian Dirnberger, Jonathan B. Curtis, Mads Weile, Aubrey Penn, Thorsten Deilmann, Rami Dana, Rezlind Bushati, Jiamin Quan, Jan Luxa, Zdeněk Sofer, Andrea Alù, Vinod M. Menon, Ursula Wurstbauer, Michael Rohlfing, Prineha Narang, Marko Lončar, and Frances M. Ross. The bulk van der Waals layered magnet CrSBr is a quasi-1D material. *ACS Nano*, 17:5316–5328, 2023.

[7] Marie Christin Heißenbüttel, Pierre Maurice Piel, Julian Klein, Thorsten Deilmann, Ursula Wurstbauer, and Michael Rohlfing. Quadratic optical response to a magnetic field in the layered magnet CrSBr. *Physical Review B*, 111:075107, 2025.

[8] Youn Jue Bae, Jue Wang, Allen Scheie, Junwen Xu, Daniel G. Chica, Geoffrey M. Diederich, John Cenker, Michael E. Ziebel, Yusong Bai, Haowen Ren, Cory R. Dean, Milan Delor, Xiaodong Xu, Xavier Roy, Andrew D. Kent, and Xiaoyang Zhu. Exciton-coupled coherent magnons in a 2D semiconductor. *Nature*, 609:282–286, 2022.

[9] Yue Sun, Fanhao Meng, Changmin Lee, Aljoscha Soll, Hongrui Zhang, Ramamoorthy Ramesh, Jie Yao, Zdeněk Sofer, and Joseph Orenstein. Dipolar spin wave packet transport in a van der Waals antiferromagnet. *Nature Physics*, 20:794–800, 2024.

[10] Youn Jue Bae, Taketo Handa, Yanan Dai, Jue Wang, Huicong Liu, Allen Scheie, Daniel G. Chica, Michael E. Ziebel, Andrew D. Kent, Xiaodong Xu, Ka Shen, Xavier Roy, and Xiaoyang Zhu. Transient magnetoelastic coupling in CrSBr. *Physical Review B*, 109:104401, 2024.

[11] Ruilin Han, Xiaomin Xue, and Peng Li. Enhanced ferromagnetism, perpendicular magnetic anisotropy and high Curie temperature in the van der Waals semiconductor CrSeBr through strain and doping. *Physical Chemistry Chemical Physics*, 26:12219–12230, 2024.

[12] Farsane Tabataba-Vakili, Huy P. G. Nguyen, Anna Rupp, Kseniia Mosina, Anastasios Papavasileiou, Kenji Watanabe, Takashi Taniguchi, Patrick Maletinsky, Mikhail M. Glazov, Zdenek Sofer, Anvar S. Baimuratov, and Alexander Högele. Doping-control of excitons and magnetism in few-layer CrSBr. *Nature Communications*, 12 2023.

[13] K. I. Kugel and D. I. Khomskii. Crystal structure and magnetic properties of substances with orbital degeneracy. *Zh. Eksp. Teor. Fiz.*, 64:1429–1439, 1973.

[14] K. I. Kugel and D. I. Khomskii. The Jahn-Teller effect and magnetism: Transition metal compounds. *Soviet Physics - Uspekhi*, 25:621–641, 1982.

[15] V. V. Mazurenko, F. Mila, and V. I. Anisimov. Electronic structure and exchange interactions of $Na_2V_3O_7$. *Physical Review B*, 73:014418, 2006.

[16] Michael E. Ziebel, Margalit L. Feuer, Jordan Cox, Xiaoyang Zhu, Cory R. Dean, and Xavier Roy. CrSBr: An air-stable, two-dimensional magnetic semiconductor. *Nano Letters*, 24:4319–4329, 2024.

[17] Youwen Wang, Nannan Luo, Jiang Zeng, Li Ming Tang, and Ke Qiu Chen. Magnetic anisotropy and electric field induced magnetic phase transition in the van der Waals antiferromagnet CrSBr. *Physical Review B*, 108:054401, 2023.

[18] T. Maruyama, Y. Shiota, T. Nozaki, K. Ohta, N. Toda, M. Mizuguchi, A. A. Tulapurkar, T. Shinjo, M. Shiraishi, S. Mizukami, Y. Ando, and Y. Suzuki. Large voltage-induced magnetic anisotropy change in a few atomic layers of iron. *Nature Nanotechnology*, 4:158–161, 2009.

[19] K. H. He, J. S. Chen, and Y. P. Feng. First principles study of the electric field effect on magnetization and magnetic anisotropy of FeCo/MgO(001) thin film. *Applied Physics Letters*, 99:72503, 2011.

[20] Bivas Rana, Samiran Choudhury, Katsuya Miura, Hiromasa Takahashi, Anjan Barman, and Yoshi Chika Otani. Electric field control of spin waves in ultrathin CoFeB films. *Physical Review B*, 100:224412, 2019.

[21] Geoffrey M. Diederich, John Cenker, Yafei Ren, Jordan Fonseca, Daniel G. Chica, Youn Jue Bae, Xiaoyang Zhu, Xavier Roy, Ting Cao, Di Xiao, and Xiaodong Xu. Tunable interaction between excitons and hybridized magnons in a layered semiconductor. *Nature Nanotechnology*, 18:23–28, 2022.

[22] Xiang Li, Kevin Fitzell, Di Wu, C. Ty Karaba, Abraham Buditama, Guoqiang Yu, Kin L. Wong, Nicholas Altieri, Cecile Grezes, Nicholas Kioussis, Sarah Tolbert, Zongzhi Zhang, Jane P. Chang, Pedram Khalili Amiri, and Kang L. Wang. Enhancement of voltage-controlled magnetic anisotropy through precise control of Mg insertion thickness at CoFeB|MgO interface. *Applied Physics Letters*, 110:52401, 2017.

[23] Takayuki Nozaki, Kay Yakushiji, Shingo Tamaru, Masaki Sekine, Rie Matsumoto, Makoto Konoto, Hitoshi Kubota, Akio Fukushima, and Shinji Yuasa. Voltage-induced magnetic anisotropy changes in an ultrathin FeB layer sandwiched between two MgO layers. *Applied Physics Express*, 6:073005, 2013.
