# Supplementary material for: Tunable magnons in a dual-gated 2D antiferromagnet
Source: Nat Commun. 2026 Jun 12;17:5262. doi: 10.1038/s41467-026-74067-z (PMC13263320; doi:10.1038/s41467-026-74067-z)
Supplement: Supplementary file 2 — Transparent Peer Review file [file 41467_2026_74067_MOESM2_ESM.pdf]

nature portfolio

Peer Review File

Tunable magnons in a dual-gated 2D antiferromagnet

Corresponding Author: Ms Nele Stetzuhn

This file contains all reviewer reports in order by version, followed by all author rebuttals in order by version.

Version 0:

Reviewer comments:

Reviewer #1

(Remarks to the Author)
This manuscript investigates the impact of electrostatic gating on the magnetic properties and magnon dynamics of thin-layer CrSBr systems. The authors use a layer-resolved macrospin model to interpret the evolution of magnon modes under dual-gating conditions, distinguishing between in-phase and out-of-phase magnon responses. The work is clearly written and generally well-structured, reflecting substantial experimental effort and thoughtful theoretical modeling. However, I have serious concerns about the data analysis and interpretation, particularly regarding the model's agreement with experimental results. These issues must be thoroughly addressed before the work can be considered for publication.

1. In Fig. 1(c), the macrospin model fails to accurately capture the experimental trends of magnon frequencies (f_IP and f_OP) as a function of gate voltage (Vb = Vt), particularly below 2 V. This discrepancy indicates that the model may not sufficiently account for low-carrier-density regimes or nonlinear effects in this voltage range. A more detailed discussion of the model's limitations and potential physical mechanisms behind this deviation would enhance the interpretation of the results.

2. Both Fig. 1(c) and Fig. 2(d) show a crossing point of the magnon modes and carrier densities around 2 V. Is this a coincidence, or does it imply a critical carrier density distribution between the top (n_t) and bottom (n_b) layers? This aspect deserves explicit discussion in the manuscript. If this behavior arises from a specific balance in layer-resolved carrier densities, the authors should clarify its implications for the magnon spectrum.

3. In Fig. 4, the agreement between experimental data and model predictions—particularly for f_OP in the 5-layer system—is poor. The data indicate minimal tunability of f_OP with bottom gate voltage, contradicting model expectations. The authors should clarify whether interlayer interactions are significantly weakened or screened in thicker samples, and how this affects the validity of their model assumptions.

4. The model employs a linear dependence (Eqs. 3–5) to describe gate-induced changes in magnetic parameters, but the physical justification for this assumption is unclear. According to Table I, the magnitude of these changes—such as an interlayer exchange shift of ~1 T—appears unreasonably large compared to the zero-field coupling (H_E0 ~0.2 T), suggesting the linear response regime has been exceeded. A detailed discussion on the validity of this approximation is essential, including whether nonlinear or saturation effects should be taken into account.

Reviewer #2

(Remarks to the Author)

The manuscript entitled "Tunable magnons in a dual-gated 2D antiferromagnet" reports an investigation of the magnetization dynamics eigenmodes (magnons) in a gated trilayer of the prototypical van der Waals antiferromagnetic semiconductor – CrSBr. The experiments employ optically detected excitonic and trionic resonances to probe the magnetic oscillations as well as the effect of the gate voltages. The authors find that the magnetic eigenmode frequencies depend on the voltages applied to the top and bottom gates. This dependence is most pronounced for one particular magnon mode in the trilayer and becomes weak for thicker samples. The authors employ a theoretical model that treats the typical magnetic characteristics of the material, such as anisotropy, to be layer dependent and as fitting parameters.

The observed tunability of the magnon modes with respect to the gate voltage is intriguing and a useful property of such van der Waals magnets. Its observation in the air stable material CrSBr is especially exciting and valuable. Hence, I find the main message and goal of the article to fit the scope of Nature Communications. However, at present, I am having trouble digesting some of the data interpretation and the corresponding conclusions drawn by comparing to the theoretical model. I find it important to clarify the following scientific issues before I can make a clear recommendation.

1. The authors have employed oscillations in the transient reflectivity as a direct signature and measure of the magnon modes. A similar attribution has indeed been done in previous literature, e.g., Ref. [21]. However, this attribution stems from a specific interaction between the magnon and the exciton in question, and requires the CrSBr to be in a canted magnetic ground state. I guess that this is why the authors also need to subject their sample to a permanent magnet and applied magnetic field, so that a canted magnetic state is achieved in equilibrium.

The manuscript does not seem to clarify how their transient reflectivity maps to magnon modes. What is the nature of the excitons and trions that allows this relation between the reflectivity and magnons?

2. Continuing along the same direction as above, the excitonic modes that have been used to detect coherent magnons (such as in Ref. [21]) obtain a varying degree of interlayer character with the magnetic canting (see Ref. [22], for example). This also affects their oscillator strength as discussed in PRB 111, 075107 (2025), for example. Both of these features, varying oscillator strength and interlayer character, have not been accounted for in making inferences in the present manuscript. Instead the authors seem to treat their excitons and trions as intralayer, whose detectability is assumed to be related only to the charge doping. Is this justifiable?

3. In Fig. 1(c), the comparison between theory and experiment is not good enough to draw conclusions of agreement. The authors should examine and comment on this point.

4. The seemingly good agreement between figures 1(d) and (f) [1(e) and (g)] also relies on several ad hoc fitting parameters for each layer, and as such should be considered more carefully. First, from symmetry, we would expect that a trilayer sandwiched between two symmetric gates should respond in the same manner to voltages applied to the top and bottom gates. The experiments however find a strong asymmetry in how the magnon modes respond to the voltages in the two gates, as per Fig. 1(d) and (e). This requires a more detailed justification before going to a phenomenological model. This asymmetry could also be a consequence of the excitons being interlayer.

5. In Fig. 3(b), the authors find three magnon modes from their numerical theoretical modeling. However, the experiment only finds two modes, consistent with the analytic model. The authors resolve this issue by disregarding the magnon mode with the middle frequency. Can this be justified on physical grounds? If not, can we rely on the numerical theoretical model which does not agree with the experiment about the number of magnon modes?

6. In assuming the phenomenological fitting equations (3) – (5), the authors argue the feasibility of the assumed dependencies on the charge density and electric field. This discussion is somewhat counterintuitive. For example, I would expect that the exchange interaction depends on the charge density which determines the screening of Coulomb interaction, as also briefly mentioned by the authors. Instead, the authors assume the exchange to depend only on the electric field, and not the charge density. At the same time, the assumed dependence of the gyromagnetic ratio on the charge density seems hard to justify. The authors state “the position of the chemical potential – controlled by n – affects the imbalance between the occupied spin-up and spin-down states, affecting the magnetic moment μ and in turn the gyromagnetic ratio γ of the corresponding macrospin”. However, the imbalance between occupied states should affect only the magnetic moment, but not the gyromagnetic ratio which is the ratio between the magnetic moment and spin. The spin should also change with the changing magnetic moment to maintain the gyromagnetic ratio.

Overall, I see bit of a gap between the phenomenological dependencies assumed to fit the experimental data and the physical expectations, suggesting further discussion on this issue.

Reviewer #3

(Remarks to the Author)
Stetzuhn et al. demonstrated the gate-tunable magnons of CrSBr in a dual-gated configuration, and developed a model to explain the results, establishing the doping- and electric field-dependence of the exchange interaction, magnetic anisotropy and magnetic moment of individual layers. Overall, the topic is of interest in the field 2D magnetics, the results are solid and the explanations seems reasonable. My concerns focus on the novelty of both motivation and the proposed model: what are the differences between the previous and present work? My detailed comments are as following, and the editor makes the final decision.
(1) The core of the manuscript is the tunability of magnon by electric gating. Given that the effect of gating on magnon energies and transport has been studied in other magnetic systems, the novelty of the present manuscript may not reach the standard of Nat. Commun. I suggest the authors describe the difference a little more in the Introduction part.
(2) The range of the tunability of the magnon modes is only 10%. How does this value reflect the high tunability? What is the potential application of magnon in this narrow range? Besides, the in-plane frequency seems to be insensitive to the gating, so what is new here?
(3) The authors claim that they proposed a model to explain the data. I am wondering that the model is just a modified model or a new model proposed for the first time? Moreover, for the gate-tunable magnon in other magnetic materials, what kinds of

models do they use? Are there any differences between the models in the present and previous work?
(4) The authors chose the flakes with layer number of 3-8 layers. Why not choose bilayer flake? In my opinion, the doping of individual layer in bilayer CrSBr may be more suitable for the explanation the authors proposed.
(5) For the fitting, the authors used the calculated value of dielectric constant of CrSBr, and the authors used the fitting results to demonstrate the accuracy of their models. Given that this value is strongly dependent on the layer number, and the calculated value may be inconsistent with the experimental value, I suggest the authors give an experimental value of dielectric constant, and its layer-dependence for fitting. This is more reasonable, I think.
(7) The experimental results in Figures 1d, e are quite different from the simulated counterparts in Figures 1f, g. I am confused that why the authors claim that the experimental data are in good agreement with the theoretical values?
(8) In Figure 2a, there is an intensity drop when Vb = Vt = ~ -0.6 V. How to explain it? Besides, given that the authors estimated the carrier density of top layer, middle layer and bottom layer in Figure 2d, the value of individual layer is accurate or not? If the layer number is larger than 3, is it reasonable to estimate the carrier density or magnetic moment of individual layer for further explanation or fitting?
(9) There are some typos, especially in References part. The authors should carefully check them.

Reviewer #4

(Remarks to the Author)
The manuscript by Nele Stetzuhn et al. reports electrically tunable magnons in the dual-gated 2D antiferromagnet CrSBr, with frequencies reaching up to 2 GHz. By employing a time-resolved pump–probe technique, the authors systematically characterize magnon frequencies under varying doping levels and electric-field orientations. The observed frequency shifts with carrier density are attributed to a layer-dependent gyromagnetic ratio, interlayer exchange interactions, and magnetic anisotropy. Magnons in CrSBr are of considerable current interest in the field of antiferromagnetic materials. Nevertheless, several issues should be addressed before the manuscript can be considered for publication in Nature Communications.
1) The authors report two oscillation frequencies of the magnon at 19 and 28 GHz, corresponding to the in-phase and out-of-phase zero-momentum magnon modes. However, according to Fig. 3 of Ref. [Phys. Rev. B 109, 104401], the optical (Op) and acoustic (Ac) interlayer AFM magnon modes appear at 24.6 and 33.4 GHz, respectively. Could the authors clarify the origin of this discrepancy in magnon frequencies? Additionally, the cited reference employs MOKE to determine the magnon modes, whereas the present work uses a time-resolved pump–probe technique. What are the differences between these two approaches in determining the magnon modes?
2) In CrSBr, the reported frequency shift under dual-gate tuning is around 2 GHz. By comparison, in CrI_3 the magnon frequency can be tuned from 80 to 55 GHz when the gate voltage is varied from –13 to 13 V [Nature Materials volume 19, pages838–842 (2020)]. What, then, is the specific advantage of CrSBr under gate tuning? I suggest that the authors clarify the significance of this result and emphasize it in the Introduction."
3) As variations in carrier concentration are generally expected to influence the spin–exchange interaction and thereby affect the magnon frequency, could the authors clarify how this aspect has been considered in their analysis?
4) In Fig. 2a, the PL emission appears to suddenly drop around –1 eV. As reported in Ref. (Nat. Commun. 15, 4735 (2024)), the XB and XB^- emissions gradually disappear with increasing reverse voltage. Could the authors clarify what happens at this point?"
5) Minor typos need revision; for instance, 'their' appears twice on line 29, page 1.

Version 1:

Reviewer comments:

Reviewer #1

(Remarks to the Author)
The revised manuscript includes new data, a modified macrospin model, and additional discussions in response to the reviewers' comments, most concerns appear to have been addressed satisfactorily. However, the lower panel of Fig. 2(d) in the revised version differs noticeably from that in the original submission. The authors should clearly explain the modifications and justify this change before a definitive recommendation can be made.

Reviewer #2

(Remarks to the Author)
The authors have adequately addressed my comments through additional measurements, theoretical analysis and clarifications. I now find the manuscript to be essentially ready for publication in Nature Communications.

I have one remaining question, which did not become fully clear from the authors' response and that the authors could consider clarifying:

Figure 1b describes how the transient reflectivity data is converted to magnon frequencies, by attributing the oscillations to magnetization dynamics. This has been done using the dependence of the exciton energy on the magnetic canting, as the authors have further clarified in their previous response. However, in Fig. 2, the authors show closely spaced exciton and

trion peaks in the PL spectrum. Now, do the trions play any role in the data of Fig. 1b? Or can one completely disregard the trions there? If the trions cannot be disregarded, how does one expect the dependence of the trion energy on the magnetic configuration to be? In the authors' response to my related question in the last round, they only discussed excitons and exciton-magnon coupling, as far as I can tell.

Reviewer #3

(Remarks to the Author)
I have carefully read the revised manuscript and the response, and greatly appreciate the efforts from the authors. My comments have been addressed, and I recommend it for publication as is.

Reviewer #4

(Remarks to the Author)
After reviewing the revised manuscript, I am generally satisfied with the authors' responses. However, there are still several issues that remain unclear or difficult to follow:
1. In Figure 2b, the gray curve is labeled as n = 0. I do not think this is an appropriate description. Although the Fermi level lies within the bandgap in this case, the carrier density n is not strictly zero. The authors are encouraged to adopt a more accurate expression.
2. On page 2, the authors state:
"Around 1.34 eV, we find an amalgamation of several weaker peaks even in the absence of trions (as n = 0), which can be assigned to the XA exciton (transition between the top valence band and the bottom conduction band) and phonon replicas [15,22,47,48]."
This suggests that the peaks around 1.34 eV originate from XA exciton and phonon replicas without doping. However, in Figure 2, the relative intensities of the gray and purple (yellow) curves appear to be of the same order of magnitude. Therefore, it is unclear whether the contribution from XA has been excluded when evaluating the intensities of XB^{-} and XB'^{-}. The authors should clarify this point.

Version 2:

Reviewer comments:

Reviewer #1

(Remarks to the Author)
My comments have been addressed, and I recommend this work for publication.

Reviewer #2

(Remarks to the Author)
The authors have adequately addressed my last remaining comment. I recommend the publication of the revised manuscript in Nature Communications.

Reviewer #4

(Remarks to the Author)
I am satisfied with the authors' response and recommend publication in Nature Communications.

Open Access This Peer Review File is licensed under a Creative Commons Attribution 4.0 International License, which permits use, sharing, adaptation, distribution and reproduction in any medium or format, as long as you give appropriate credit to the original author(s) and the source, provide a link to the Creative Commons license, and indicate if changes were made.
In cases where reviewers are anonymous, credit should be given to 'Anonymous Referee' and the source.
The images or other third party material in this Peer Review File are included in the article's Creative Commons license, unless indicated otherwise in a credit line to the material. If material is not included in the article's Creative Commons license and your intended use is not permitted by statutory regulation or exceeds the permitted use, you will need to obtain permission directly from the copyright holder.
To view a copy of this license, visit https://creativecommons.org/licenses/by/4.0/

We sincerely thank the editor and all the reviewers for the valuable feedback provided in their responses. First, we would like to summarize general changes in the manuscript, in particular to improve the predictive power of our model. Motivated by the reviewers' comments, we have:

- Conducted additional experiments on the gate-dependent tr-reflectivity and photoluminescence of two newly fabricated bilayer CrSBr samples.
- Altered the macrospin model, in particular to include the anisotropy field H_b and the doping-dependent magnetic moment in the fitting parameters.
- Corrected two mistakes in the capacitive modeling, in particular for single-gated samples.

The new data sets and modeling approaches add the following insights to the manuscript:

- The model accuracy across different samples has improved, also predicting the gate-dependent changes in the magnon frequencies of the new bilayer devices reasonably well.
- We observe a layer-number dependent decrease in the high magnon frequency on a terraced sample, while the lower magnon frequency is almost unaffected by thickness. This confirms the predicted trend of the layer-resolved macrospin model.
- The tunability of the magnon frequency decreases when going from a 2- to 4-layer area within the same sample. This agrees with the trend we previously observed across different flakes.
- In the bilayer sample data, the fitted interlayer exchange fields are larger compared to those of the thicker samples, possibly indicating an enhanced interlayer interaction in the bilayer limit.

While these additional experiments and modeling delayed our response, we nevertheless believe that the resulting changes significantly improve the manuscript. We hope that it now meets the standard of *Nature Communications*.

In the following answer, the reviewers' questions are shown in blue, our answers in black, and changes to the manuscript are highlighted in red. Please note that we tried to make our responses to each reviewer self-contained – so that there is some overlap between individual answers.

Reviewer #1 (Remarks to the Author):

This manuscript investigates the impact of electrostatic gating on the magnetic properties and magnon dynamics of thin-layer CrSBr systems. The authors use a layer-resolved macrospin model to interpret the evolution of magnon modes under dual-gating conditions, distinguishing between in-phase and out-of-phase magnon responses. The work is clearly written and generally well-structured, reflecting substantial experimental effort and thoughtful theoretical modeling. However, I have serious concerns about the data analysis and interpretation, particularly regarding the model's agreement with experimental results. These issues must be thoroughly addressed before the work can be considered for publication.

We thank the reviewer for the thorough feedback and will address the concerns about the data analysis and interpretation in the following reply.

1. In Fig. 1(c), the macrospin model fails to accurately capture the experimental trends of magnon frequencies (f_IP and f_OP) as a function of gate voltage (Vb = Vt), particularly below 2 V. This discrepancy indicates that the model may not sufficiently account for low-carrier-density regimes or

nonlinear effects in this voltage range. A more detailed discussion of the model's limitations and potential physical mechanisms behind this deviation would enhance the interpretation of the results.

We agree. To improve the fits of the macrospin model to the trilayer data, we:

- Identified and corrected an erroneous capacitance in the capacitive model leading to more accurate electron density *n* and perpendicular electric field F_z
- Included the anisotropy field H_b in the fitting parameters (instead of fixing it in the previous model iteration)
- Changed the dependency in Eq. (5) from a doping-dependent gyromagnetic ratio to a doping-dependent macrospin length: $M_{0,i} = 1 + \eta_M n_i$. This is discussed in more detail in the answer to the last question by reviewer 2.

The resulting changed plots are shown in Fig. R1, where (a) shows the doping dependence of f_{IP} and f_{OP} (points: experimental data, solid lines: macrospin model fits). We also show the altered fits for the complete gate-dependence of (b) f_{IP} and (c) f_{OP} (Fig. R1). The corresponding plots in Fig. 1 of the main manuscript were updated accordingly.

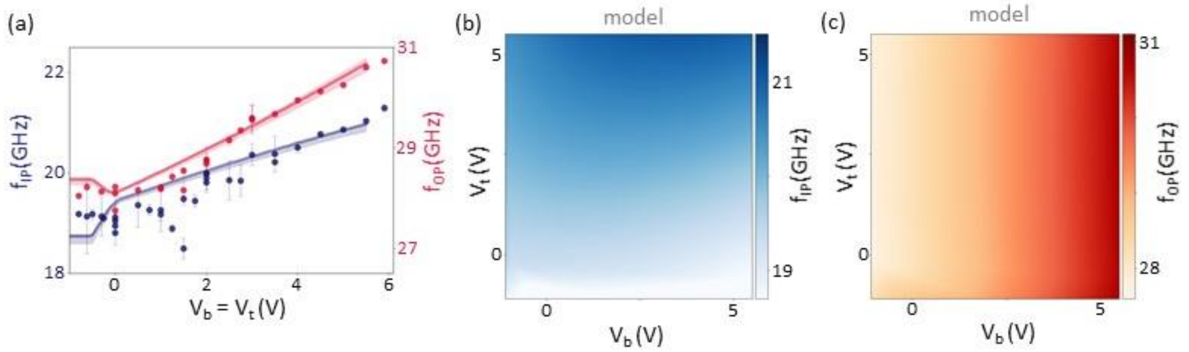

Fig. R1: Macrospin fitting of the magnon modes in the trilayer CrSBr device.

The overall agreement between data and fits has improved, in particular for f_{OP}. We note that there are still some discrepancies left in certain gate voltage regimes such as the delayed onset of magnon frequency shift with doping in Fig. R1 (a). This could indeed indicate that there is an additional threshold or non-linearity at low carrier densities and fields, which we do not take into account in the fit functions in Eq. (3)-(5). We discuss possible effects of doping and electric field on the magnetic properties of CrSBr in SI section 8:

"(1) By increasing the electron doping n, the magnitude of the macrospin can increase due to an increase in carriers with magnetic moment. The Coulomb repulsion parameter U can also increase with n, possibly influencing the exchange constants according to the Kugel-Khomskii model. (2) When doping with electrons, e_g levels will be populated in addition to t_{2g} ones. This can change the orbitals involved in the hopping between sites, again affecting the (anti-)ferromagnetic exchange coupling parameters. Additionally, the different orbital shapes can influence the anisotropy of the material. (3) A perpendicular electric field between layers can shift the energy positons of bands associated with different orbitals in neighboring layers with respect to each other, again influencing the (anti)ferromagnetic exchange coupling parameter. (4) Gates can influence orbital filling especially in the outer layers due to the resulting electric field, as electrons shift further/closer to the gate, shuffling them into $d_{x^2-y^2}$ or d_{z^2} orbitals, respectively. This is commonly called voltage-induced magnetic anisotropy."

This complex picture makes it very difficult to predict the gate-dependence of the magnetic parameters of the sample in full detail. Additionally, we note that the accuracy of the capacitive modeling (and in turn the fits to the macrospin model) is limited by the information we gather from the gate-dependent photoluminescence (PL) measurements. In particular, we fix the built-in field F_0 in the model so that the doping onset in the model corresponds to the drop in exciton-to-trion ratio in PL (SI section 4). Inaccuracies in the estimation of F_0 potentially affect the threshold voltage of the magnon frequency shifts, leading to an offset between the experimental and theoretically predicted value. We have added an outlook on these limitations in the discussion of the manuscript: "The accuracy of the layer-resolved macrospin model is limited by several simplifications, mainly concerning the estimation of carrier density and electric field from PL measurements and the focus on the dominating linear dependencies of the magnetic sample parameters. In the future, we believe that experiments using a variable external magnetic field on CrSBr and other material systems, as well as studying higher-momentum magnons can further improve our understanding of gate-dependent magnonic phenomena and expand our minimal model."

The goal of our work is to put forward the minimal model capturing the observed experimental trends quantitatively. We therefore fit the experimental data using the simplest linear approximation. To this end, we demonstrate that when the model is simplified even further, for example by removing the field dependencies, the model fails to capture the experimental data. This argument, below, is added to the SI section 9.

"The most intuitive consequences of an increased electron density in the sample are an increase of the layer magnetization as well as an increased interlayer exchange interaction. We therefore start by fitting the trilayer data only considering doping-dependent linear effects, explicitly

- $M_{0,i} \propto n_i$,
- $H_{A,i} \propto n_i$ *and*
- $H_{E,ij} \propto n_i + n_j$.

We show the difference between experimental (f_{ex}) and fitted (f_{mod}) magnon frequencies in Fig. R2 (a) (top: $f_{IP,ex} - f_{IP,mod}$, bottom: $f_{OP,ex} - f_{OP,mod}$). In the resulting Voronoi plots, white/faint colors correspond to small differences between the fit and the experimental plot, while bright red/blue signify larger deviations. Unsurprisingly, the resulting fits fail to capture any field-dependent changes of the magnon frequencies, seen by large deviations in the residual frequencies for $V_b \neq V_t$. From this, we conclude that at least one of the magnetic properties should be dependent on the perpendicular electric field F_z.

Therefore, we choose an electric field dependence of the interlayer exchange interaction in the fits, i.e.

- $M_{0,i} \propto n_i$,
- $H_{A,i} \propto n_i$ *and*
- $H_{E,ij} \propto F_{z,ij}$

In Fig. R2 (b). To avoid overfitting, we neglect the doping dependence of H_E. This model indeed fits the gate dependence of the f_{OP} mode better, visible in the more homogeneous and smaller deviations in the bottom plot of Fig. R2 (b). However, it still does not capture the field dependence of f_{IP}. This leads us to believe that in order to fit the field-dependence of both modes, we need to include two competing parameters $\propto F_{z,ij}$.

We therefore include a second field-dependence, choosing

- $M_{0,i} \propto n_i$,
- $H_{E,ij} \propto F_{z,ij}$ *and*
- $H_{A,i} \propto F_{z,i,i+1} + F_{z,i-1,i}$.

This captures the gate-dependencies of both f_{IP} and f_{OP} reasonably well (Fig. R2 (c)). The average difference between the modeled and experimental f_{OP} is < 0.2 GHz. The largest deviations in the residual frequency can be seen in the low carrier density regime around $V_b = V_t = 0$–2 V. This is due to the delayed onset of the frequency shifts also visible in Fig. 1c of the main manuscript. For f_{IP}, we can see that at high absolute field values F_z the fits deviate the most from the experimental behavior (up to 0.7 GHz), possibly indicating an additional phenomenon taking effect at those gate voltages. However, we also note that in the measurements on the trilayer device, the lower frequency mode had a weaker signature in the FFT, leading to a larger error when extracting its position. Overall, the average difference between the experimental and modeled f_{IP} is < 0.3 GHz.

Lastly, we examine whether including an additional doping dependence of the exchange interaction improves the fits further using

- $M_{0,i} \propto n_i$,
- $H_{E,ij} \propto n_i + n_j$,
- $H_{E,ij} \propto F_{z,ij}$ *and*
- $H_{A,i} \propto F_{z,i,i+1} + F_{z,i-1,i}$

In Fig. R2 (d). We see no significant improvements in the fits compared to those in Fig. R2 (c). We therefore neglect any dependencies beyond those shown in Fig. R2 (c) to avoid high errors of the fit parameters resulting from overfitting."

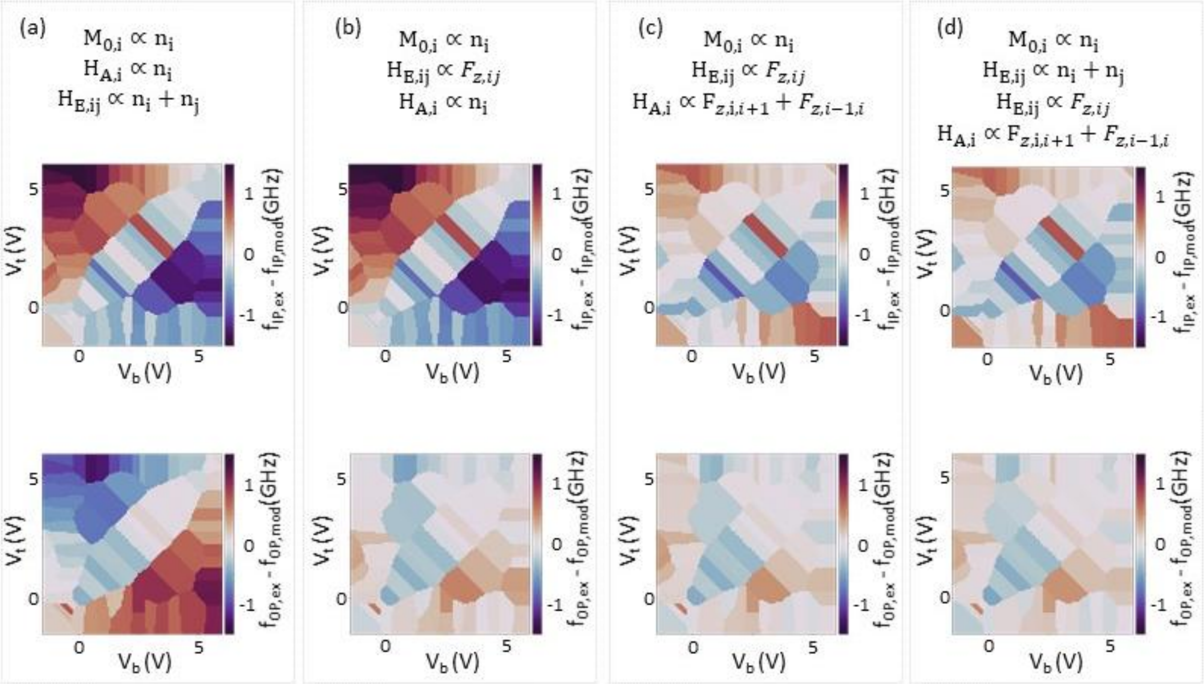

Fig. R2: Difference between fitted (f_{mod}) and experimental (f_{ex}) gate-dependent magnon frequencies in the trilayer CrSBr sample for different model dependencies on n and F. (a) to (d) refer to different fitting models described in the text (fit dependencies at the top); the color scale is the deviation between the fitting model and the data. The top row shows f_{IP}, the bottom row f_{OP}.

2. Both Fig. 1(c) and Fig. 2(d) show a crossing point of the magnon modes and carrier densities around 2 V. Is this a coincidence, or does it imply a critical carrier density distribution between the top (n_t) and bottom (n_b) layers? This aspect deserves explicit discussion in the manuscript. If this behavior arises from a specific balance in layer-resolved carrier densities, the authors should clarify its implications for the magnon spectrum.

The crossing visible in Fig. 1(c) is not a real effect, but a result of plotting the two magnon frequencies together in one plot with different y-axes. To avoid this confusion, we have changed the y-axes ranges (see Fig. R1 (a) above).

3. In Fig. 4, the agreement between experimental data and model predictions—particularly for f_OP in the 5-layer system—is poor. The data indicate minimal tunability of f_OP with bottom gate voltage, contradicting model expectations. The authors should clarify whether interlayer interactions are significantly weakened or screened in thicker samples, and how this affects the validity of their model assumptions.

We have made several changes to the macrospin fits to describe our experiments better (more details in our answer to point 1 of the same reviewer). In addition, for the 5-layer device, we have found and corrected a mistake in the capacitive modeling. In the gate-dependent photoluminescence measurements on this sample, we had previously noticed that the top gate was not properly working – meaning, while voltage was applied to the gold contacted graphene gate, it did not induce a potential shift in the measured area of the device. One possible reason is a tear in the graphene flake that was not visible during the assembly of the device. In the previous simulations, we nevertheless calculated the charge carrier density in the top layer by solving

$$0 = \left(V_t + V_{t,0}\right) - E_{F,t} - \frac{en_t}{C_t} - \frac{C_{CrSBr}}{C_t}\left(E_{F,t} - E_{F,m}\right)$$

and setting $V_t = 0$. However, in the case of a non-functioning top gate this is not correct, as the Fermi level in the top gate is not fixed by the chemical potential in the gate anymore. Instead, we now use the formula derived using no top gate:

$$0 = -en_t - C_{CrSBr}\left(E_{F,t} - E_{F,m}\right).$$

The updated modeling for the 5-layer sample is shown together with the experimental data points in Fig. R3.

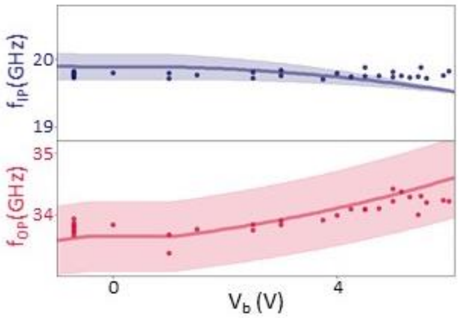

Fig. R3: Corrected gate-dependent frequencies of OP and IP magnons within the macrospin model (solid lines) and corresponding experimental data (points) for the 5-layer CrSBr device.

We find a clear improvement of the model agreement with respect to the original manuscript, in particular at low gate voltages. The shaded error regions are estimated by fitting the 5-layer data while varying the *n*- and *F*-dependencies found for the trilayer data in their error interval (Table R1). An independent precise fitting of the 5-layer sample data is not possible due to the incomplete field dependence.

To address the concern regarding the thickness dependence of the interlayer interactions, and encouraged by further comments by reviewer 3, we have measured two additional gated bilayer CrSBr samples. The improved fitting parameters for all samples, summarized in the table below, do imply the trend suggested by the reviewer – the highest interlayer exchange field H_{E0} is seen in bilayer samples. However, it is hard to compare quantitatively due to a general variation of the interlayer exchange and anisotropy fields between samples, even for samples with similar thicknesses (see, e.g. H_{E0} for the two new bilayer samples). This makes it difficult to say with certainty whether there are thickness-dependent trends in the interlayer interactions. Table 1 of the main manuscript has been updated with the new fitting parameters and samples.

	H_{E0} (T)	H_{a0}(T)	H_b (T)	ν_a (Tnm/V)	ν_E (Tnm/V)	η_M (10^{-12} cm^2)
**3L (original text, dual-gated)**	0.163 (3)	1.15 (20)	1.48 (20)	-0.86 (10)	-0.85 (10)	0.0073 (1)
**5L (original text, single-gated)**	0.295 (5)	0.96 (10)	1.27 (1)			
**8L (original text, dual-gated)**	0.25 (1)	0.91 (1)	1.295 (15)			
**2L (new, single-gated)**	0.50 (1)	0.98 (2)	1.22 (1)			
**2L (new, dual-gated)**	0.67 (3)	0.92 (1)	1.16 (1)			

Table R1: Updated fitting parameters for all CrSBr samples. The gyromagnetic ratio γ = 155 GHz T^{-1} is fixed in the model. H_{E0}, H_{a0} and H_b are the internal magnetic fields and are fitted for each sample individually. The fit parameters ν_E and ν_a describe the electric-field dependencies of the exchange and anisotropy fields, respectively, and η_M the doping-dependence of the layer macrospin. They were fitted using the field-dependent magnon frequencies of the trilayer CrSBr. For the 2-, 5- and 8-layer CrSBr devices, we fit the experimental frequencies to the macrospin model with ν_a, ν_E and η_M fixed to the trilayer values in the top row.

4. The model employs a linear dependence (Eqs. 3–5) to describe gate-induced changes in magnetic parameters, but the physical justification for this assumption is unclear. According to Table I, the magnitude of these changes—such as an interlayer exchange shift of ~1 T—appears unreasonably large compared to the zero-field coupling (H_E0 ~0.2 T), suggesting the linear response regime has been exceeded. A detailed discussion on the validity of this approximation is essential, including whether nonlinear or saturation effects should be taken into account.

We note that the field-dependent changes in table 1 of the main manuscript (see Table R1) show the fit parameters, i.e. the slopes of exchange or anisotropy field versus perpendicular electric field. The value of, e.g., $\nu_{\mathrm{E}} = 0.85$ Tnm/V therefore corresponds to a maximum change in the exchange field of ~0.1 T (and not 1 T), since we only reach electric fields of ~0.1 V/nm (SI section 6, 7). This change is similar in magnitude to what has been reached in bulk CrSBr under strain [*Diederich, G. M. et al., Nature Nano 18:1, 1 (2022)*].
It is still true that the changes from the initial interlayer exchange field of ~0.16 T are significant. However, we avoid making claims of saturation or non-linear effects due to limitations in determining *n* and *F* as well as the sheer number of possible physical effects governing the magnon dynamics (as discussed in the answer to question 1 of the same reviewer). We also note that in Fig. 4(c) in [*Zhang, X.-X. et al., Nature Materials, 19:8, 838 (2020)*], the gate-dependent change in exchange interaction of CrI_3 – being of a similar order of magnitude – follows a linear trend.

Reviewer #2 (Remarks to the Author):

The manuscript entitled "Tunable magnons in a dual-gated 2D antiferromagnet" reports an investigation of the magnetization dynamics eigenmodes (magnons) in a gated trilayer of the prototypical van der Waals antiferromagnetic semiconductor – CrSBr. The experiments employ optically detected excitonic and trionic resonances to probe the magnetic oscillations as well as the effect of the gate voltages. The authors find that the magnetic eigenmode frequencies depend on the voltages applied to the top and bottom gates. This dependence is most pronounced for one particular magnon mode in the trilayer and becomes weak for thicker samples. The authors employ a theoretical model that treats the typical magnetic characteristics of the material, such as anisotropy, to be layer dependent and as fitting parameters.

The observed tunability of the magnon modes with respect to the gate voltage is intriguing and a useful property of such van der Waals magnets. Its observation in the air stable material CrSBr is especially exciting and valuable. Hence, I find the main message and goal of the article to fit the scope of Nature Communications. However, at present, I am having trouble digesting some of the data interpretation and the corresponding conclusions drawn by comparing to the theoretical model. I find it important to clarify the following scientific issues before I can make a clear recommendation.

We thank the reviewer for the very helpful feedback and discuss the detailed changes to address concerns about the data interpretation and model below.

1. The authors have employed oscillations in the transient reflectivity as a direct signature and measure of the magnon modes. A similar attribution has indeed been done in previous literature, e.g., Ref. [21]. However, this attribution stems from a specific interaction between the magnon and the exciton in question, and requires the CrSBr to be in a canted magnetic ground state. I guess that this is why the authors also need to subject their sample to a permanent magnet and applied magnetic field, so that a canted magnetic state is achieved in equilibrium.

The manuscript does not seem to clarify how their transient reflectivity maps to magnon modes. What is the nature of the excitons and trions that allows this relation between the reflectivity and magnons?

The relation between reflectivity and magnons lies in the modulation of the excitonic binding energy as a function of the magnetic state, which is a unique feature of CrSBr [*Wilson, N. P. et al., Nature Materials 20:12, 1657 (2021), Brennan, N.J. et al., ACS Physical Chemistry Au, 4:4, 322 (2024)*]. In its fully antiferromagnetic (AFM) state, the bright excitons in neighboring layers of CrSBr are decoupled due to their opposite spin directions. In the ferromagnetic (FM) state, the electron and hole orbitals of the excitons can undergo interlayer hybridization, effectively increasing the size of the exciton wavefunction. This leads to a redshift of the excitonic resonance when switching from the antiferromagnetic state to the canted or ferromagnetic state [*Wilson, N. P. et al., Nature Materials 20:12, 1657 (2021), Brennan, N.J. et al., ACS Physical Chemistry Au, 4:4, 322 (2024), Dirnberger, F. et al., Nature 620:7974, 533 (2023)*]. The magnitude of the energy shift ΔE scales with cos(ϑ), where ϑ is the angle between the macrospins of neighboring layers. A transient change away from the purely AFM state due to magnons changes the excitonic binding energy in a time-dependent manner. The

resulting oscillatory shift of the excitonic resonance in the reflectivity spectra renders magnons visible in tr-reflectivity measurements with a fixed probe wavelength. We have added a condensed version of this explanation to the manuscript: "The excitons in CrSBr around 1.37 eV exhibit a strong coupling to the magnetic order of the sample: Depending on the orientation of the macrospins in neighboring layers, the interlayer character and degree of localization of the excitons differs [40–42]. This results in a change of their binding energy, and accordingly their energetic resonance position in photoluminescence and reflectivity spectra [22, 23, 25]. Oscillations in the magnetic order can therefore be seen as oscillations in the tr-reflectivity signal of the sample [21, 26, 28]."

Generally, the exciton-magnon coupling in CrSBr does not require a canted magnetization of the CrSBr layers. However, the sensitivity of the tr-reflectivity measurement to different magnon modes depends on the direction of canting of the magnetization: for $H_0 \,||\, \hat{a}$, only f_{OP} is visible in tr-reflectivity, and for $H_0 \,||\, \hat{c}$, only f_{IP} is visible. For an external field with contributions along both axes, both magnon modes can be seen [*Sun, Y. et al., Nature Physics 2024, 1-7 (2024)*]. An external magnetic field also improves the amplitude of the oscillations in tr-reflectivity [*Bae, Y. J. et al., Nature 609:7926, 282 (2022)*].

The exact nature of the excitons around 1.37 eV has been the topic of extended research. In a recent study on excitonic lifetimes in CrSBr, it has been proposed that the emission stems from quasi one-dimensional excitons which are strongly localized in the layers, e.g. due to defects [*Kalitukha, I. v. et al. arxiv:2601.05413 (2026)*]. If this confinement is reduced along the c-axis due to a change in magnetic order, this is detectable as a shift in the excitonic resonance in reflectivity measurements. In another recent publication [*Śmiertka, M. et al., Nature Comms, doi:10.1038/s41467-026-68482-5 (2026)*], the excitons as 1.37 eV behave as Frenkel-like excitons, i.e. they are localized. In [*Liebich, M. et al., Nature Materials 24:3, 384 (2025)*], the authors demonstrated that the excitons can be tuned from monolayer-like states in the AFM phase to interlayer-delocalized states in the FM phase. We therefore conclude that the excitons in CrSBr around 1.37 eV are intralayer excitons with a partially interlayer character, depending on the magnitude of the external magnetic field.

2. Continuing along the same direction as above, the excitonic modes that have been used to detect coherent magnons (such as in Ref. [21]) obtain a varying degree of interlayer character with the magnetic canting (see Ref. [22], for example). This also affects their oscillator strength as discussed in PRB 111, 075107 (2025), for example. Both of these features, varying oscillator strength and interlayer character, have not been accounted for in making inferences in the present manuscript. Instead the authors seem to treat their excitons and trions as intralayer, whose detectability is assumed to be related only to the charge doping. Is this justifiable?

We thank the reviewer for pointing this out. We agree that the magnetic state of CrSBr affects the localization volume, energy position and oscillator strength of the excitons. However, in the current work, we are only focused on the frequency (rather than amplitude) of the oscillations in the tr-reflectivity. In that sense, whether the magnons affect the position or the width of the resonance, this change should happen at the same frequency regardless.
When inferring the doping level of individual layers from photoluminescence, the interlayer character of the excitons is indeed important. We treat the excitons and trions as intralayer, since our applied external magnetic field of ~0.1 Tesla only leads to a small canting angle of the macrospins (around 3 degree, comparing to Fig. 1(d) in [*Heißenbüttel, M.-C. et al., PRB 111, 075107 (2025)*]). As discussed in the answer to the previous question, the excitons will therefore only have a small interlayer contribution. We assume that the same is true for trions at small external fields, while noting that it has been shown in magneto-optical spectroscopy that intra-interlayer trion coupling cannot be completely disregarded when explaining the behavior of trions in magnetic fields [*Tabataba-Vakili, F. et al., Nature Comms 15:1, 4735 (2024)*]. We expect the effect of the magnons

themselves on the interlayer character and oscillator strength of the excitons to be very small, as they represent only minor deviations from the initial canting angle given by the external field. We now make this assumption more transparent in the manuscript: "We quantitatively link I_B and $I_{B'}$ to the layer-resolved electron densities ni and electric fields $F_{z,i\,j}$ (where i, j = top, middle or bottom layer) using a simple electrostatic model [50, 51]. For this, we assume that the interlayer character of the excitons is sufficiently small because the weak applied external magnetic field results in a small canting angle of the in-plane magnetization [42]."

3. In Fig. 1(c), the comparison between theory and experiment is not good enough to draw conclusions of agreement. The authors should examine and comment on this point.

We agree. To improve the fits of the macrospin model to the trilayer data, we:

- Identified and corrected an erroneous capacitance in the capacitive model leading to more accurate electron density *n* and perpendicular electric field F_z
- Included the anisotropy field H_b in the fitting parameters (instead of fixing it in the previous model iteration)
- Changed the dependency in Eq. (5) from a doping-dependent gyromagnetic ratio to a doping-dependent macrospin length: $M_{0,i} = 1 + \eta_M n_i$. This is discussed in more detail in the answer to the last question by the same reviewer.

The resulting changed plots are shown in Fig. R4, where (a) shows the doping dependence of f_{IP} and f_{OP} (points: experimental data, solid lines: macrospin model fits). We also show the altered fits for the complete gate-dependence of (b) f_{IP} and (c) f_{OP} (Fig. R4). The corresponding plots in Fig. 1 of the main manuscript were updated accordingly.

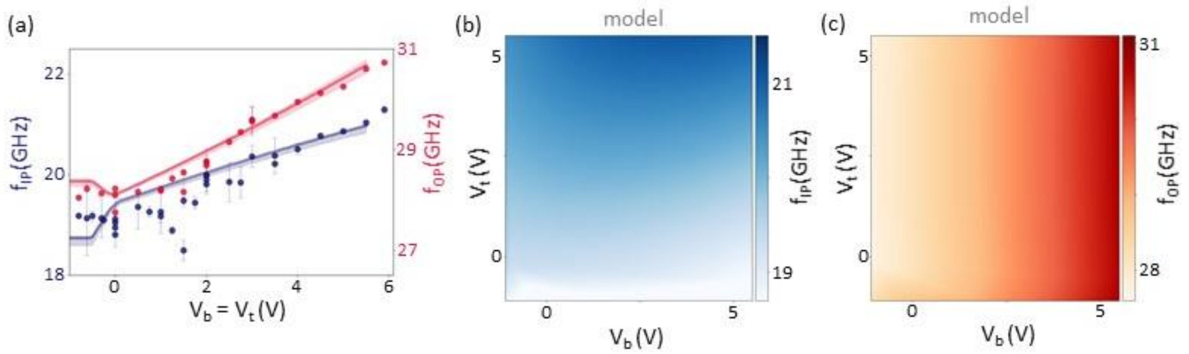

Fig. R4: Macrospin fitting of the magnon modes in the trilayer CrSBr device.

The overall agreement between data and fits has improved, in particular for f_{OP}. We note that there are still some discrepancies left in certain gate voltage regimes such as the delayed onset of magnon frequency shift with doping in Fig. R1 (a). This could indeed indicate that there is an additional threshold or non-linearity at low carrier densities and fields, which we do not take into account in the fit functions in Eq. (3)-(5). We discuss possible effects of doping and electric field on the magnetic properties of CrSBr in SI section 8:

"(1) By increasing the electron doping n, the magnitude of the macrospin can increase due to an increase in carriers with magnetic moment. The Coulomb repulsion parameter U can also increase with n, possibly influencing the exchange constants according to the Kugel-Khomskii model. (2) When doping with electrons, e_g levels will be populated in addition to t_{2g} ones. This can change the orbitals involved in the hopping between sites, again affecting the (anti-)ferromagnetic exchange coupling parameters. Additionally, the different orbital shapes can influence the anisotropy of the material. (3)

A perpendicular electric field between layers can shift the energy positons of bands associated with different orbitals in neighboring layers with respect to each other, again influencing the (anti)ferromagnetic exchange coupling parameter. (4) Gates can influence orbital filling especially in the outer layers due to the resulting electric field, as electrons shift further/closer to the gate, shuffling them into $d_{x^2-y^2}$ or d_{z^2} orbitals, respectively. This is commonly called voltage-induced magnetic anisotropy."

This complex picture makes it very difficult to predict the gate-dependence of the magnetic parameters of the sample in full detail. Additionally, we note that the accuracy of the capacitive modeling (and in turn the fits to the macrospin model) is limited by the information we gather from the gate-dependent photoluminescence (PL) measurements. In particular, we fix the built-in field F_0 in the model so that the doping onset in the model corresponds to the drop in exciton-to-trion ratio in PL (SI section 4). Inaccuracies in the estimation of F_0 potentially affect the threshold voltage of the magnon frequency shifts, leading to an offset between the experimental and theoretically predicted value. We have added an outlook on these limitations in the discussion of the manuscript: "The accuracy of the layer-resolved macrospin model is limited by several simplifications, mainly concerning the estimation of carrier density and electric field from PL measurements and the focus on the dominating linear dependencies of the magnetic sample parameters. In the future, we believe that experiments using a variable external magnetic field on CrSBr and other material systems, as well as studying higher-momentum magnons can further improve our understanding of gate-dependent magnonic phenomena and expand our minimal model."

In this work, we would like to propose the absolute minimal dependencies needed to describe the observed experimental trends quantitatively, which should give us access to the dominant effects.

4. The seemingly good agreement between figures 1(d) and (f) [1(e) and (g)] also relies on several ad hoc fitting parameters for each layer, and as such should be considered more carefully.

Our goal is to develop a minimal model that on one hand describes the entirety of our data and on the other hand is physically justifiable. The logic of our argument (now added to the SI section 9) is found below.

"The most intuitive consequences of an increased electron density in the sample are an increase of the layer magnetization as well as an increased interlayer exchange interaction. We therefore start by fitting the trilayer data only considering doping-dependent linear effects, explicitly

- $M_{0,i} \propto n_i$,
- $H_{A,i} \propto n_i$ *and*
- $H_{E,ij} \propto n_i + n_j$.

We show the difference between experimental (f_{ex}) and fitted (f_{mod}) magnon frequencies in Fig. R2 (a) (top: $f_{\mathrm{IP,ex}} - f_{\mathrm{IP,mod}}$, bottom: $f_{\mathrm{OP,ex}} - f_{\mathrm{OP,mod}}$). In the resulting Voronoi plots, white/faint colors correspond to small differences between the fit and the experimental plot, while bright red/blue signify larger deviations. Unsurprisingly, the resulting fits fail to capture any field-dependent changes of the magnon frequencies, seen by large deviations in the residual frequencies for $V_b \neq V_t$. From this, we conclude that at least one of the magnetic properties should be dependent on the perpendicular electric field F_z.

Therefore, we choose an electric field dependence of the interlayer exchange interaction in the fits, i.e.

- $M_{0,i} \propto n_i$,
- $H_{A,i} \propto n_i$ *and*
- $H_{E,ij} \propto F_{z,ij}$

In Fig. R2 (b). To avoid overfitting, we neglect the doping dependence of H_E. This model indeed fits the gate dependence of the f_{OP} mode better, visible in the more homogeneous and smaller deviations in the bottom plot of Fig. R2 (b). However, it still does not capture the field dependence of f_{IP}. This leads us to believe that in order to fit the field-dependence of both modes, we need to include two competing parameters $\propto F_{z,ij}$.

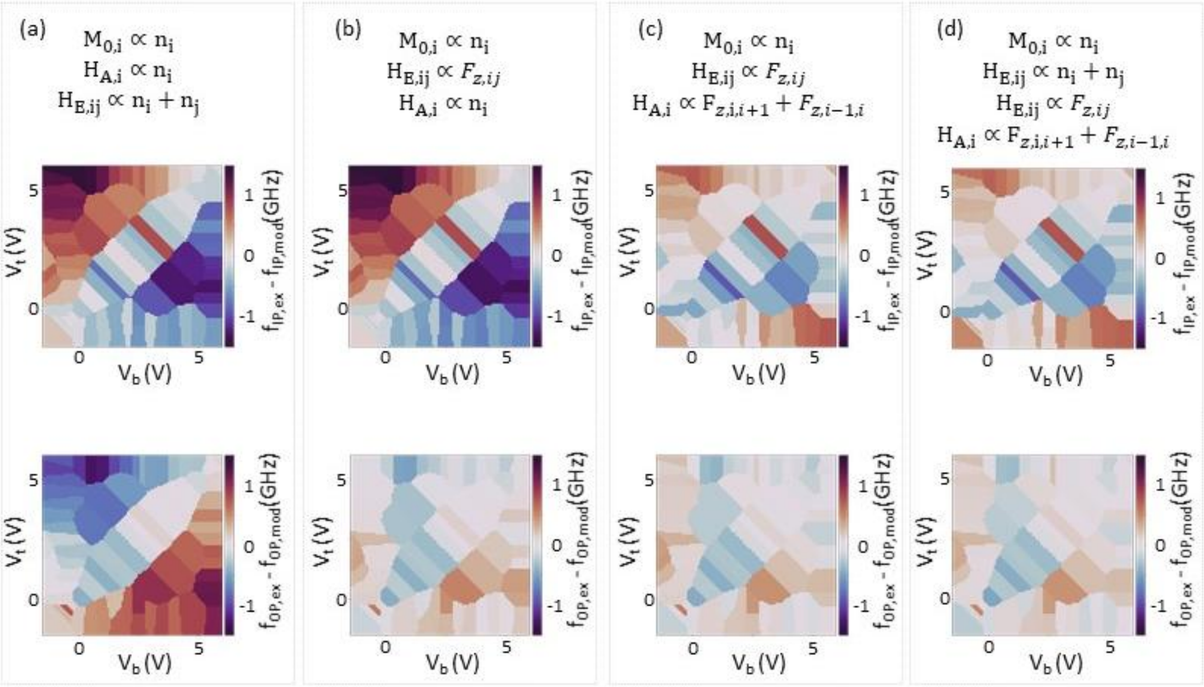

Fig. R5: Difference between fitted (f_{mod}) and experimental (f_{ex}) gate-dependent magnon frequencies in the trilayer CrSBr sample for different model dependencies on n and F. (a) to (d) refer to different fitting models described in the text (fit dependencies at the top); the color scale is the deviation between the fitting model and the data. The top row shows f_{IP}, the bottom row f_{OP}.

We therefore include a second field-dependence, choosing

- $M_{0,i} \propto n_i$,
- $H_{E,ij} \propto F_{z,ij}$ *and*
- $H_{A,i} \propto F_{z,i,i+1} + F_{z,i-1,i}$.

This captures the gate-dependencies of both f_{IP} and f_{OP} reasonably well (Fig. R2 (c)). The average difference between the modeled and experimental f_{OP} is < 0.2 GHz. The largest deviations in the residual frequency can be seen in the low carrier density regime around V_b = V_t = 0–2 V. This is due to the delayed onset of the frequency shifts also visible in Fig. 1c of the main manuscript. For f_{IP}, we can see that at high absolute field values F_z the fits deviate the most from the experimental behavior (up to 0.7 GHz), possibly indicating an additional phenomenon taking effect at those gate voltages. However, we also note that in the measurements on the trilayer device, the lower frequency mode had a weaker signature in the FFT, leading to a larger error when extracting its position. Overall, the average difference between the experimental and modeled f_{IP} is < 0.3 GHz.

Lastly, we examine whether including an additional doping dependence of the exchange interaction improves the fits further using

- $M_{0,i} \propto n_i$,
- $H_{E,ij} \propto n_i + n_j$,
- $H_{E,ij} \propto F_{z,ij}$ *and*
- $H_{A,i} \propto F_{z,i,i+1} + F_{z,i-1,i}$

In Fig. R2 (d). We see no significant improvements in the fits compared to those in Fig. R2 (c). We therefore neglect any dependencies beyond those shown in Fig. R2 (c) to avoid high errors of the fit parameters resulting from overfitting."

First, from symmetry, we would expect that a trilayer sandwiched between two symmetric gates should respond in the same manner to voltages applied to the top and bottom gates. The experiments however find a strong asymmetry in how the magnon modes respond to the voltages in the two gates, as per Fig. 1(d) and (e). This requires a more detailed justification before going to a phenomenological model.

We hypothesize that part of the asymmetric response of the magnon modes to the two gates is a result of the broken inversion symmetry of the sample due to, e.g. different hBN thicknesses or polymer residues on the top and bottom of the stack. In the gate-dependent PL measurements on the trilayer CrSBr sample we find an asymmetric response to V_b and V_t, which we attribute to a built-in electric field $F_0 < 0$ (SI section 3). As a result, the overall gate-dependence of the field F_z in the trilayer CrSBr is shifted with respect to $V_b = V_t$ (Fig. R6). We believe that this is one factor of the asymmetry seen in the gate-dependence of the magnon frequencies (as both frequencies are asymmetric with respect to $V_b = V_t$).

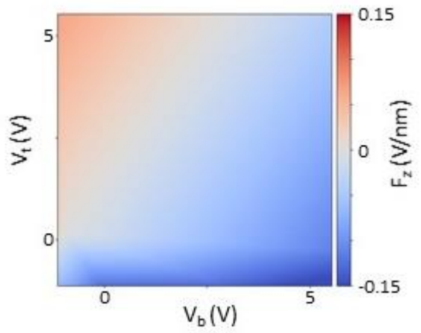

Fig. R6: Modeled gate-dependence of the electric field F_z in the trilayer CrSBr sample.

However, this does not explain the directional field-dependence of f_{IP} and f_{OP}. Indeed, we intuitively expect the effects of an electric field to be symmetric with respect to its sign, i.e. $\Delta f(F_z) = \Delta f(-F_z) = \Delta f(|F_z|)$. However, we observe a dependence of both modes on the polarity of the perpendicular electric field and therefore use the dependencies $H_E \propto F_z$ and $H_a \propto F_z$ to accurately model the frequencies with the macrospin model.

It has been predicted that the presence of a substrate can lead to a dependence of magnon energies on the direction of F_z, e.g. in [*Marmodoro, A. et al., PRB, 105, 174411 (2022)*]. A dependence of the voltage-induced magnetic anisotropy on F_z has already been demonstrated, for example in few-layer iron [*Maruyama, T. et al, Nature Nano 4:3, 158 (2009)*] and FePd [*Bonell, F. et al., APL 98:23 (2011)*].

A similar asymmetry of the magnonic response has also been observed in the gate-dependence of the ferromagnetic resonance of the 2D magnet $Cr_2Ge_2Te_6$ [*Hendriks, F. et al., Nature Comms, 15:1, 1 (2024)*]. In this study, the magnon frequency increases when applying a field in one direction, and decreases when flipping the polarity. This agrees with our observation of the gate dependence of f_{IP} (the "ferromagnetic-like mode"). We therefore hypothesize that this directional effect is inherent to different supported dual-gated 2D magnetic materials.

However, we do not know the ultimate reason for the dependence of system's parameters on the direction of F_z, which we now make clearer in the manuscript: "The modeled frequency maps shown in Fig. 1f, g also reproduce all basic trends of the data reasonably well, in particular the opposing V_t- and V_b-sensitivity of the two magnon modes. To model this behavior, it is necessary to include the sign of the electric field in Eqs. (3) and (4) – which is surprising considering the symmetrical nature of the device stack."

This asymmetry could also be a consequence of the excitons being interlayer.

We note that in time-resolved measurements, we only use the excitons as proxies for sensing spin waves. In particular, we measure the response of both X_B and $X_{B'}$ excitons to the magnons as the two resonances are too close in energy to discern them with the probe light. This means that we always measure the collective response of the excitons in the whole sample. We are not able, for example, to detect the difference in macrospin oscillations closer to the surface or inside the bulk. In that sense, the intra- or interlayer character of the excitons does not affect which magnons we measure in the experiment.

To further examine the effect of the interlayer nature of excitons (and their potential contribution to the asymmetry), motivated by reviewer's comment, we probed the interlayer character of the excitons by examining their response to a perpendicular electric field in PL measurements. For interlayer excitons we would expect, e.g., an energy shift of the PL emission depending on F_z due to Stark splitting. We do not see such an effect in our electric field-dependent PL measurements: In the achievable field regime in which the excitons are visible, their energy shift is negligible, as we would expect for intralayer excitons (fitted energy position of the X_B (blue) and $X_{B'}$ (green) excitons for fixed V_t = -0.65 V and varying V_b in Fig. R7).

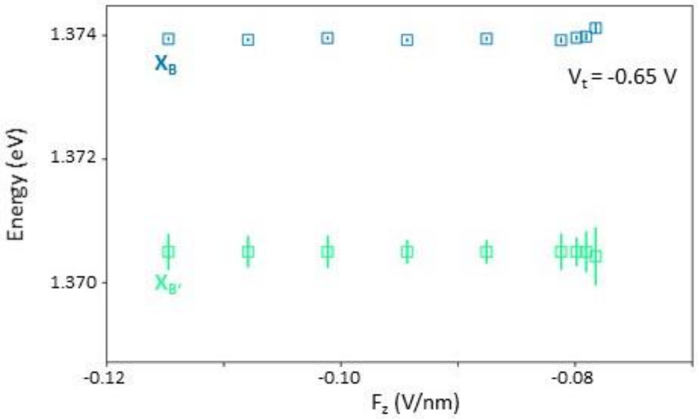

Fig. R7: Field-dependence of the energy position of the X_B and $X_{B'}$ excitons for a fixed top gate voltage and varying bottom gate voltage in 3L CrSBr.

5. In Fig. 3(b), the authors find three magnon modes from their numerical theoretical modeling. However, the experiment only finds two modes, consistent with the analytic model.

The difference in the number of eigenmodes in the numerical model is a result of explicitly including the macrospins of all layers. By including all N macrospins in an N-layered sample, we arrive at $2N$ solutions (N frequencies with negative and positive sign each), as compared to only 2 solutions in the simplest analytical model, which implicitly assumes that the macrospin of every second layer is the same. This is to say that in the numerical model, we allow a phase difference between the macrospins across layers, i.e. a finite momentum k_z of the magnons. As a result, the additional modes in the numerical model describe the magnon dispersion along k_z (when using sufficiently high N).

To confirm the validity of the numerical model, we can compare limiting cases in which it should align with the analytical one. For example, the solutions of the numerical model of the two branches for $f_{IP}(k_z = 0)$ and $f_{OP}(k_Z = 0)$ should be the same as the two frequencies from the analytical model. We show this agreement in Fig. R8 (blue stars: analytical results, black empty circles: numerical model for $N = 100$ with open boundary conditions).

Additionally, we can also calculate the analytical dispersion when including a phase difference between neighboring macrospins (calculation in SI section 5). This analytical dispersion (red solid line in Fig. R8) agrees with the numerical one, apart from the existence of edge modes. However, using periodic boundary conditions in the numerical model yields identical results to the analytical dispersion (blue circles Fig. R8). We conclude that the numerical and analytical model converge for thick samples. However, the numerical model allows us to include layer-resolved changes of the magnetic parameters of few-layered samples.

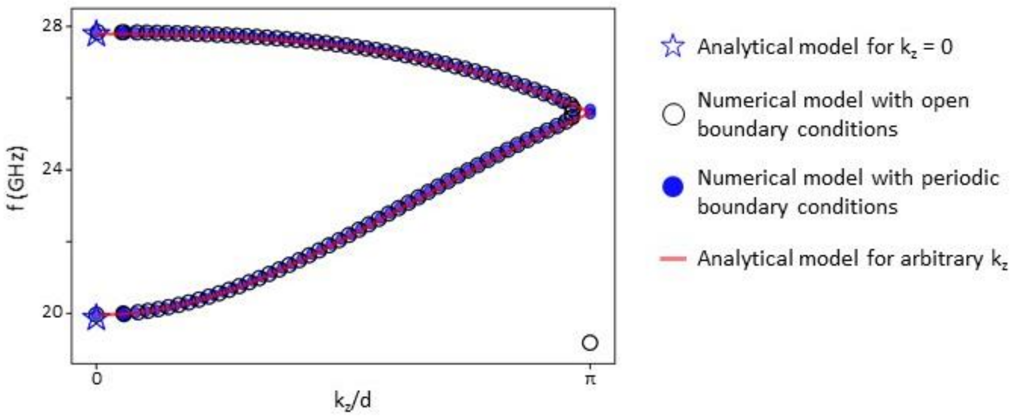

Fig. R8: Comparison of the magnon frequencies vs. wavevector sample thickness d obtained by the analytical and numerical modeling approaches discussed in the text.

The authors resolve this issue by disregarding the magnon mode with the middle frequency. Can this be justified on physical grounds? If not, can we rely on the numerical theoretical model which does not agree with the experiment about the number of magnon modes?

In bulk samples, it is easy to argue that a photoinduced excitation only couples to magnons with minimal k_z, as photons have zero momentum [*Bae, Y. J. et al., Nature 609:7926, 282 (2022)*], corresponding to $f_{IP}(k_z = 0)$ and $f_{OP}(k_Z = 0)$ in Fig. R8. In the case of thin samples, however, it is no longer intuitive to talk about k_z due to the reduced number of macrospins. We therefore need another way to estimate which modes are excited efficiently in an optical experiment. We propose

that the coupling for each eigenmode to a photoinduced excitation is proportional to the scalar product between that mode and an excitation-induced homogeneous change in $\vec{m}_{0,i}$ across layers.

We model two kinds of excitations for this estimation, specifically, in which all equilibrium macrospins $\vec{m}_{0,i}$ change in the same or alternating directions, resulting in new equilibrium positions $\vec{m}_{0,i}{}'$ after the excitation (top and bottom images in Fig. R9(a)). We then calculate the scalar product of the magnonic eigenmodes $\delta\vec{m}_i$ with these new equilibrium macrospins $\vec{m}_{0,i}{}'$. In the resulting graphs in Fig. R9(b,c), we show the layer-dependent eigenfrequencies color-coded by the magnitude of this scalar product (top and bottom graph correspond to different excitation types). Critically, we see that for high layer number *N* this scalar product is non-zero only for the lowest and highest non-edge modes (due to their low k_z). For low *N*, the edge mode spreads across the sample and gets efficiently excited.

In the trilayer, we find that the middle eigenmode couples the least efficiently to a photoinduced excitation. For an intuitive reasoning, we explicitly show the eigenmodes $\delta\vec{m}_i$ for the trilayer in Fig. R9(d) (top to bottom shows lowest to highest mode). We see that the highest and lowest modes represent a symmetric change from the equilibrium macrospins (grey and colored arrows in inset sketches of Fig. R9(d)), consistent with a homogeneous excitation across the sample. In the middle eigenmode, however, we can see that the change in the components $\delta m_{i,\mathrm{c}}$ is asymmetric across the sample and would require an excitation mechanism that points in opposite directions for the macrospins in the top and bottom layers, while leaving the middle one unaffected. We therefore assume that the middle mode does not efficiently couple to a photoinduced excitation and discard it for the analysis of our experimental data. To improve clarity on these points, we have extended the discussion in SI section 7.

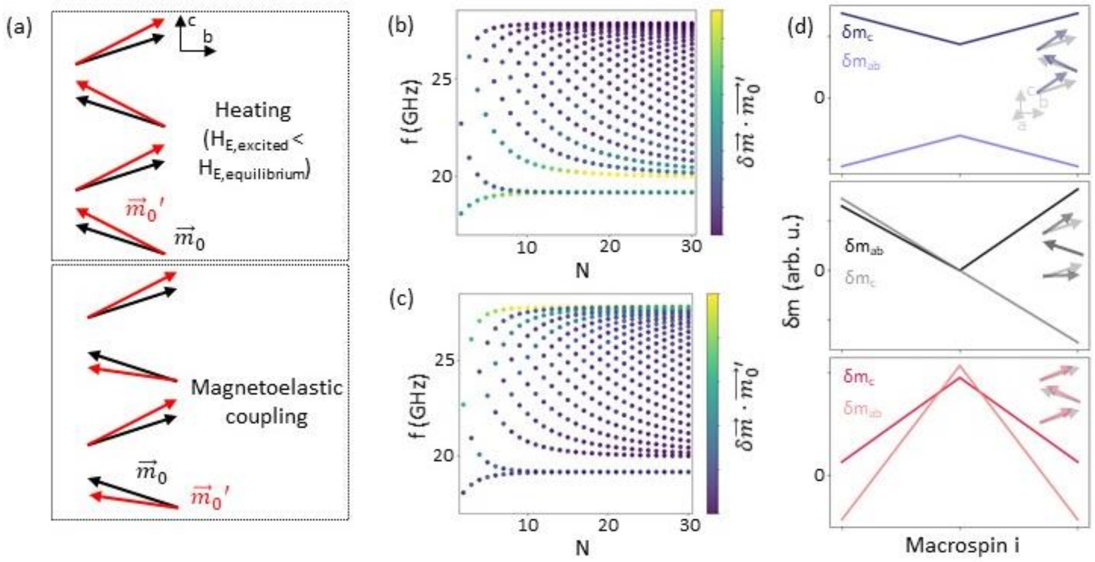

Fig. R9: Modeling of eigenmodes in multilayer CrSBr coupling to different type of zero-k_z excitation (top – symmetric, bottom – antisymmetric excitation). (d) shows the trilayer eigenmodes, specifically the out-of-plane component $\delta m_{i,\mathrm{c}}$ (dark colors) and the in-plane component $\delta m_{i,\mathrm{ab}}$ (transparent colors).

6. In assuming the phenomenological fitting equations (3) – (5), the authors argue the feasibility of the assumed dependencies on the charge density and electric field. This discussion is somewhat counterintuitive. For example, I would expect that the exchange interaction depends on the charge density which determines the screening of Coulomb interaction, as also briefly mentioned by the

authors. Instead, the authors assume the exchange to depend only on the electric field, and not the charge density.

We have discussed the choice of fitting parameters in more detail in our answer to point 4 of the same reviewer. While we expect a dependence of H_E on n, we find that including it in the macrospin fits yields minimal changes to fitting accuracy – so we did not include this effect to avoid overfitting. The F_z-dependence, on the other hand, is crucial to explain the gate asymmetry of the two modes. The extended reasoning was added to SI section 9.

At the same time, the assumed dependence of the gyromagnetic ratio on the charge density seems hard to justify. The authors state "the position of the chemical potential – controlled by n – affects the imbalance between the occupied spin-up and spin-down states, affecting the magnetic moment μ and in turn the gyromagnetic ratio γ of the corresponding macrospin". However, the imbalance between occupied states should affect only the magnetic moment, but not the gyromagnetic ratio which is the ratio between the magnetic moment and spin. The spin should also change with the changing magnetic moment to maintain the gyromagnetic ratio.

We thank the reviewer for pointing out this inaccuracy. Previously, we included a variable gyromagnetic ratio in our model to describe the doping-dependence of the macrospin. Instead, the n-dependent change in magnetic moment leads to a variation in the magnitude of the saturation magnetization of each layer. We have therefore altered the model to explicitly include the length of the layer-resolved macrospins $M_{0,i}$ (where i is the layer number) while fixing the gyromagnetic ratio. Previously, we had set $M_0 = 1$ in all layers for simplicity (i.e., normalized the macrospins).

Explicitly, this means that the expression for the energy of the system is now

$$E = -\vec{H}_0 \sum_i M_{0,i}\vec{m}_i + \sum_{<i,j>} H_{E_{ij}} M_{0,i} M_{0,j} \vec{m}_i \cdot \vec{m}_j - \frac{1}{2}\sum_i M_{0,i}^2 H_{a_i} (m_i^a)^2 - \frac{1}{2} H_b \sum_i M_{0,i}^2 (m_i^b)^2$$

Carrying this change through the calculation leads to a change in the final set of equations where $H_{E_{ij}} \rightarrow H_{E_{ij}} M_{0,i} M_{0,j}$, $H_0 \rightarrow H_0 M_{0,i}$, $H_{a_i} \rightarrow M_{0,i}^2 H_{a_i}$ and $H_b \rightarrow M_{0,i}^2 H_b$ (the updated complete calculation is shown in SI section 4 and 5). For the minimal doping- and field-dependent model, we now use

$$M_{0,i} = 1 + \eta_M n_i$$

instead of a doping-dependent gyromagnetic ratio (eq. (5) of the main manuscript).

We have updated Fig. 3c of the main manuscript (Fig. R10) and the corresponding text passage accordingly:

"In contrast to conventional models, the macrospin length, interlayer exchange and intermediate axis anisotropy are taken to be explicitly layer-dependent. […] To determine compatible mechanisms, we analyze the dependence of the magnon modes on overall changes in M_0 (= $M_{0,t}$ = $M_{0,m}$ = $M_{0,b}$), H_a (= H_{at} = H_{am} = H_{ab}) and H_E(= H_{Etm} = H_{Emb}) in the numerical model within realistic ranges, see Fig. 3c-e. We observe that both f_{IP} and f_{OP} increase linearly with M_0 at similar rates (Fig. 3c)."

All fits in the updated manuscript now use this doping dependence.

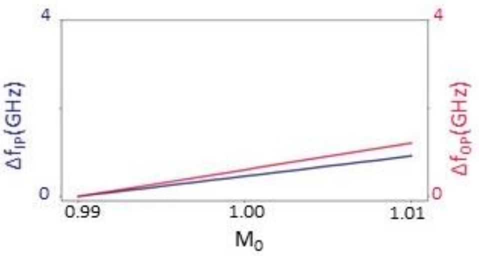

Fig. R10: Changes in the magnon frequencies as a function of macrospin length M_0.

Overall, I see bit of a gap between the phenomenological dependencies assumed to fit the experimental data and the physical expectations, suggesting further discussion on this issue.

We thank the reviewer once again for the thorough feedback. We hope that the measurement of additional samples, improved modeling and further discussion of remaining discrepancies between experimental and modeled behavior in the reviewed manuscript and SI can clear remaining doubts.

Reviewer #3 (Remarks to the Author):

Stetzuhn et al. demonstrated the gate-tunable magnons of CrSBr in a dual-gated configuration, and developed a model to explain the results, establishing the doping- and electric field-dependence of the exchange interaction, magnetic anisotropy and magnetic moment of individual layers. Overall, the topic is of interest in the field 2D magnetics, the results are solid and the explanations seems reasonable. My concerns focus on the novelty of both motivation and the proposed model: what are the differences between the previous and present work? My detailed comments are as following, and the editor makes the final decision.

We thank the reviewer for the constructive feedback. We have altered the text of the main manuscript to strengthen the arguments for novelty and the need for a layer-resolved model, as described in more detail in the following answer.

(1) The core of the manuscript is the tunability of magnon by electric gating. Given that the effect of gating on magnon energies and transport has been studied in other magnetic systems, the novelty of the present manuscript may not reach the standard of Nat. Commun. I suggest the authors describe the difference a little more in the Introduction part.

While the tunability of magnons has been experimentally studied in other two-dimensional systems such as Cr_3GeTe_2 [*Hendriks, F. et al., Nature Comms, 15:1, 1 (2024)*] and CrI_3 [*Zhang, X.-X. et al., Nature Materials, 19:8, 838 (2020)*], our manuscript focusses on a more detailed understanding of the tunability via a new layered-resolved macroscopin model. In particular:

- To the best of our knowledge, no previous study on magnons in van der Waals magnets has explicitly modeled their dependence on n and F. From our simple toy model, we can deduce which magnetic material parameters are most strongly influenced by electrostatic gating. In particular, we find a competing field-dependence of the interlayer exchange and anisotropy energies.
- The systematic layer-number dependence of magnon tunability in van der Waals magnets has not been studied before. The layer-number-dependent electrostatic tunability can only be explained in a layer-resolved model, since it results from increased screening in the center of the device. The model is applicable to a broad class of emerging van der Waals magnets beyond CrSBr.
- The separate tunability of the in- and out of phase magnon frequency of a 2D antiferromagnet by a perpendicular electric field has not been shown before.
- As a side note, we are also the first to show the electric field-dependent photoluminescence of CrSBr. We gain information on the energy-split excitons at 1.37eV which confirms their assignment as inner- and outer layer excitons, which has recently come under debate [*Shao, Y. et al., Nature Materials 24, 391 (2025)*].

We thank the reviewer for the suggestion and now emphasize these points more strongly in the introduction: "While the effect of gating on magnon energies and transport has been studied in a few selected 2D magnetic systems [33–36], the response of magnons in thin CrSBr to electrostatic gating remains unexplored. […] In this report, we address these questions by exploring the response of magnons in few-layer CrSBr devices to electro-static gating. We find an opposing trend of the in- and out-of-phase magnon mode to an electric field in a dual-gated trilayer device, which, to the best of our knowledge, has not been reported previously. The detailed understanding of such a response is especially important in the context of developing spintronic applications of nanostructured 2D

materials [39]. We therefore develop a minimal model connecting the layer-resolved charge carrier densities and electric fields to the magnetic properties of the sample. This allows us to explicitly correlate the measured magnon frequencies in CrSBr to its electrostatic properties using capacitive modeling and gate-dependent photoluminescence measurements."

(2) The range of the tunability of the magnon modes is only 10%. How does this value reflect the high tunability? What is the potential application of magnon in this narrow range? Besides, the in-plane frequency seems to be insensitive to the gating, so what is new here?

As shown below in Fig. R11(a), the in-phase frequence f_{IP} indeed exhibits a tunability of up to 2 GHz in the trilayer sample, a similar order of magnitude to that of the out of phase frequency f_{OP}. Interestingly, the two modes react differently to the application of a perpendicular electric field (Fig. R11(b, c)). To the best of our knowledge, this effect has not been seen previously, as other studies focused only on ferromagnetic samples with a single mode [*Hendriks, F. et al., Nature Comms, 15:1, 1 (2024)*] or doping effects in antiferromagnets [*Zhang, X.-X. et al., Nature Materials, 19:8, 838 (2020)*].

We emphasize that we see the aim of this work in the development of a new approach to understand gate- and field- induced magnon tunability via a layer-resolved macroscopin model, rather than establishing record-high magnon tunability. However, a tunability of 2.5 GHz is large enough to, e.g., use for the construction of magnonic logic gates as proposed in [*Rana, B. et al., Physical Review Applied, 9:1, 014033 (2018)*]. In a simple estimate, two magnons propagating in CrSBr strips of length *l* with different frequencies acquire a phase shift of $\Delta\phi = \frac{2\pi l \Delta f}{v_g}$. With $\Delta f = 2\ \mathrm{GHz}$ and a group velocity $v_g = 1\,\frac{\mathrm{km}}{\mathrm{s}}$ [*Bae, Y. J. et al., Nature 609:7926, 282 (2022)*] a phase shift of π (needed for destructive interference) can be reached within a gate length of half a micron.

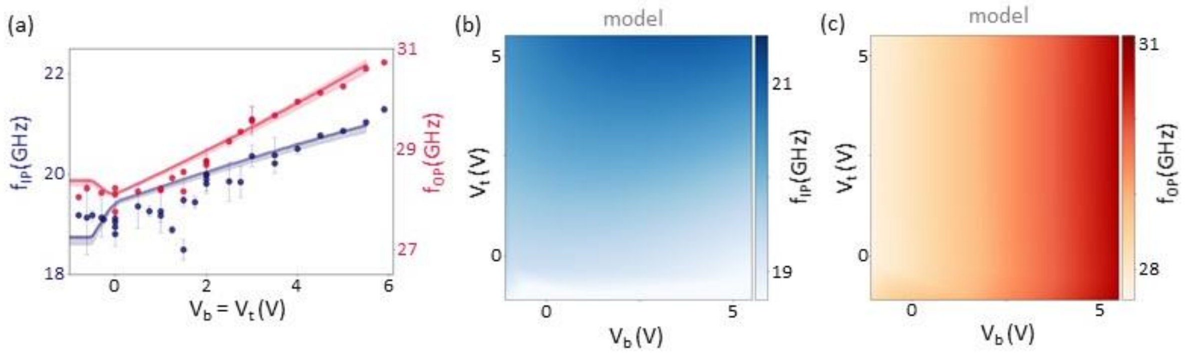

Fig. R11: Macrospin fitting of the magnon modes in the trilayer CrSBr device.

(3) The authors claim that they proposed a model to explain the data. I am wondering that the model is just a modified model or a new model proposed for the first time? Moreover, for the gate-tunable magnon in other magnetic materials, what kinds of models do they use? Are there any differences between the models in the present and previous work?

The model we are proposing – a layer-resolved macroscopin model – is a modification of the well-known Landau-Lifshitz (LL) model for magnons. In general, LL is the simplest model to treat magnetic excitations without considering damping. One key assumption in conventional LL is that every layer is the same, i.e. has the same exchange and anisotropy fields, with only the direction of H_E flipping in

every second layer for antiferromagnets. Also, an infinite thickness of the sample is implied by using periodic boundary conditions. While this model is well suited to describe many bulk materials, we believe that simple LL model breaks down in few-layered two-dimensional magnets:

- Surface effects gain importance when approaching the single layer limit – in a trilayer, two out of three layers are surface layers. This leads, for example, to substrate-dependent magnetic properties [*Zhang, L. et al., APL 116:4 (2020)*].
- In layered van der Waals semiconductors, electrostatic gating leads to different carrier densities across layers, as the inner layers are screened from the gates by the outermost ones. As carrier density and electric field influence the magnetic properties of the material, they can therefore also differ between layers. This can only be described in a layer-dependent model.

We therefore introduce a layer-dependent LL model, in which the magnetic properties can be different in each layer of the structure. While this makes an analytical solution impossible, we think that the model can reasonably reflect the layer-dependence of the magnon tunability we observe in experiments. As van der Waals magnets have garnered significant interest since their discovery, including within the field of spintronics, we believe that this type of model will be helpful for future studies on gate-tunable magnetic phenomena.

To the best of our knowledge, this is the first time such a layer-dependent model has been used to describe experimental data. A previous study on the gate tunability of the magnon mode in the 2D ferromagnet Cr_3GeTe_2 (CGT) [*Hendriks, F. et al., Nature Comms, 15:1, 1 (2024)*] used a bulk LL model including only one internal field (not differentiating, for example, between exchange and anisotropy effects). This model does not allow to draw any clear conclusion of the dependency of the internal field on *n* and *F*. A second study on the gate tunability of magnons in bilayer CrI_3 has not modeled the experimental data [*Zhang, X.-X. et al., Nature Materials, 19:8, 838 (2020)*].

(4) The authors chose the flakes with layer number of 3-8 layers. Why not choose bilayer flake? In my opinion, the doping of individual layer in bilayer CrSBr may be more suitable for the explanation the authors proposed.

We agree that the effect of electrostatic gating should be largest for a bilayer sample. We were initially dissuaded from measuring bilayers due to the strong drop in intensity of the time-resolved reflectivity signal reported in bilayer samples [*Bae, Y. J. et al., Nature 609:7926, 282 (2022)*].

Nevertheless, motivated by the reviewer's comment, we fabricated and measured two additional bilayer samples (one single- and one dual-gated). Consistent with the previous observations, we indeed observe a significant decrease in tr-reflectivity signal strength on a terraced sample. The signal intensity decreases by an order of magnitude when going from 8 to 2 layers (see Fig. R12(a) for an optical image of the sample and Fig. R12(b) for the corresponding signal amplitude). Nevertheless, we find sufficient signal strength on this bilayer sample to test its gating response.

First, however, we compare the extracted magnon frequencies on the different thickness steps of this sample (Fig. R12c). We find almost no thickness dependence of the lower f_{IP} mode, while the higher f_{OP} mode decreases by several GHz going to the bilayer region. The layer-dependent modes calculated from the macrospin model predict this behavior.

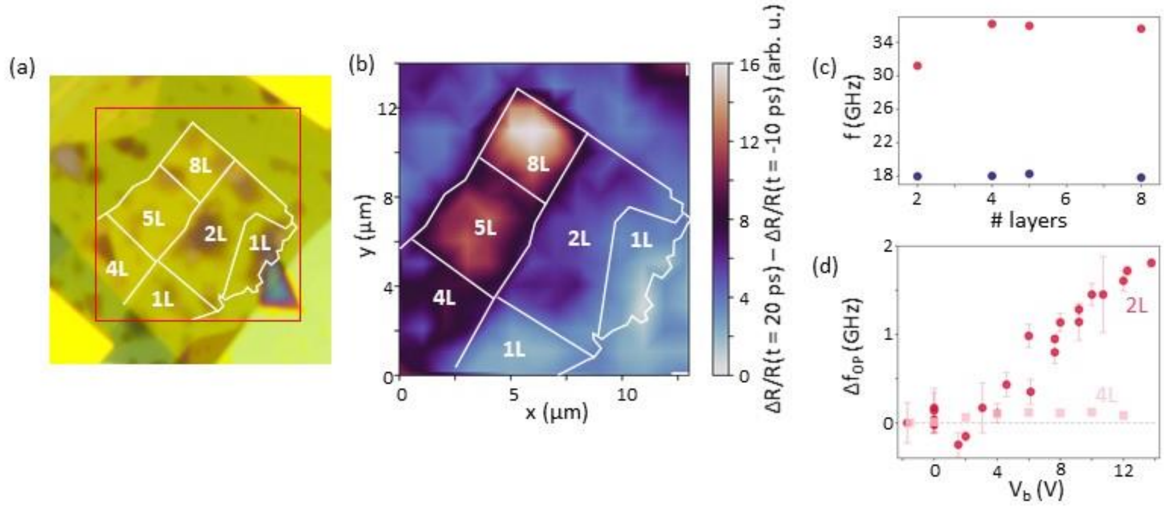

Fig. R12: Spatially resolved tr-reflectivity signal strength and magnon frequencies on a CrSBr sample containing 1-8 layer regions.

Next, we measure the bottom-gate dependence on the bilayer region of this single-gated sample and compare it to the tunability on the thicker parts. We find almost 2 GHz of tunability for f_{OP} on the bilayer (red data points in Fig. R12(d)), compared to < 0.3 GHz on the 4-layer region (pink data points in Fig. R12(d)).

The second new sample for this review is a double-gated bilayer sample. On this sample, the signal amplitudes were very weak, leading to a higher error and lower density of gate-dependent data points. The results of the doping-dependent measurements on the two bilayer samples are shown below (single-gated sample in Fig. R13(a) and dual-gated sample in Fig. R13(b)).

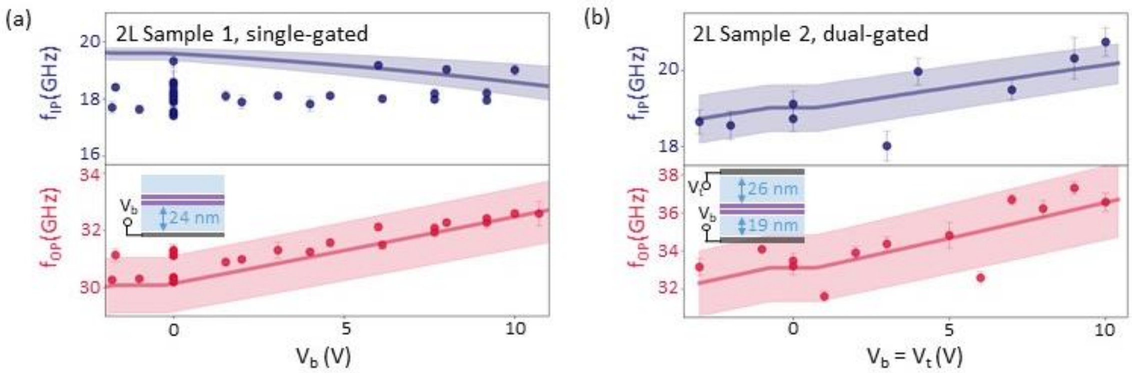

Fig. R13: Magnon tunability on a (a) single- and (b) dual-gated bilayer CrSBr sample.

We find that the maximum frequency shift does not surpass that of the trilayer significantly for either sample in the chosen range of gate voltages (limited by dielectric breakthrough of the hBN), which is in agreement with the modelled frequencies (solid lines with shaded error region in Fig. R13). We emphasize here that the model applies doping- and field-dependencies obtained for the trilayer CrSBr and fits the bilayer very well – further confirming its predictive power. For the dual-gated sample, we find a maximum shift of $\Delta f_{IP} \sim 2$ GHz and $\Delta f_{OP} \sim 4$ GHz. Due to the superior data quality on the trilayer sample, and the negligible increase in tunability for the bilayer, we keep the trilayer sample data as the central Fig. 1 of the main manuscript. We added the bilayer data of the single-gated bilayer sample to Fig. 4 of the main manuscript and the remaining new data to the SI section 8. Table 1 of the main manuscript now also includes the fit results of the bilayer samples.

(5) For the fitting, the authors used the calculated value of dielectric constant of CrSBr, and the authors used the fitting results to demonstrate the accuracy of their models. Given that this value is strongly dependent on the layer number, and the calculated value may be inconsistent with the experimental value, I suggest the authors give an experimental value of dielectric constant, and its layer-dependence for fitting. This is more reasonable, I think.

To the best of our knowledge, there has been no experimental report of the DC dielectric constant of CrSBr along the c-axis. Since CrSBr is a semiconducting material, measuring the dielectric constant – especially in the limit of a few layers and zero frequency – is challenging. We believe that the value we calculated by DFT is reliable; it is also comparable to values used in other studies for hBN-encapsulated bilayer CrSBr, such as ε = 4.5 [*Semina, M. A. et al., PRB, 111:20, 205301 (2025)*]. We also note that small changes in the dielectric constant do not significantly alter the resulting charge carrier densities, so that any error due to an inaccuracy in ε should result in fitting values within the error margin given in Table 1 of the main manuscript.

(7) The experimental results in Figures 1d, e are quite different from the simulated counterparts in Figures 1f, g. I am confused that why the authors claim that the experimental data are in good agreement with the theoretical values?

In the review process, we identified possible improvements to the fitting and received a valuable comment from reviewer 2 in point #6. As a result, to improve the fits of the macrospin model to the trilayer data, we:

- Identified and corrected an erroneous capacitance in the capacitive model leading to more accurate electron density *n* and perpendicular electric field F_z
- Included the anisotropy field H_b in the fitting parameters (instead of fixing it in the previous model iteration)
- Changed the dependency in Eq. (5) from a doping-dependent gyromagnetic ratio to a doping-dependent macrospin length: $M_{0,i} = 1 + \eta_M n_i$. This is discussed in more detail in the answer to the last question by reviewer 2.

The resulting changed plots are shown in Fig. R14, where (a) shows the doping dependence of f_{IP} and f_{OP} (points: experimental data, solid lines: macrospin model fits). We also show the altered fits for the complete gate-dependence of (b) f_{IP} and (c) f_{OP} (Fig. R14). The corresponding plots in Fig. 1 of the main manuscript were updated accordingly.

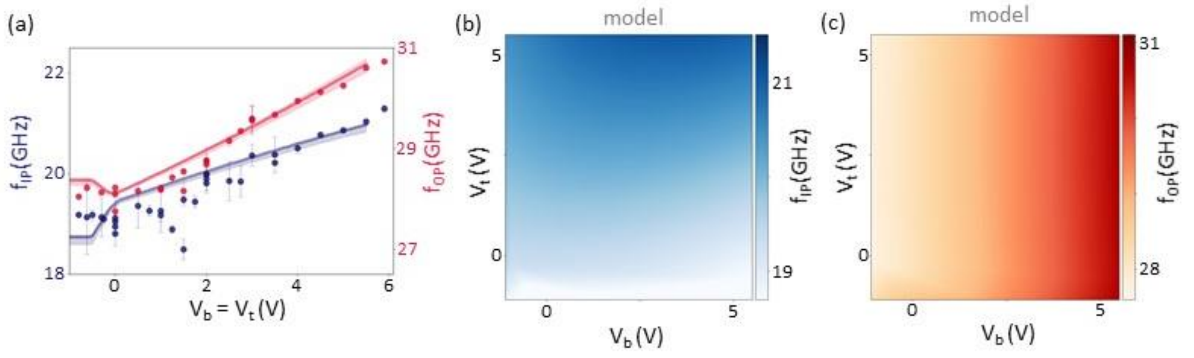

Fig. R14: Macrospin fitting of the magnon modes in the trilayer CrSBr device.

The overall agreement between data and fits has improved, in particular for f_{OP}. We note that there are still some discrepancies left in certain gate voltage regimes such as the delayed onset of magnon frequency shift with doping in Fig. R1 (a). This could indeed indicate that there is an additional threshold or non-linearity at low carrier densities and fields, which we do not take into account in the fit functions in Eq. (3)-(5). We discuss possible effects of doping and electric field on the magnetic properties of CrSBr in SI section 8:

"(1) By increasing the electron doping n, the magnitude of the macrospin can increase due to an increase in carriers with magnetic moment. The Coulomb repulsion parameter U can also increase with n, possibly influencing the exchange constants according to the Kugel-Khomskii model. (2) When doping with electrons, e_g *levels will be populated in addition to* t_{2g} *ones. This can change the orbitals involved in the hopping between sites, again affecting the (anti-)ferromagnetic exchange coupling parameters. Additionally, the different orbital shapes can influence the anisotropy of the material. (3) A perpendicular electric field between layers can shift the energy positons of bands associated with different orbitals in neighboring layers with respect to each other, again influencing the (anti)ferromagnetic exchange coupling parameter. (4) Gates can influence orbital filling especially in the outer layers due to the resulting electric field, as electrons shift further/closer to the gate, shuffling them into* $d_{x^2-y^2}$ *or* d_{z^2} *orbitals, respectively. This is commonly called voltage-induced magnetic anisotropy."*

Additionally, we discuss different fitting approaches in SI section 9 to support the chosen dependencies.

(8) In Figure 2a, there is an intensity drop when Vb = Vt = ~ -0.6 V. How to explain it? Besides, given that the authors estimated the carrier density of top layer, middle layer and bottom layer in Figure 2d, the value of individual layer is accurate or not? If the layer number is larger than 3, is it reasonable to estimate the carrier density or magnetic moment of individual layer for further explanation or fitting?

The emission around 1.374 eV in Fig. 2a is the photoluminescence of charge neutral excitons in CrSBr [*Tabataba-Vakili, F. et al., Nature Comms 15:1, 4735 (2024)*]. We assign the intensity drop around $V_b = V_t = -0.8$ V in Fig. 2a to the increased formation of charged excitons (trions) consisting of two electrons and one hole due to the surplus of electrons at higher gate voltages. Trions have an emission at lower energies around 1.35 eV. The intensity drop at 1.374 eV is simultaneous with an intensity increase at 1.35 eV, consistent with the interpretation of trion formation. This behavior is typical of gated two-dimensional materials and has been reported in TMDs as well as CrSBr [*Tabataba-Vakili, F. et al., Nature Comms 15:1, 4735 (2024)*]. One can hypothesize that the sudden drop in intensity results from the flat conduction band in CrSBr, leading to a van't Hovt singularity in the density of states [*Wu, F. et al., Advanced Materials, 34:16, 2109759 (2022)*], and therefore a strong increase in the number of electrons as soon as the band is reached.

The fact that the emission energy of excitons and trions in the outer and middle layers of the trilayer are slightly different allows us to draw conclusions on the doping level in the different layers of the device. We use this to estimate the free parameters in the capacitive modeling of the carrier densities in each layer, leading to possible inaccuracies in doping onsets. In this model adapted from [*Pisoni, R. et al., PRL 123:117702 (2019), Kovalchuk, S. et al., Nature Comms 16:9893 (2025)*], we explicitly calculate the electron densities in each individual layer, which in that sense are accurate within the boundaries given by underlying assumptions – in particular the built-in electric field, intrinsic doping level or density of states. We do the same for samples of other thicknesses and

extract the doping levels of each individual layer. The capacitive modeling is described in detail in SI section 4 for all samples.

(9) There are some typos, especially in References part. The authors should carefully check them.

We thank the reviewer for pointing out typos and have carefully checked and corrected for mistakes in the article and references.

Reviewer #4 (Remarks to the Author):

The manuscript by Nele Stetzuhn et al. reports electrically tunable magnons in the dual-gated 2D antiferromagnet CrSBr, with frequencies reaching up to 2 GHz. By employing a time-resolved pump–probe technique, the authors systematically characterize magnon frequencies under varying doping levels and electric-field orientations. The observed frequency shifts with carrier density are attributed to a layer-dependent gyromagnetic ratio, interlayer exchange interactions, and magnetic anisotropy. Magnons in CrSBr are of considerable current interest in the field of antiferromagnetic materials. Nevertheless, several issues should be addressed before the manuscript can be considered for publication in Nature Communications.

We thank the reviewer for the encouraging words and valuable feedback in the response.

1) The authors report two oscillation frequencies of the magnon at 19 and 28 GHz, corresponding to the in-phase and out-of-phase zero-momentum magnon modes. However, according to Fig. 3 of Ref. [Phys. Rev. B 109, 104401], the optical (Op) and acoustic (Ac) interlayer AFM magnon modes appear at 24.6 and 33.4 GHz, respectively. Could the authors clarify the origin of this discrepancy in magnon frequencies?

The frequency of the magnon modes generally is influenced by external factors, such as the applied magnetic field or temperature (and, accordingly, pump laser fluence), as well as the internal magnetic properties of the material, i.e. the exchange and anisotropy parameters. The latter can vary across measurements done by different groups, for example due to different growth methods of the CrSBr crystals as well as strain or residues introduced by exfoliation and transfer. The reported frequencies in similar conditions to our measurements (H_0 = 0.1 T, T = 10 K) vary across papers: ∼24 and 34 GHz [*Bae, Y. J. et al., Nature 609:7926, 282 (2022)*], ∼21 and 32 GHz [*Sun, Y. et al., Nature Physics 2024, 1-7 (2024)*], ∼20 and 29 GHz [*Diederich, G. M. et al., Nature Nano 18:1, 1 (2022)*]. Across experiments we have conducted on different devices from the same mother crystal, we have observed a range of frequencies for f_{IP} = 17.5 - 22 GHz and f_{OP} = 28 - 33.5 GHz.

We also note that other studies have focused on bulk CrSBr, while we performed our measurements on few-layer devices. We found a thickness-dependent change in the magnon frequencies on a newly measured sample (optical image and signal intensity map in Fig. R15(a,b) below). Going from 8 to 2 layers, the higher frequency f_{OP} decreases by several GHz, while the lower frequency f_{IP} is almost unaffected (Fig. R15(c)). Interestingly, this is also captured in our macrospin model, where f_{OP} drops significantly when going below 4 layers while f_{IP} remains relatively unchanged (Fig. R15(d)). We show this data in SI section 10 of the updated manuscript.

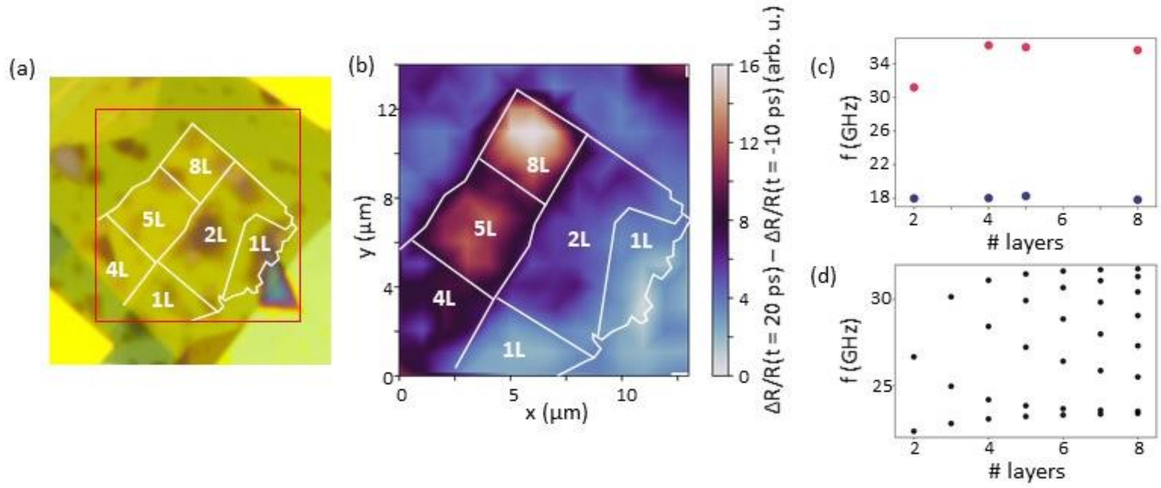

Fig. R15: Spatially resolved tr-reflectivity signal strength and magnon frequencies on a CrSBr sample containing 1-8 layer regions. Experimental layer-number-dependent frequencies in (c) and model predicitons in (d).

Additionally, the cited reference employs MOKE to determine the magnon modes, whereas the present work uses a time-resolved pump–probe technique. What are the differences between these two approaches in determining the magnon modes?

It is possible to observe the magnon oscillations using time-resolved polar MOKE, as shown in [*Bae, Y. J. et al., PRB, 109:10, 104401 (2024)*]. The polar MOKE geometry is sensitive only to changes in the out-of-plane magnetization, i.e. only the component of the magnon oscillations along the z-direction. This signal can become rather weak, making tr-reflectivity the perfect alternative due to the magnon-exciton coupling in CrSBr. This coupling leads to the modulation of the time-resolved reflectivity signal by magnons with a very good signal-to-noise ratio, as previously reported [*Dirnberger, F. et al., Nature 620:7974, 533 (2023)*, *Sun, Y. et al., Nature Physics 2024, 1-7 (2024)*, *Bae, Y. J. et al., Nature 609:7926, 282 (2022)*]. We have added the following explanation to the manuscript: "The excitons in CrSBr around 1.37 eV exhibit a strong coupling to the magnetic order of the sample: Depending on the orientation of the macrospins in neighboring layers, the interlayer character and degree of localization of the excitons differs [40–42]. This results in a change of their binding energy, and accordingly their energetic resonance position in photoluminescence and reflectivity spectra [22, 23, 25]. Oscillations in the magnetic order can therefore be seen as oscillations in the tr-reflectivity signal of the sample [21, 26, 28]."

2) In CrSBr, the reported frequency shift under dual-gate tuning is around 2 GHz. By comparison, in CrI_3 the magnon frequency can be tuned from 80 to 55 GHz when the gate voltage is varied from –13 to 13 V [Nature Materials volume 19, pages838–842 (2020)]. What, then, is the specific advantage of CrSBr under gate tuning? I suggest that the authors clarify the significance of this result and emphasize it in the Introduction."

There are several material-specific advantages of CrSBr:

- CrSBr has proven to be more air-stable [*Ziebel, M. E. et al., Nano Letters 24:15, 4319 (2024)*] than materials of the chromium halide family such as CrI_3 - which dissolves in air within seconds without proper encapsulation [*Shcherbakov, D. et al., Nano Letters, 18:7, 4214 (2018)*] - making it a stronger candidate for future spintronic devices.

- The layer dependent excitons in CrSBr [*Tabataba-Vakili, F. et al., Nature Comms 15:1, 4735 (2024)]* allow a layer-resolved estimation of the carrier density – enabling us to model the field- and doping-related response in this work.
- The magnons in CrSBr have a long coherence time exceeding 1 ns, allowing for long-range magnon transport [*Bae, Y. J. et al., Nature 609:7926, 282 (2022)*].
- The magnon-exciton coupling in CrSBr allows easy accessibility of the magnons through tr-reflectivity [*Dirnberger, F. et al., Nature 620:7974, 533 (2023)*, *Sun, Y. et al., Nature Physics 2024, 1-7 (2024)*, *Bae, Y. J. et al., Nature 609:7926, 282 (2022)*].
- CrSBr is a quasi 1D material [*Wu, F. et al., Advanced Materials, 34:16, 2109759 (2022), Klein, J. et al., ACS Nano, 17:6, 5316 (2023), Smolenski, S. et al., Nature Comms 16, 1134 (2025)*] and hosts magnon-coupled polaritons in the bulk [*Dirnberger, F. et al., Nature 620:7974, 533 (2023)*]. The interplay of several tunable phenomena makes CrSBr a rich playground for new physics.

We emphasize that previous studies of gate-tunable magnons have not offered a model directly connecting charge carrier densities and electric fields to magnetic material properties [*Hendriks, F. et al., Nature Comms, 15:1, 1 (2024), Zhang, X.-X. et al., Nature Materials, 19:8, 838 (2020)*]. The layer-resolved LL model allows us to extract which parameters governing magnon dynamics are most strongly affected by charge carrier density *n* and perpendicular electric field F_z, namely the macrospin magnitude and the anisotropy/exchange fields. We believe that our model can be extended to other van der Waals magnets in the future.
Finally, we note that we see the aim of this work in the development of a new approach to understand gate- and field- induced magnon tunability via a layer-resolved macroscopin model, rather than establishing record-high magnon tunabilty. We have therefore rephrased parts of the introduction to add more emphasis on this aspect of our work: "While the effect of gating on magnon energies and transport has been studied in a few selected 2D magnetic systems [33–36], the response of magnons in thin CrSBr to electrostatic gating remains unexplored. [...] In this report, we address these questions by exploring the response of magnons in few-layer CrSBr devices to electro-static gating. We find an opposing trend of the in- and out-of-phase magnon mode to an electric field in a dual-gated trilayer device, which, to the best of our knowledge, has not been reported previously. The detailed understanding of such a response is especially important in the context of developing spintronic applications of nanostructured 2D materials [39]. We therefore develop a minimal model connecting the layer-resolved charge carrier densities and electric fields to the magnetic properties of the sample. This allows us to explicitly correlate the measured magnon frequencies in CrSBr to its electrostatic properties using capacitive modeling and gate-dependent photoluminescence measurements."

3) As variations in carrier concentration are generally expected to influence the spin–exchange interaction and thereby affect the magnon frequency, could the authors clarify how this aspect has been considered in their analysis?

We agree. Many field- and carrier-density-dependent processes, including changes in the Coulomb repulsion or hopping parameters influencing the exchange interaction, can affect the magnons (see SI section 8 for detailed description).

This complexity makes it difficult to include all possible effects of charge carrier density *n* and electric field F_z. Instead, we aim find a minimal model that captures the dominating processes by comparing different fitting approaches in our macrospin model. The logic of our argument (now added to the SI section 9) is below.

"The most intuitive consequences of an increased electron density in the sample are an increase of the layer magnetization as well as an increased interlayer exchange interaction. We therefore start by fitting the trilayer data only considering doping-dependent linear effects, explicitly

- $M_{0,i} \propto n_i$,
- $H_{A,i} \propto n_i$ *and*
- $H_{E,ij} \propto n_i + n_j$.

We show the difference between experimental (f_{ex}) and fitted (f_{mod}) magnon frequencies in Fig. R2 (a) (top: $f_{IP,ex} - f_{IP,mod}$, bottom: $f_{OP,ex} - f_{OP,mod}$). In the resulting Voronoi plots, white/faint colors correspond to small differences between the fit and the experimental plot, while bright red/blue signify larger deviations. Unsurprisingly, the resulting fits fail to capture any field-dependent changes of the magnon frequencies, seen by large deviations in the residual frequencies for $V_b \neq V_t$. From this, we conclude that at least one of the magnetic properties should be dependent on the perpendicular electric field F_z.

Therefore, we choose an electric field dependence of the interlayer exchange interaction in the fits, i.e.

- $M_{0,i} \propto n_i$,
- $H_{A,i} \propto n_i$ *and*
- $H_{E,ij} \propto F_{z,ij}$

In Fig. R2 (b). To avoid overfitting, we neglect the doping dependence of H_E. This model indeed fits the gate dependence of the f_{OP} mode better, visible in the more homogeneous and smaller deviations in the bottom plot of Fig. R2 (b). However, it still does not capture the field dependence of f_{IP}. This leads us to believe that in order to fit the field-dependence of both modes, we need to include two competing parameters $\propto F_{z,ij}$.

We therefore include a second field-dependence, choosing

- $M_{0,i} \propto n_i$,
- $H_{E,ij} \propto F_{z,ij}$ *and*
- $H_{A,i} \propto F_{z,i,i+1} + F_{z,i-1,i}$.

This captures the gate-dependencies of both f_{IP} and f_{OP} reasonably well (Fig. R2 (c)). The average difference between the modeled and experimental f_{OP} is < 0.2 GHz. The largest deviations in the residual frequency can be seen in the low carrier density regime around V_b = V_t = 0–2 V. This is due to the delayed onset of the frequency shifts also visible in Fig. 1c of the main manuscript. For f_{IP}, we can see that at high absolute field values F_z the fits deviate the most from the experimental behavior (up to 0.7 GHz), possibly indicating an additional phenomenon taking effect at those gate voltages. However, we also note that in the measurements on the trilayer device, the lower frequency mode had a weaker signature in the FFT, leading to a larger error when extracting its position. Overall, the average difference between the experimental and modeled f_{IP} is < 0.3 GHz.

Lastly, we examine whether including an additional doping dependence of the exchange interaction improves the fits further using

- $M_{0,i} \propto n_i$,
- $H_{E,ij} \propto n_i + n_j$,
- $H_{E,ij} \propto F_{z,ij}$ *and*

- $H_{A,i} \propto F_{z,i,i+1} + F_{z,i-1,i}$

In Fig. R2 (d). We see no significant improvements in the fits compared to those in Fig. R2 (c). We therefore neglect any dependencies beyond those shown in Fig. R2 (c) to avoid high errors of the fit parameters resulting from overfitting."

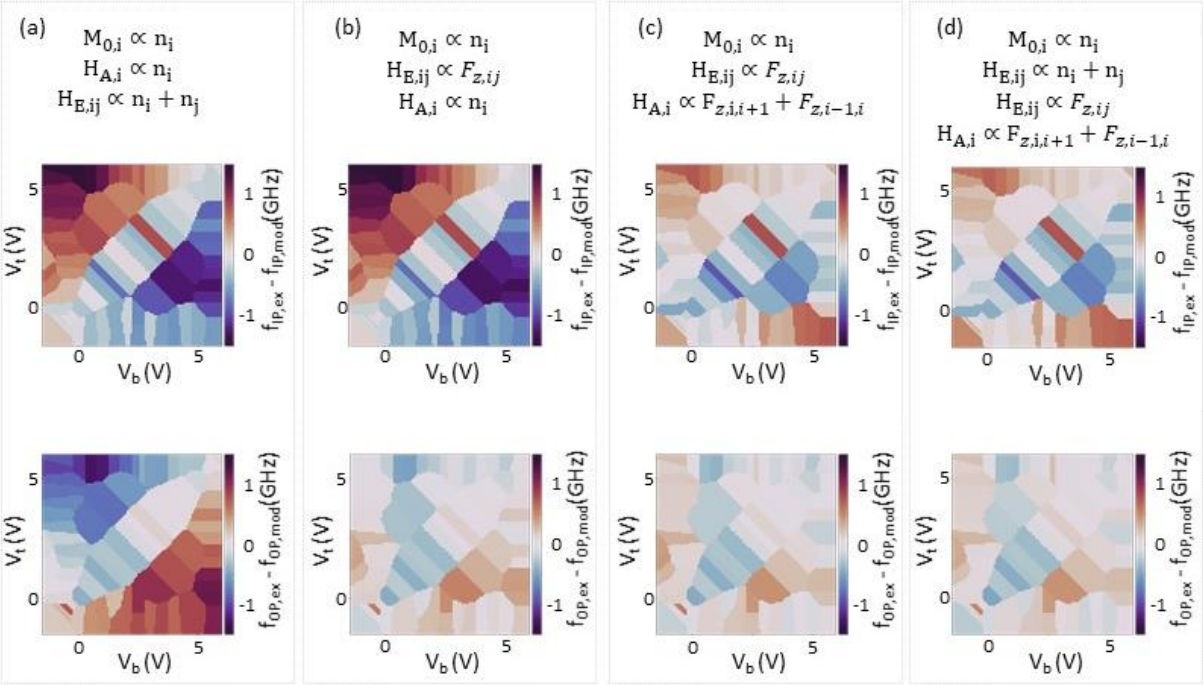

Fig. R16: Difference between fitted (f_{mod}) and experimental (f_{ex}) gate-dependent magnon frequencies in the trilayer CrSBr sample for different model dependencies on n and F. (a) to (d) refer to different fitting models described in the text (fit dependencies at the top); the color scale is the deviation between the fitting model and the data. The top row shows f_{IP}, the bottom row f_{OP}.

4) In Fig. 2a, the PL emission appears to suddenly drop around –1 eV. As reported in Ref. (Nat. Commun. 15, 4735 (2024)), the XB and XB⁻ emissions gradually disappear with increasing reverse voltage. Could the authors clarify what happens at this point?"

With an increasing gate voltage, the Fermi level in the sample is shifted upwards. The resulting surplus of electrons when entering the conduction band of CrSBr leads to the formations of charged excitons, i.e. negative trions. We attribute the intensity drop of the neutral exciton emission around $V_b = V_t = -0.8$ V to the onset of said formation. The trion emission is visible at lower energies around 1.35 eV, coming up simultaneously to the intensity decrease around 1.374 eV.

The difference in the behavior of the X_B and X_B' excitons between [*Tabataba-Vakili, F. et al., Nature Comms 15:1, 4735 (2024)*] and our manuscript stems from the response of the excitons to single- vs. double-sided gating. When gating from one side only, electrons mainly accumulate in the layer closest to the gate, screening the ones further away from it. Due to this, in Fig. 1i of [*Tabataba-Vakili, F. et al., Nature Comms 15:1, 4735 (2024)*], the authors see emission from the exciton X_B' (residing in the middle layer) for all gate voltages with only a small decrease in brightness with positive gate voltages. This leads us to conclude that the middle layer was not strongly doped in this experiment, supported by the fact that only one trion species (X_B^-) was observed.

In our experiments we see a similar behavior if we vary only the bottom gate voltage, fixing V_t = -0.65 V (right side of Fig. R17). Across the whole range, we see emission from both excitons. There is a sharp drop in intensity of X_B (blue) below V_b = 0 V which we assign to the bottom layer becoming doped, but the intensity of X_B' (green) remains unchanged due to the low amount of charge carriers in the middle layer. Using both gates, however, the amount of injected electrons is overall higher and happens from both the top and the bottom side of the device. Due to this increased gating efficiency, we see a drop in intensity of the PL of both excitons when changing the gates simultaneously (left side of Fig. R17). We interpret that in our trilayer device, the middle layer can only be strongly doped in a dual-gated sample architecture.

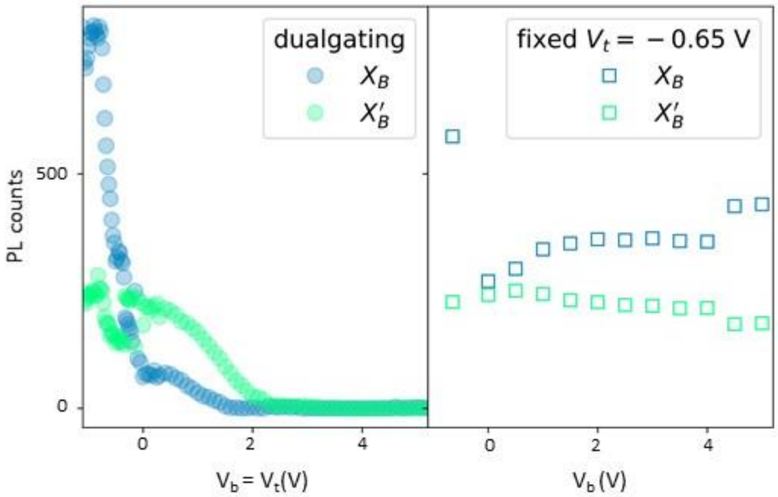

Fig. R17: Gate-dependent PL intensity of the X_B and $X_{B'}$ exciton in the trilayer when using both gates (left) or only the bottom gate (right).

5) Minor typos need revision; for instance, 'their' appears twice on line 29, page 1.

We thank the reviewer for pointing out typos and have carefully checked and corrected for mistakes in the article.

Reviewer #1 (Remarks to the Author):

The revised manuscript includes new data, a modified macrospin model, and additional discussions in response to the reviewers' comments, most concerns appear to have been addressed satisfactorily.

We thank the reviewer for the positive feedback on the revised manuscript.

However, the lower panel of Fig. 2(d) in the revised version differs noticeably from that in the original submission. The authors should clearly explain the modifications and justify this change before a definitive recommendation can be made.

The modifications in the lower panel of Fig. 2(d) are the result of the following changes to the capacitive model:

- We have corrected an error in the original simulation script that caused incorrect capacitance values for the hBN dielectric. We thank the reviewers again for helping us find this issue.
- The capacitance correction entailed changes to the model's offset gate voltages $V_{b,0}$ and $V_{t,0}$, which determine the built-in electric field F_0. $V_{b,0}$ remained unchanged at 1.7 V, while $V_{t,0}$ changed from 1 V to 0.6 V. With the corrected capacitance of the top and bottom hBN, we have found that the layer-resolved charge carrier densities n_i follow the exciton-to-trion ratios in the gate-dependent photoluminescence measurements more closely with this built-in field (SI section 4).
- The modified capacitance and built-in field also cause a correction to the charge-carrier densities as a function of $V_b = V_t$, as shown in Fig. 2(d) in the manuscript. The changes are especially prominent in the top layer, n_t, due to the change in $V_{t,0}$.

The changed charge carrier densities in Fig. 2(d) have been included in the subsequent macrospin modelling in the revised version. We note that these alterations did not affect the overall message of our work.

Reviewer #2 (Remarks to the Author):

The authors have adequately addressed my comments through additional measurements, theoretical analysis and clarifications. I now find the manuscript to be essentially ready for publication in Nature Communications.

We thank the reviewer for the positive assessment of the revised manuscript.

I have one remaining question, which did not become fully clear from the authors' response and that the authors could consider clarifying:

Figure 1b describes how the transient reflectivity data is converted to magnon frequencies, by attributing the oscillations to magnetization dynamics. This has been done using the dependence of the exciton energy on the magnetic canting, as the authors have further clarified in their previous response. However, in Fig. 2, the authors show closely spaced exciton and trion peaks in the PL spectrum. Now, do the trions play any role in the data of Fig. 1b? Or can one completely disregard the trions there? If the trions cannot be disregarded, how does one expect the dependence of the trion energy on the magnetic configuration to be? In the authors' response to my related question in the last round, they only discussed excitons and exciton-magnon coupling, as far as I can tell.

We find that coherent magnons affect the time-resolved reflectivity at both the trion and neutral exciton resonances. At an intermediate gate voltage, i.e. $V_b = V_t = 3$ V, we can detect magnon oscillations when probing at either energy (FFT of the time traces in Fig. R1(a)). Critically, we find the same frequencies, corresponding to f_{IP} and f_{OP}. We attribute the sensitivity of the trions to magnons to their coupling to the magnetic order in CrSBr, in a similar fashion to magnon-exciton coupling. The supressed interlayer electron tunnelling for anti-aligned macrospins in neighbouring layers influences the binding energy of the (mostly) intralayer trions, shifting their resonance energy. The energetic shift of the trions as a result of spin canting in an external magnetic field has been shown in magneto-photoluminescence measurements by [*Tabataba-Vakili et al., Nature Communications 15, 4735 (2024)*].

This trion-magnon coupling indeed plays an important role in the data of Fig. 1(b-e), as the strength of the time-resolved reflectivity signal – and the amplitude of the magnon oscillations – depends on the doping level of the system, at both the exciton and trion resonance. In Fig. R1(b), we show gate-dependent photoluminescence as well as differential reflectivity (at pump-probe delay 5 ps) spectra for select gate voltages. For negative gate voltages ($V_b = V_t = -1$ V), there is no trion feature in the photoluminescence and the ΔR/R spectra. Hence, to detect magnons in this gate voltage regime, we set the probe energy to the exciton resonance, around 1.376 eV (marked as "probe energy exciton" in Fig. R1(b), bottom panel). We note that the spectral width of the probe pulse, ~ 0.01eV, is narrower than the exciton-trion separation.

As the gate voltage and carrier density increase, the trions become the dominant feature in both spectra. Simultaneously, the amplitude of the tr-reflectivity traces at the exciton resonance decreases, before mostly vanishing for gate voltages above $V_b = V_t = 3$ V (green curve in Fig. R2(b), bottom panel). To detect magnons with an optimal signal-to-noise ratio, we therefore set the probe energy to the trion resonance in this regime, around 1.35 eV (marked as "probe energy trion" in Fig. R1(b), bottom panel).

To improve the clarity on this point, we have modified the corresponding section in the manuscript:

“The magnons are excited with an ultrafast laser pulse tuned to the exciton or trion resonance at 1.376 eV or 1.350 eV, respectively. Subsequently, we probe the tr-reflectivity response at the same wavelength. We tune the pump- and probe energy to either resonance depending on the doping level of the system to optimize the signal-to-noise ratio in the time-resolved reflectivity measurements (SI section 3).”

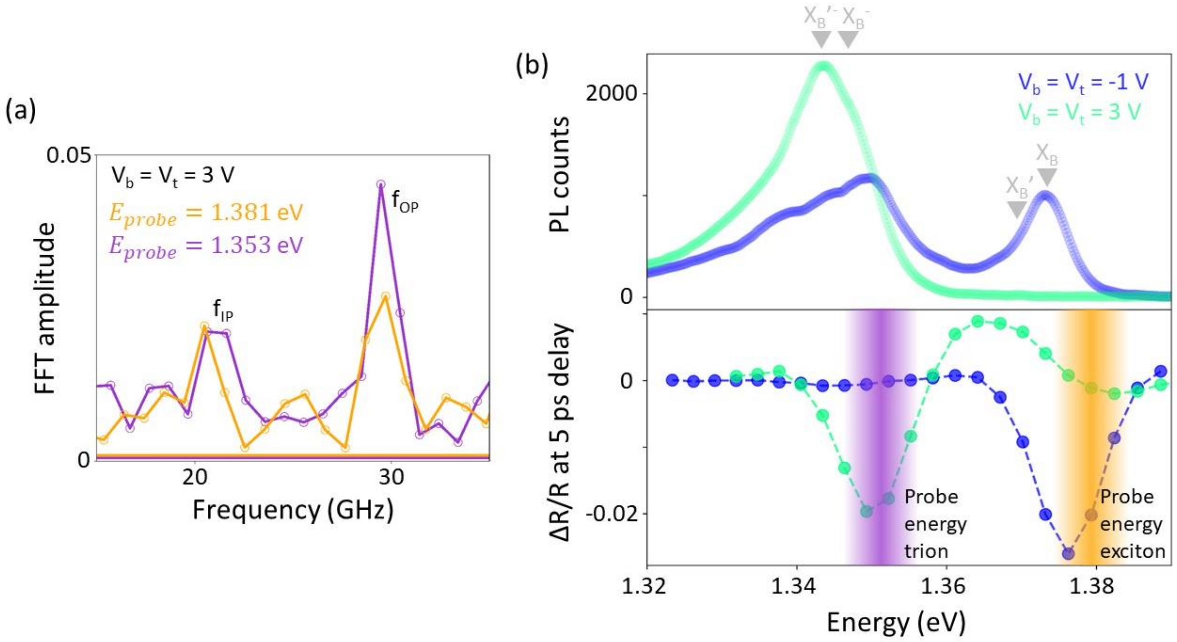

Fig. R1: (a) FFT of tr-reflectivity traces taken at an intermediate gate voltage (V_b = V_t = 3V) with the probe energy tuned to the exciton (orange) or trion (purple) resonance marked in (b). The frequencies f_{IP} and f_{OP} are the same at both probe energies. **(b)** Representative gate-dependent PL (top) and ΔR/R (bottom) spectra. Depending on doping, the pump/probe energy in the tr-reflectivity measurements is tuned to the neutral exciton (X_B and X_B', orange) or trion (X_B^- and $X_B'^-$, purple) resonances.

Reviewer #3 (Remarks to the Author):

I have carefully read the revised manuscript and the response, and greatly appreciate the efforts from the authors. My comments have been addressed, and I recommend it for publication as is.

We thank the reviewer for the kind words and the recommendation for publication of the revised manuscript.

Reviewer #4 (Remarks to the Author):

After reviewing the revised manuscript, I am generally satisfied with the authors' responses.

We thank the reviewer for the positive response to the revised manuscript.

However, there are still several issues that remain unclear or difficult to follow:
1. In Figure 2b, the gray curve is labeled as n = 0. I do not think this is an appropriate description. Although the Fermi level lies within the bandgap in this case, the carrier density n is not strictly zero. The authors are encouraged to adopt a more accurate expression.

We agree that the charge carrier density in the sample is never strictly zero due to, e.g., spatially varying charge puddles or defects [*Shin et al., Advanced Materials 28 (2016), Ersfeld et al., Nano Letters 20, 5 (2020)*]. We have therefore changed the label and the mentions in the text to $E_{\mathrm{V}} < E_{\mathrm{F}} < E_{\mathrm{C}}$ to more clearly indicate that we are treating the case where the Fermi level lies in the band gap.

2. On page 2, the authors state:
"Around 1.34 eV, we find an amalgamation of several weaker peaks even in the absence of trions (as n = 0), which can be assigned to the XA exciton (transition between the top valence band and the bottom conduction band) and phonon replicas [15,22,47,48]."
This suggests that the peaks around 1.34 eV originate from XA exciton and phonon replicas without doping. However, in Figure 2, the relative intensities of the gray and purple (yellow) curves appear to be of the same order of magnitude. Therefore, it is unclear whether the contribution from XA has been excluded when evaluating the intensities of XB⁻ and XB′⁻. The authors should clarify this point.

In the doping-dependent photoluminescence (PL) map shown in Fig. R2(a), we see a rapid increase in the emission intensity around 1.34-1.35 eV above V_b = V_t = -0.8 V (marked by the red box in Fig. R2(a)), which we attribute to trions due to their strong gate-voltage dependence. However, as pointed out by the reviewer, we detect emission in the same energy region before this sharp increase in counts. This has been attributed to the X_A exciton and phonon replicas of X_B [*Klein et al., ACS Nano, 17 (2023), Wilson et al., Nature Materials, 20 (2021), Tabataba-Vakili et al., Nature Communications 15, 4735 (2024), Lin et al., Journal of Physical Chemistry Letters, 15 (2024)*]. We assume that these additional peaks persist above V_b = V_t = -0.8 V, and therefore include them in the fits of the PL spectra as a (weakly gate-dependent) background, therefore excluding their influence on the trion intensities.

We show the doping-dependent PL spectra between V_b = V_t = -0.8 V and V_b = V_t = -0.5 V with the respective fitted excitonic peaks X_B and X_B' and trions X_B^- and $X_B'^-$ in Fig. R2(b). We indicate the fitted background (consisting of multiple gaussians) with dashed lines. The intensity of the X_B^- trion increases by a factor of ~5 going from -0.8 V (yellow in Fig. R2(b)) to -0.7 V (orange in Fig. R2(b)), while the background remains comparable. It intensifies by another factor of ~2 when rising the gate voltages by a further 0.2 V. Even though the background (stemming partially from X_A) is comparable or larger than the trion intensities, we can reliably fit their intensity.

To summarize, for the fits of the gate-dependent PL spectra in the manuscript we use

- two Gaussian peaks to describe the excitons X_B and X_B' around 1.37-1.375 eV
- two Gaussian peaks to describe the trions X_B^- and $X_B'^-$ around 1.34-1.35 eV

- a weakly gate-dependent background of four Gaussians between 1.32-1.36 eV to describe the background visible in this energy range. This is the minimum amount of peaks necessary to adequately fit the spectra without significant trion contributions ($V_b = V_t < -0.8$ V). We keep the number of background peaks constant for all spectra.

Strictly fixing the background fit parameters to the same values fitted for $V_b = V_t < -0.8$ V leads to slight deviations of the fits from experimental data at higher gate voltages, likely because peaks such as the X_A exciton also depend on V_b and V_t. However, we found that small gate-dependent changes of the fitting parameters of the background do not significantly change the overall gate-dependence of the trion intensities – particularly the trion onset voltage that we use to infer the doping level of the sample in the manuscript, which is visible by eye in Fig. R2(a).

We have added a short description of the fitting procedure of the gate-dependent PL spectra in SI section 4 to improve clarity on this point, and have removed the description of the background peaks as "weaker" than the trions in the main manuscript.

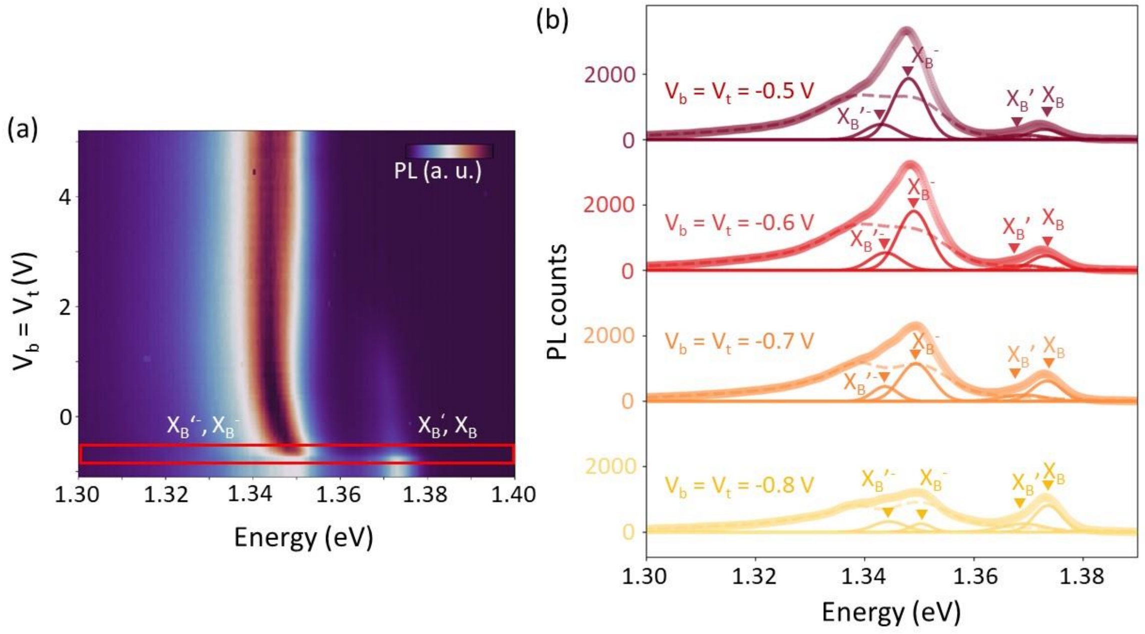

Fig. R2: (a) Doping-dependent photoluminescence map of dual-gated trilayer CrSBr. The red box shows the gate voltage region where trions start to appear. Select gate-dependent PL spectra in this region are shown in **(b)** with the corresponding Gaussian fits of the excitons (X_B and X_B') and trions (X_B^- and $X_B'^-$). The dashed lines show the fitted background.

Reviewer #1 (Remarks to the Author):

My comments have been addressed, and I recommend this work for publication.

We thank the reviewer for their time and valuable feedback on the manuscript, and for the recommendation for publication of our work.

Reviewer #2 (Remarks to the Author):

The authors have adequately addressed my last remaining comment. I recommend the publication of the revised manuscript in Nature Communications.

We thank the reviewer for their time and helpful feedback and their recommendation to publish the revised manuscript.

Reviewer #4 (Remarks to the Author):

I am satisfied with the authors' response and recommend publication in Nature Communications.

We thank the reviewer for the feedback and devoting their time in reviewing our work, and for the positive assessment towards publication of the manuscript.
